# MLL-Rearranged Acute Lymphoblastic Leukemias Activate BCL-2 through H3K79 Methylation and Are Sensitive to the BCL-2-Specific Antagonist ABT-199

**DOI:** 10.1016/j.celrep.2015.12.003

**Published:** 2015-12-17

**Authors:** Juliana M. Benito, Laura Godfrey, Kensuke Kojima, Leah Hogdal, Mark Wunderlich, Huimin Geng, Isabel Marzo, Karine G. Harutyunyan, Leonard Golfman, Phillip North, Jon Kerry, Erica Ballabio, Triona Ní Chonghaile, Oscar Gonzalo, Yihua Qiu, Irmela Jeremias, LaKiesha Debose, Eric O’Brien, Helen Ma, Ping Zhou, Rodrigo Jacamo, Eugene Park, Kevin R. Coombes, Nianxiang Zhang, Deborah A. Thomas, Susan O’Brien, Hagop M. Kantarjian, Joel D. Leverson, Steven M. Kornblau, Michael Andreeff, Markus Müschen, Patrick A. Zweidler-McKay, James C. Mulloy, Anthony Letai, Thomas A. Milne, Marina Konopleva

**Affiliations:** 1 Department of Leukemia, The University of Texas MD Anderson Cancer Center, Houston, TX 77030, USA; 2 Weatherall Institute of Molecular Medicine, Molecular Haematology Unit, NIHR Oxford Biomedical Research Centre Programme, University of Oxford, Headington, Oxford OX3 9DS, UK; 3 Division of Hematology, Respiratory Medicine and Oncology, Department of Internal Medicine, Faculty of Medicine, Saga University, Saga 840-8502, Japan; 4 Department of Medical Oncology, Dana-Farber Cancer Institute, Boston, MA 02215, USA; 5 Cancer and Blood Diseases Institute, Cincinnati Children’s Hospital Medical Center, Cincinnati, OH 45229, USA; 6 Department of Laboratory Medicine, University of California, San Francisco, San Francisco, CA 94143, USA; 7 Department of Biochemistry, Molecular and Cell Biology, University of Zaragoza, 50018 Zaragoza, Spain; 8 Division of Pediatrics, The University of Texas MD Anderson Cancer Center, Houston, TX 77030, USA; 9 Department of Physiology and Medical Physics, Royal College of Surgeons in Ireland, York House, Dublin 2, Ireland; 10 German Research Center for Environmental Health (GmbH), 85764 Neuherberg, Germany; 11 Department of Bioinformatics and Computational Biology, The University of Texas MD Anderson Cancer Center, Houston, TX 77030, USA; 12 Department of Oncology Development, AbbVie Inc., North Chicago, IL 60064, USA

**Keywords:** apoptosis pathways, leukemias, bcl-2 family members, MLL/AF4, DOT1L, H3K79 methylation

## Abstract

Targeted therapies designed to exploit specific molecular pathways in aggressive cancers are an exciting area of current research. *Mixed Lineage Leukemia* (*MLL*) mutations such as the t(4;11) translocation cause aggressive leukemias that are refractory to conventional treatment. The t(4;11) translocation produces an MLL/AF4 fusion protein that activates key target genes through both epigenetic and transcriptional elongation mechanisms. In this study, we show that t(4;11) patient cells express high levels of BCL-2 and are highly sensitive to treatment with the BCL-2-specific BH3 mimetic ABT-199. We demonstrate that MLL/AF4 specifically upregulates the *BCL-2* gene but not other BCL-2 family members via DOT1L-mediated H3K79me2/3. We use this information to show that a t(4;11) cell line is sensitive to a combination of ABT-199 and DOT1L inhibitors. In addition, ABT-199 synergizes with standard induction-type therapy in a xenotransplant model, advocating for the introduction of ABT-199 into therapeutic regimens for MLL-rearranged leukemias.

## Highlights

• *MLLr* ALL blasts express high levels of BCL-2, BAX, and BIM
• MLL/AF4 activates *BCL2* through H3K79 methylation
• *MLLr* ALL cells are exquisitely sensitive to BCL-2 antagonist ABT-199
• ABT-199 treatment synergizes with H3K79 methylation inhibitors on *MLLr* samples

## Graphical Abstract

**Figure:**
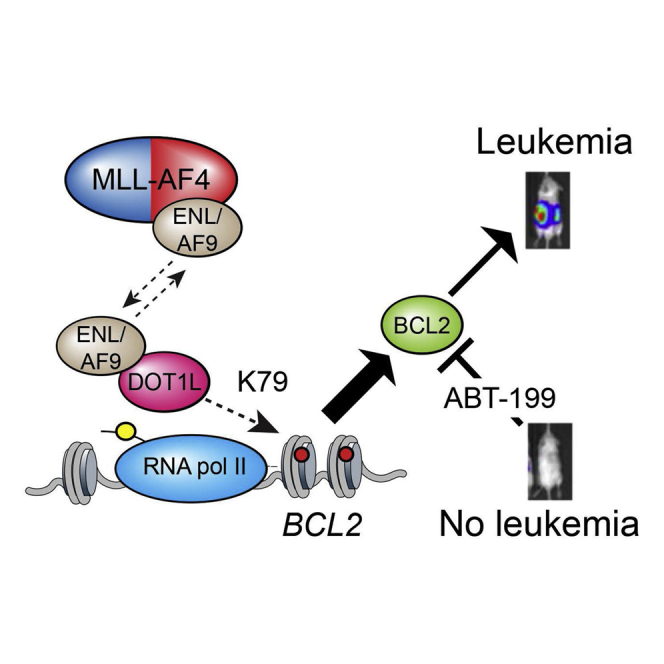

## Teaser

Therapies designed to exploit specific molecular pathways in aggressive cancers are an exciting area of research. Mutations in the MLL gene cause aggressive incurable leukemias. Benito et al. show that MLL leukemias are highly sensitive to BCL-2 inhibitors, especially when combined with drugs that target mutant MLL complex activity.

## Introduction

Mixed-lineage-leukemia (*MLL*) is one of the most frequently translocated genes (*MLL*-rearranged or *MLLr*) in hematologic malignancies and produces aggressive leukemias where more targeted therapeutic approaches are particularly needed. Translocation t(4;11)(q21;q23) generates *MLL/AF4* and *AF4/MLL* fusion products, both of which function as transcriptional activators. The role of AF4/MLL in t(4;11) leukemias is controversial, as it has transformation potential (Bursen et al., 2010) but is not expressed in all t(4;11) patients (Andersson et al., 2015). Conversely, the MLL/AF4 fusion protein is expressed in all t(4;11) patients, and knockdowns of MLL/AF4, even in the presence of AF4/MLL, are sufficient to stop t(4;11) leukemias from growing (Thomas et al., 2005).

t(4;11) leukemias are diagnosed mainly as precursor B cell acute lymphoblastic leukemia (B-ALL) in both infants, children, and adults, and they predict poor long-term outcomes, even with aggressive chemotherapy or therapy combined with stem cell transplantation (Beldjord et al., 2014, Dreyer et al., 2015, Pieters et al., 2007). t(4;11) leukemias have very few cooperating mutations, especially in infants (Andersson et al., 2015), suggesting that MLL/AF4 is the primary driver of continued leukemogenesis. Therefore, understanding the function of the MLL/AF4 fusion protein and the genes that it regulates will be essential for the development of targeted t(4;11) therapies.

BCL-2 family proteins mediate an intrinsic, mitochondrial apoptosis pathway. BCL-2, BCL-X_L_, and MCL-1 are anti-apoptotic BCL-2 family proteins, while BCL-2 homology 3 (BH3) proteins BIM, BID, BAD, NOXA, PUMA, and HRK are pro-apoptotic proteins that trigger cell death. Previous studies demonstrated high expression of *BCL-2* in *MLLr* pediatric ALL (Robinson et al., 2008). Using chromatin immunoprecipitation sequencing (ChIP-seq), we and others have detected direct binding of MLL/AF4 (Guenther et al., 2008, Wilkinson et al., 2013) to the *BCL-2* gene. This suggests, but does not completely establish, that MLL/AF4 and other fusion proteins could be the cause of increased BCL-2 levels through direct upregulation of *BCL-2* transcription. Supporting the potential importance of this observation, activity of the first-generation BCL-2 antagonists has indicated that BCL-2 inhibition could be exploited for *MLLr* leukemias (Robinson et al., 2008, Urtishak et al., 2013). ABT-199/GDC-0199 (venetoclax) is a BH3 mimetic that specifically targets BCL-2 while sparing BCL-X_L_, thus avoiding thrombocytopenia (Chonghaile et al., 2014, Pan et al., 2014, Souers et al., 2013, Vaillant et al., 2013, Vandenberg and Cory, 2013). ABT-199 has achieved promising anti-leukemia activity in patients with chronic lymphocytic leukemia (CLL) (Molica, 2015), and it has been reported to have preclinical activities in estrogen-receptor-positive breast cancer, acute myeloid leukemia (AML), early T cell progenitor leukemia, Myc-driven B cell lymphomas, and acute lymphoblastic leukemia (Alford et al., 2015, Chonghaile et al., 2014, Pan et al., 2014, Souers et al., 2013, Vaillant et al., 2013, Vandenberg and Cory, 2013).

Recruitment of P-TEFb (a heterodimer consisting of Cyclin T1 or T2 and the CDK9 kinase) and transcription elongation factors such as ENL and AF9 (Lin et al., 2010, Mueller et al., 2007, Yokoyama et al., 2010) are thought to be major ways in which MLL/AF4 activates gene targets. Other mechanisms have been proposed, including an ENL/AF9 direct interaction with the polycomb group (PcG) protein CBX8 (Maethner et al., 2013). In addition, ENL and AF9 interact directly with DOT1L (Biswas et al., 2011, Leach et al., 2013, Mohan et al., 2010), a histone methyltransferase that specifically methylates lysine 79 on histone 3. Since ENL or AF9 and DOT1L exist in a separate, distinct complex from MLL/AF4 (Biswas et al., 2011, Leach et al., 2013), it is unclear whether or how MLL/AF4 has any direct effect on recruitment of the DOT1L protein, but increased H3K79me2/3 levels are strongly associated with MLL/AF4 binding and with high levels of gene activation (Krivtsov et al., 2008).

In this study, we explored the dependence of ALL subtypes on BCL-2 family proteins and examined the antitumor efficacy of ABT-199 in ALL, with a special focus on the *MLLr* types. Our findings indicate that direct transcriptional upregulation of *BCL-2* by MLL/AF4 confers sensitivity to the selective BCL-2 antagonist ABT-199. We also show that MLL/AF4 promotes high levels of *BCL-2* expression by binding directly to the locus and keeping it active via maintenance of H3K79me2/3 without affecting P-TEFb recruitment. This MLL/AF4 regulatory activity is specific to *BCL-2* and has no effect on other BCL-2 family members. This led to the finding that the DOT1L inhibitors sensitize *MLLr* leukemias to BCL-2 inhibition with ABT-199. Importantly, we were also able to show that ABT-199 synergizes with standard-induction-type chemotherapeutic agents, suggesting that ABT-199 could be a useful addition to *MLLr* therapeutic regimens.

## Results

### t(4;11) ALL Is Associated with High Levels of BCL-2, BAX, and BIM

**Figure 1:**
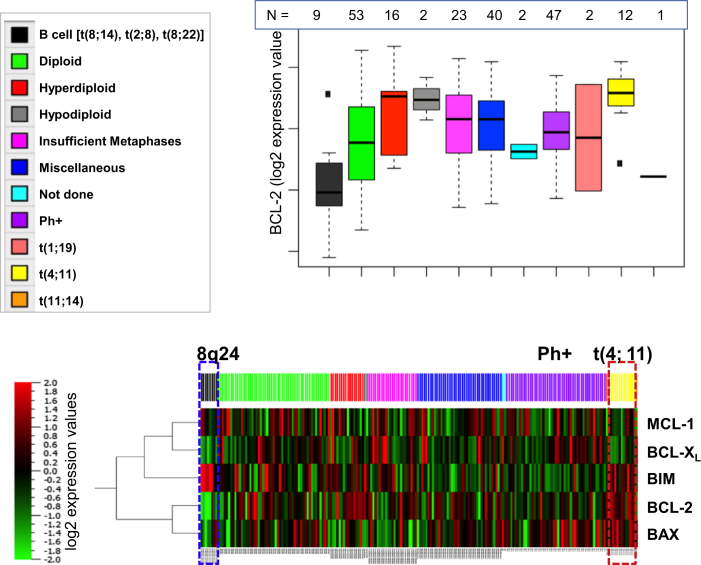
RPPA Profiling of BCL-2 Proteins in ALL, Showing Heatmaps of Differentially Expressed Proteins Based on Cytogenetics Black bars indicate 8q24 leukemia samples and their expression patterns are shown in the blue dashed line box. Yellow bars indicate t(4;11)-positive samples, and their expression patterns are shown in the red dashed line box. See also Figure S1.

Expression of 12 pro- and anti-apoptotic proteins was studied in 186 ALL cases by reverse-phase protein analysis (RPPA). Supervised clustering demonstrated distinct differences in acute lymphoblastic leukemia (ALL) with different cytogenetic characteristics (p < 0.005; false discovery rate [FDR], <0.2%). Patients with 8q24 (*CMYC*) translocation (Figure 1; n = 9) expressed low levels of BCL-2 and BAX while maintaining high expression of BIM and intermediate levels of MCL-1. No specific patterns were seen in t(9;22) (Ph+) ALL. Patients with t(4;11) (n = 12) expressed high levels of BCL-2, BAX, and BIM (Figure 1) but relatively low levels of BCL-X_L_ and MCL-1, although the latter differences did not reach statistical significance.

To investigate whether high BCL-2 protein levels in *MLLr* ALL is associated with high transcript levels of BCL-2, gene expression microarray data from three large cohorts of patients with ALL were analyzed (Figure S1): the St. Jude Children’s Research Hospital pediatric ALL clinical trial cohort; the Eastern Cooperative Oncology Group (ECOG) Clinical Trial E2993, and the Children’s Oncology Group (COG) Clinical Trial P9906. *BCL-2* mRNA expression was significantly higher in *MLLr* samples than in normal B cell controls in the St. Jude cohort (p = 0.015). *BCL-2* mRNA expression levels were significantly higher in the *MLLr* samples than in the E2A/PBX1 samples in all three cohorts (p = 0.015 for ECOG E2993, 0.0005 for COG P9906, and 0.002 for St. Jude’s) and higher than in molecularly undesignated B-ALL samples in the COG P9906 study (p = 0.01). These results suggest that *BCL-2* is highly expressed in t(4;11) and other *MLLr* ALL and, therefore, that BCL-2 is a potential therapeutic target in these ALL subtypes.

### MLL/AF4 Directly Controls Activation of the *BCL-2* Gene

**Figure 2:**
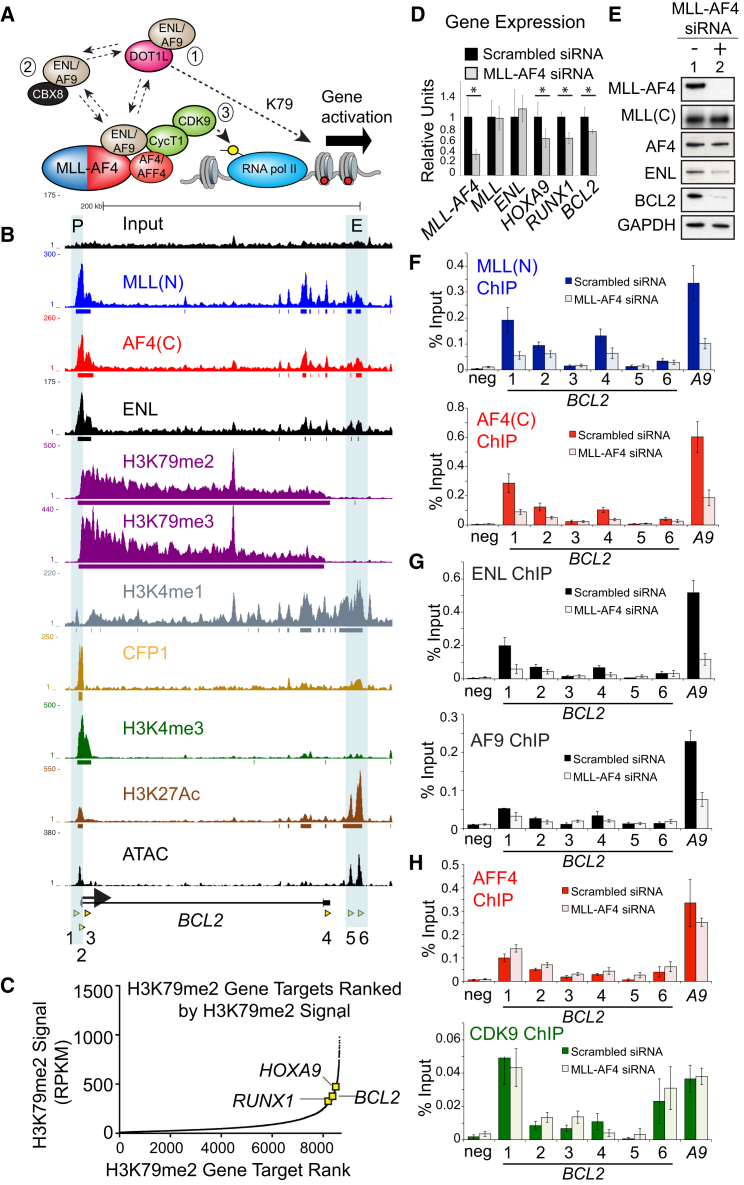
MLL-AF4 Binds to the *BCL-2* Gene and Keeps It Active (A) Schematic of MLL/AF4 direct interactions and mutually exclusive complexes (dotted arrows). Gene activation could occur (1) by promoting H3K79 methylation, (2) by inhibiting CBX8 activity, or (3) AF4/AFF4 direct recruitment of P-TEFb (ie. Cyclin T1 and CDK9) and serine 2 phosphorylation of RNA polymerase II. (B) ChIP-seq and ATAC-seq peaks in SEM cells across the *BCL-2* locus. PCR primers (1–6) used in subsequent experiments are shown as yellow arrowheads. P, promoter; E, enhancer. (C) *BCL-2* is ranked 8,381 out of 8,647 genes (top 5%) marked with H3K79me2 in SEM cells. (D) Real-time PCR for different targets in SEM cells treated with either a control (black bars) or an *MLL-AF4*-specific siRNA (gray bars). Signal was normalized to control treated cells and is the average of five independent knockdown experiments. Error bars indicate SD. ^∗^p < 0.02. (E) Western blots for the indicated proteins in SEM cells treated with either a control (−) or *MLL-AF4* specific (+) siRNA. (F–H) ChIP experiments (the average of three to five independent knockdowns) for MLL(N), AF4(C), ENL, AF9, AFF4, or CDK9 in SEM cells treated with either control (dark colored bars) or *MLL-AF4* siRNAs (light colored bars). PCR primers are as indicated in (A). *A9* = a primer set in the well-known MLL/AF4 target gene *HOXA9*, used as a positive control for ChIP. Error bars indicate SEM. See also Figure S2.

Confirming previously published data (Guenther et al., 2008, Wilkinson et al., 2013), ChIP-seq using an MLL N-terminal antibody (Ab) (MLLN) and an AF4 C-terminal Ab (AF4C) in the *MLL/AF4*-positive B-ALL cell lines SEM and RS4;11 shows that MLL/AF4 binds to the *BCL-2* locus (Figure 2B; Figure S2A). ChIP-seq for specific MLL/AF4 complex components (summarized in Figure 2A) shows that ENL binding closely matches the profile of MLL/AF4 binding, while H3K79me2/3 creates a broad domain across the locus (Figure 2B). Comparable to two canonical MLL/AF4 target genes, *BCL-2* is a typical MLL/AF4 target gene in that it is marked with very high levels of H3K79me2 (Figure 2C). A potential downstream enhancer—identified by its enrichment for H3K4me1, H3K27Ac, and the presence of ATAC sequencing (ATAC-seq) peaks—is also bound by the MLL/AF4/ENL complex (Figure 2B, blue shaded region marked with an E). A peak of H3K4me3 and binding of the SET1 complex (CFP1) are detected at the *BCL-2* promoter (Figure 2B), suggesting that the locus could also be regulated by H3K4 methyltransferase complexes.

For a functional analysis of MLL/AF4 activity, SEM and RS4;11 cells were treated with unique *MLL/AF4*-specific small interfering RNAs (siRNAs) (Thomas et al., 2005). MLL/AF4 siRNA knockdowns led to a reduction of *BCL-2* gene expression that was comparable to that of *HOXA9* and *RUNX1* (Figure 2D; Figure S2B) and also resulted in reduced BCL-2 protein levels in replicate experiments (Figures 2E and S2C). MLL/AF4 siRNA treatment also reduced MLL/AF4 binding to *BCL-2* (Figures 2F and S2D) without affecting wild-type MLL levels (Figures 2D, 2E, and S2B) or wild-type MLL or AF4 binding to BCL-2 (Figure S2E). Conversely, wild-type MLL knockdowns had no effect on *BCL-2* expression (Figure S2F), indicating that MLL/AF4 directly activates *BCL-2* while wild-type MLL is dispensable for *BCL-2* activation in t(4;11) cells.

MLL/AF4 knockdowns were associated with reduced ENL binding to *BCL-2* in both SEM and RS4;11 cells (Figures 2G and S2D), but there was only a marginal effect on AF9 binding, especially compared to *HOXA9* (Figure 2G). ENL mRNA levels are unaffected by MLL/AF4 knockdowns (Figure 2D), but ENL protein levels are reduced (Figure 2E), suggesting that the direct interaction between MLL/AF4 and ENL may somehow stabilize the ENL protein. In contrast to the results observed with ENL, no effect was seen on the binding of AFF4, CDK9, or Cyclin T1 in SEM cells (Figures 2H and S2G), suggesting that MLL/AF4-mediated activation of *BCL-2* does not occur through P-TEFb recruitment and stabilization. Although CBX8 binding was easily detected at *HOXC8* (a known Polycomb target in SEM cells), no change in CBX8 binding was seen at *BCL-2* (Figure S2H). Instead, MLL/AF4 knockdowns were associated with a loss of DOT1L binding at *BCL-2* (Figure S2I). Together, these results suggest that neither pathway 2 nor pathway 3 (see Figure 2A) are important components of MLL/AF4-mediated regulation of *BCL-2* but that MLL/AF4, instead, stabilizes both ENL and DOT1L binding (pathway 1) at *BCL-2*.

### MLL/AF4 Controls *BCL-2* Gene Activation by Promoting Increased H3K79me2/3 Levels

**Figure 3:**
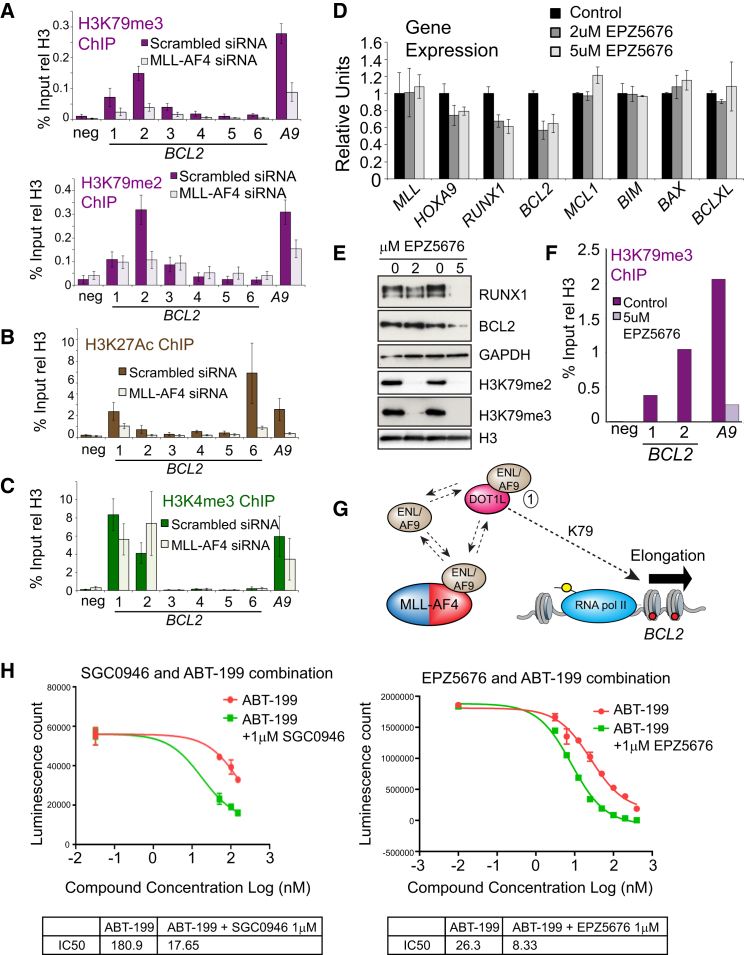
MLL-AF4 Keeps *BCL-2* Active by Promoting H3K79me2/3 (A–C) ChIP experiments (average of four independent knockdowns) for H3K79me2, H3K79me3, H3K27Ac, or H3K4me3 in SEM cells treated with either control (dark bars) or *MLL-AF4* siRNAs (light bars). PCR primers are as in Figure 1. Error bars indicate SEM. rel, relative; neg, negative. (D) SEM cells treated with 2 μM or 5 μM EPZ5676 for 7 days were subjected to real-time RT-PCR with the primers/probe sets indicated. Error bars indicate SD of four PCR replicates. (E) Western blots of SEM cells treated with 2 or 5 μM EPZ5676 for 7 days. (F) H3K79me3 ChIP at the *BCL-2* and *HOXA9* loci in 5 μM EPZ5676-treated SEM cells. (G) A proposed model where MLL/AF4 stabilizes ENL protein levels and creates a local concentration of ENL that allows for dynamic exchange between an MLL-AF4:ENL complex and a DOT1L:ENL complex, potentially increasing H3K79me3 levels at the locus. (H) SEM cells were co-treated with 1 μM of DOT1L inhibitors SGC0946 or EPZ5676 and increasing concentrations of ABT-199 (50 nM, 100 nM, and 150 nM) for 4 days (SGC0946) or 7 days (EPZ5676). Error bars indicate SEM. See also Figure S3.

To explore MLL/AF4 function further, we performed ChIP for several different histone marks in MLL/AF4 knockdowns. Consistent with the observed loss of DOT1L binding, both H3K79me2 and me3 levels are reduced across *BCL-2* (Figure 3A). We also observed reductions of H3K27Ac, especially at the downstream enhancer region of *BCL-2* (Figure 3B), whereas no significant changes in H3K4me3 levels were seen (Figure 3C).

To determine whether reduction of H3K79me2/3 levels alone could impact expression of *BCL-2* or other gene targets, SEM cells were treated with either 2 μM or 5 μM of the DOT1L inhibitor EPZ5676 for 7 days. The MLL/AF4 target genes *HOXA9*, *RUNX1*, and *BCL-2* (but not any other BCL-2 family members) all showed reduced expression with the 2- and 5-μM treatment at day 7 (Figure 3D), and this correlated with a loss of H3K79me2/3 globally and at the *BCL-2* and *HOXA9* loci (Figures 3E and 3F). Although both the 2-μM and 5-μM treatments impacted *BCL-2* expression, only the 5-μM treatment produced an observable reduction in BCL-2 protein levels (Figure 3E), comparable to that seen when *BCL-2* is directly targeted with siRNAs that can disrupt leukemic growth (Figures S3A and S3B). Together with the data in the previous section, these results indicate that the primary way that MLL/AF4 controls activation of *BCL-2* is through maintaining H3K79Me2/3 levels (see Figure 3G for a summary).

To determine whether DOT1L activity and BCL-2 inhibition were cooperative, we examined growth-inhibitory activity of two structurally distinct DOT1L inhibitors, SGC0946 and EPZ5676, combined with the selective BCL-2 inhibitor ABT-199. Consistent with our aforementioned results using 2 μM EPZ5676, treatment with 1 μM EPZ5676 had very little detectable effect on BCL-2 family protein levels (Figure S3C). However, a combined blockade of DOT1L and BCL-2 demonstrated deeper growth-inhibitory effects in t(4;11) SEMK2 cells (Figure 3H) but not in the non-*MLLr* cell line Nalm-6 (Figure S3D).

**Figure 4:**
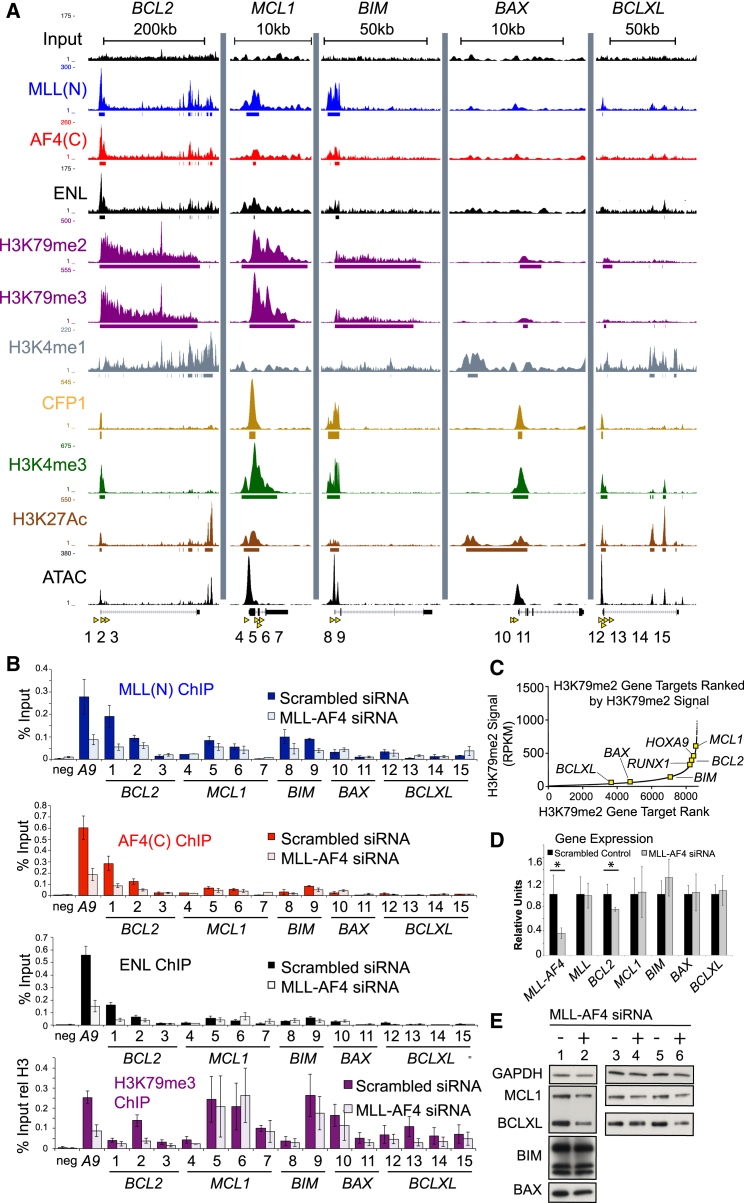
MLL/AF4 Does Not Directly Activate Other BCL-2 Family Genes (A) ChIP-seq and ATAC-seq peaks in SEM cells at the loci indicated. *BCL-2* tracks are also shown for comparison purposes. PCR primers (1–15) used in subsequent experiments shown by yellow arrowheads. (B) ChIP experiments (average of three to five independent knockdowns) in SEM cells treated with either control (dark colored bars) or *MLL-AF4* siRNAs (light colored bars). PCR primers are as in (A). *BCL-2* and *HOXA9* (*A9*) data are from Figure 2 and included for comparison purposes. Error bars indicate SEM. (C) The same H3K79me2 ranking graph as in Figure 2C, with *BCL-2* family genes added for comparison purposes. (D) Real-time PCR of samples from Figure 2D for *BCL-2* family genes. *MLL-AF4*, *MLL*, and *BCL-2* expression data are from Figure 2D and included for comparison purposes. Error bars indicate SD. ^∗^p < 0.02. (E) Western blots for the indicated proteins in SEM cells treated with either a control (−) or *MLL-AF4*-specific (+) siRNA. Results shown are from three different biological replicates.

In SEM cells, MLL/AF4 is bound to both *MCL-1* and *BIM*, but not *BAX* or *BCL-2L1* (Figures 4A and 4B), and *BCL-2* family members are associated with a range of H3K79me2/3 levels (Figures 4A–4C). MLL/AF4 knockdowns have little effect on ENL or H3K79me3 levels at these loci (Figure 4B), and there is no effect on the expression levels of *MCL-1*, *BIM*, *BAX*, or *BCL-2L1* (Figures 4D and 4E). Together, these data suggest that MLL/AF4 specifically activates *BCL-2* by promoting increased H3K79me2/3 levels, while other BCL-2 family members are not dependent on MLL/AF4 or H3K79me2/3 for their expression, even though BIM and BAX are both highly expressed in t(4;11) patient samples.

### BH3 Profiling Demonstrates BCL-2 Dependence of *MLLr* Primary ALL

**Figure 5:**
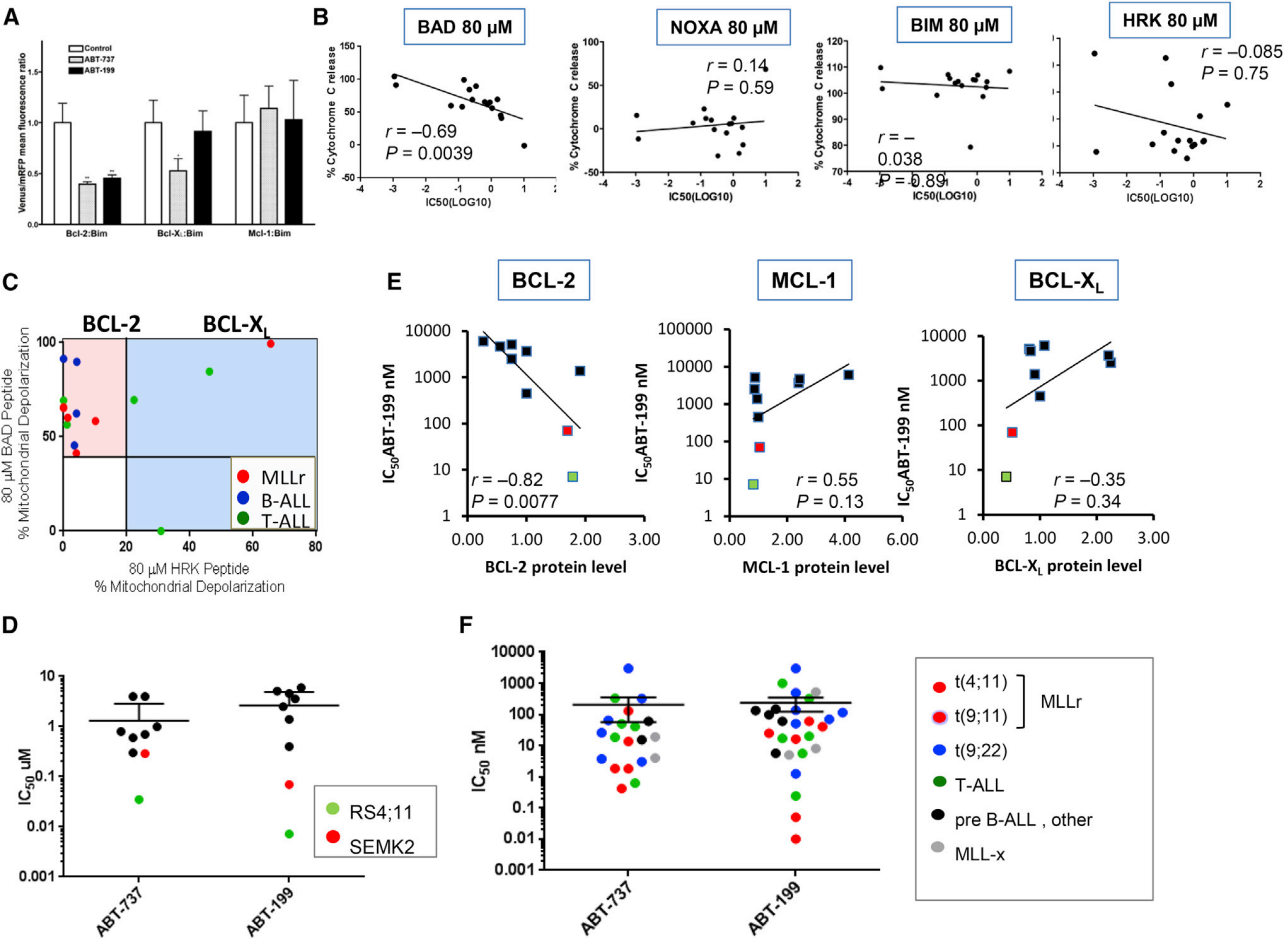
BH3 Profiling Reveals BCL-2 Dependence in Primary ALL (A) BiFC analysis of interactions of anti-apoptotic proteins with BIM. Results are expressed as the fold change induced by ABT-199/ABT-737 in Venus/RFP (red fluorescent protein) ratio and are the mean ± SEM of three to six independent experiments. ^∗^p < 0.05; ^∗∗^p < 0.01. (B) Levels of cytochrome *c* release from mitochondria of ALL cells exposed to the indicated BH3 peptides were correlated with cell viability IC_50_ values for ABT-199. (C) Cytochrome *c* release induced by BAD versus HRK peptides. The pink area represents probable BCL-2 dependence, and the blue area represents BCL-X_L_ dependence. (D) IC_50_ values for ABT-199 and ABT-737 in ALL cell lines. *MLL*-rearranged ALL cell lines are shown in green (RS4;11) or red (SEMK2). ALL cell line cells were treated with ABT-737 or ABT-199 for 48 hr, and the IC_50_ values calculated on the basis of viable (i.e., Annexin V-/PI-) cell numbers determined by flow cytometry. (E) Sensitivity to ABT-199 correlates with endogenous BCL-2 protein levels but not with BCL-X_L_ levels in ALL cell lines. Spearman correlations were calculated based on protein expression levels relative to an internal control (β-actin) and then normalized to levels in NALM-6 cells. (F) Primary ALL cells (n = 19) were treated with ABT-737 or ABT-199 for 24 hr, and the IC_50_ values were calculated as described earlier. MLLx, *MLL*-rearranged xenograft samples. See also Figure S4.

ABT-199 is a BCL-2-selective inhibitor recently shown to be active in ALL (Alford et al., 2015). In bimolecular fluorescence complementation (BiFC) assays (Figure 5A; Vela et al., 2013), ABT-199 inhibited BCL-2/BIM interactions, and ABT-737 inhibited the interactions of BCL-2 and BCL-X_L_ with BIM, according to the known specificity of these inhibitors. BH3 profiling is a technique that identifies the BCL-2 protein family addictions of cancer cells based on the selective binding of BH3 proteins to specific anti-apoptotic BCL-2 family proteins (Certo et al., 2006, Chonghaile et al., 2014, Pan et al., 2014). Using ALL blast mitochondria from 16 samples consisting of primary ALL cells or patient-derived xenografts, we found a statistically significant correlation between mitochondrial sensitivity to BAD BH3 peptide (indicating BCL-2, BCL-X_L_, or BCL-W dependence) and cell viability determined by half-maximal inhibitory concentration (IC_50_) values of ABT-199 (Figure 5B; Table S2). No correlation was found for the mitochondrial response to BCL-X_L_-selective HRK, MCL-1-selective NOXA, or pan-BCL-2 family BIM BH3 peptides (Figure 5B). Similar correlations were obtained in a different set of adult and pediatric B-ALL samples treated with ABT-737 (Figure S4A). Next, we plotted sensitivity to the BAD BH3 peptide against sensitivity to the HRK peptide (Figure 5C). Five of the six *MLLr* samples and additional four B-ALL samples showed BCL-2 dependency (shaded area).

### *MLLr* Cells Expressing High Levels of BCL-2 Are Susceptible to ABT-199-Induced Apoptosis In Vitro and to a Combination of ABT-199 with Chemotherapy

Our gene transcription studies and functional BH3 profiling predict that *MLLr* ALL will be particularly dependent on BCL-2 for survival. In a series of genetically diverse ALL cell lines, two t(4;11)-positive cell lines, RS4;11 and SEM-K2, exhibited high sensitivity to ABT-199 (Figures 5D and S4B). BCL-2 protein expression was highest in *MLLr* RS4;11 and SEMK2 together with the pro-B ALL cell line REH; and BCL-2, but not MCL-1 or BCL-X_L_, expression correlated with sensitivity to ABT-199 (r = −0.82, p = 0.008) (Figures 5E and S4C). In a panel of 24 primary ALL samples, 92% were sensitive to ABT-199 (IC_50_ < 1 μM; Figures 5F and S4D), and 78% were sensitive to ABT-737. Interestingly, all five *MLLr* samples—four t(4;11) cases and one t(9;11) case—had ICs_50_ ≤0.1 μM. Western blot analysis of ALL blasts (n = 9) confirmed high BCL-2 protein levels (Figure S4E). High sensitivity of primary B-ALL (non-MLL) samples to BCL-2 inhibition was validated in a separate cohort of adult and pediatric samples treated with ABT-199 or dual BCL-2/BCL-X_L_ inhibitor ABT-263 for 8 hr (Figure S4F).

**Figure 6:**
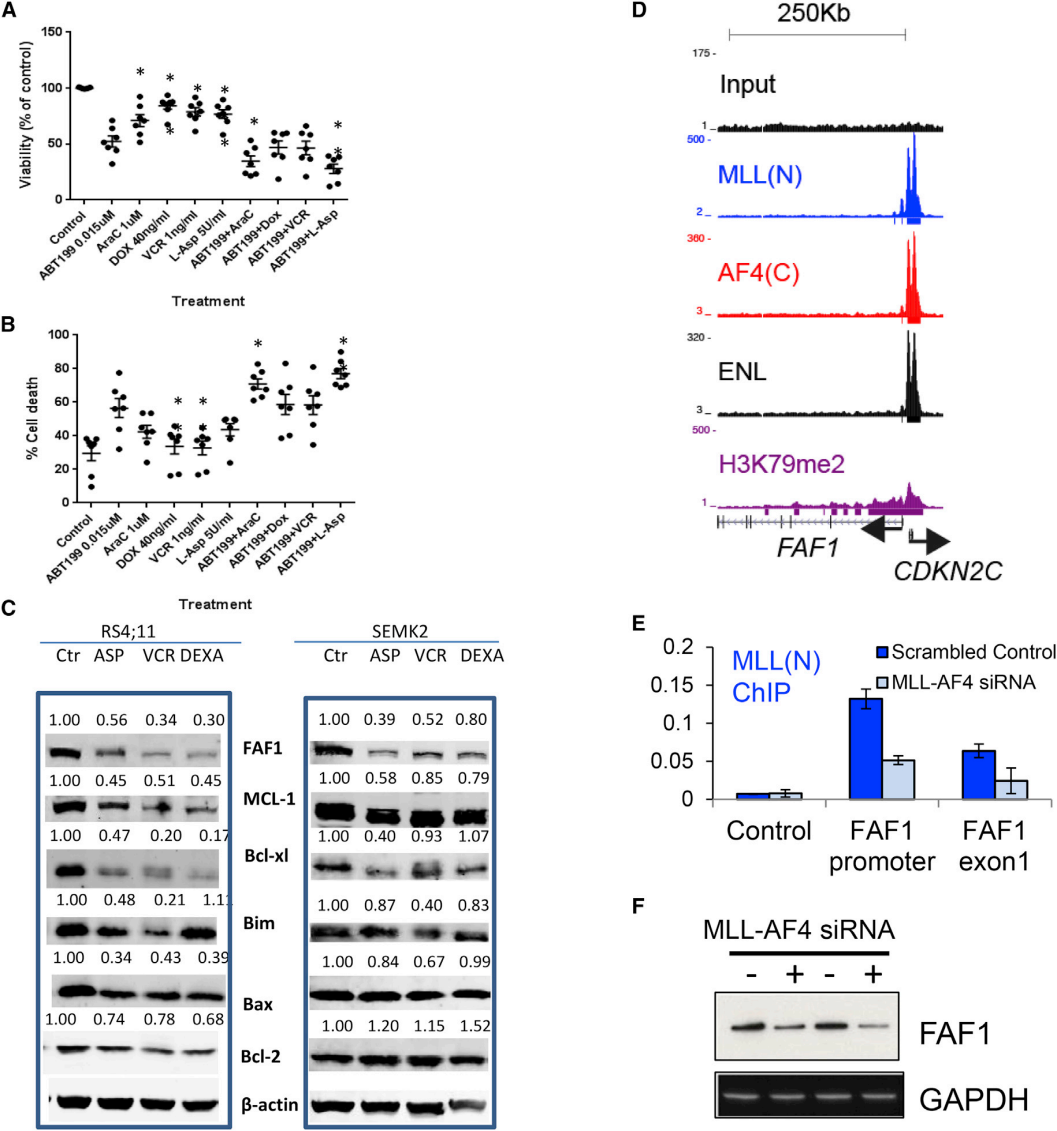
ABT-199 in Combination with Chemotherapy Exhibits Cytotoxic Activity against Primary ALL Cells (A and B) ALL primary samples (n = 6) and one sample from a patient with t(4;11) biphenotypic leukemia were treated with each single reagent or in combination with ABT-199 at the following concentrations: ABT-199, 0.015 μM; AraC, 1 μM; DOX, 40 ng/ml; VCR, 1 ng/ml; and L-ASP, 5 U/ml. At 24 hr, viability (A) and cell death (B) were determined by Annexin V and 7-AAD staining. Each dot represents one sample. Error bars indicate SEM. ^∗^p < 0.05. (C) Western blot analysis of RS4;11 or SEMK2 cells left untreated (Ctr) or treated for 48 or 24 hr, respectively, with: L-ASP (ASP), 2 U or 5 U (RS4;11 or SEMK2, respectively); VCR, 5 ng/ml; or DEXA, 1 μM. Representative blot of one of the three experiments that yielded similar results is shown. (D–F) MLL/AF4 binds to the *FAF1* gene (D), and MLL/AF4 siRNA treatment reduces MLL-AF4 binding to *FAF1* (E) and reduces FAF1 protein levels (western blot, F). Error bars in (E) represent SEM for three independent knockdown experiments. See also Figure S5 and Table S3.

In ALL cell lines (REH, SEMK2, and RS4;11), a combination of ABT-737 or ABT-199 with chemotherapy agents (vincristine [VCR], doxorubicin [DOX], dexamethasone [DEXA], cytarabine [AraC], L-asparaginase [L-ASP]) was commonly synergistic (Table S3), with best responses observed upon combination with L-ASP (combination index values, <0.01). In six primary ALL samples, each combination produced significantly greater cytotoxic activity (Figures 6A and 6B). Consistent with the cell line data, the combination of L-ASP with ABT-199 exhibited the greatest effect.

Treatment with L-ASP in ALL patients is able to induce apoptosis specifically in lymphoblast cells. We reasoned that the nature of the L-ASP/ABT-199 synergistic interaction could be due to enhancement of apoptotic pathways in MLL/AF4 cells. Consistent with this, western blot analysis of RS4;11 or SEMK2 cells treated with L-ASP, VCR, or DEXA showed that each treatment significantly reduced the levels of anti-apoptotic proteins MCL-1 and BCL-X_L_ and the apoptotic factor FAS-associated factor 1 (FAF1; Figure 6C). Interestingly, MLL/AF4 binds and activates *FAF1* expression (Figures 6D–6F), and although FAF1 does not appear to contribute to leukemic growth (Figure S5), MLL/AF4 could contribute to high levels FAF1 protein in the cell and sensitivity to apoptosis induction.

### ABT-199 Attenuates Tumor Growth of *MLLr* precursor-B ALL In Vivo and Enhances Anti-leukemia Effects of Standard Chemotherapy

**Figure 7:**
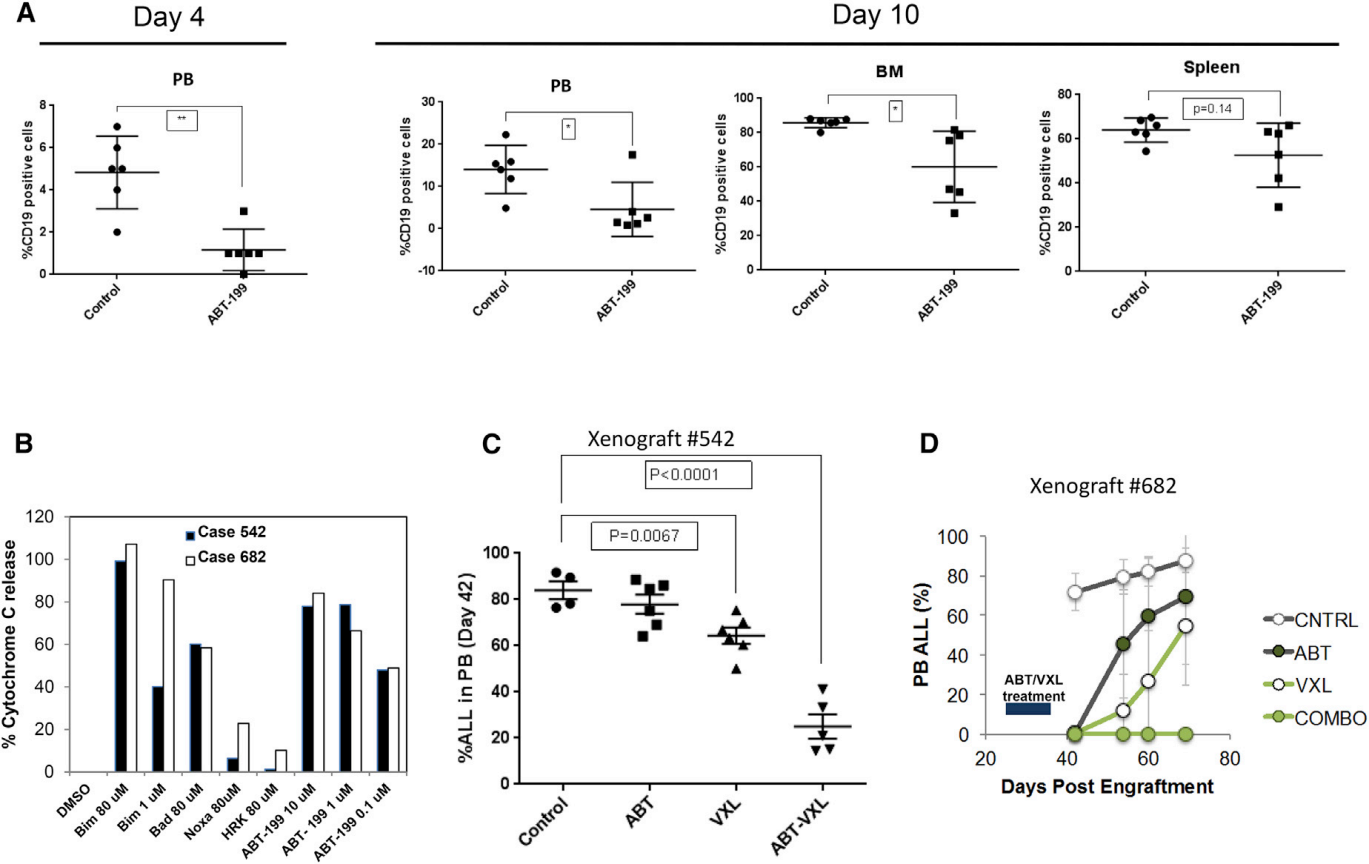
ABT-199 Attenuates Tumor Growth of *MLLr* Pre-B ALL and Interacts Synergistically with Induction-type Chemotherapy to Eradicate Patient-Derived ALL Cells In Vivo (A) NSG mice were injected intravenously with ICN3 xenograft cells generated from a pediatric patient with relapsed *MLL*-rearranged pre-B-ALL. At day 45 post-injection, mice were randomized into two treatment groups (n = 6 per arm): Vehicle only; ABT-199 at 100 mg/kg/day. Leukemia burden expressed as percent human CD19^+^ cells is shown in peripheral blood (PB) on days 4 and 10 after treatment initiation and in BM and spleen on day 10. ^∗^p < 0.05; ^∗∗^p < 0.01. (B) BH3 profiling of primary derived xenografts #542 and #682. Cytochrome *c* release in response to various concentrations of BH3 peptides and ABT-199. (C and D) Leukemia cells from two patients with t(4;11) ALL (#542 and #682) were injected into NRG mice via tail vein. On day 24 post-engraftment, mice were randomly divided into cohorts to receive VXL (VCR, DEXA, and L-ASP), ABT-199 alone, VXL+ABT-199 combination; or vehicle controls (n = 6 per arm). (C) Percentage of circulating ALL cells on day 42 post-engraftment in case #542. (D) Time course changes in percentages of circulating ALL cells in case #682. Leukemia progression was evaluated by determining the percentage of circulating human CD45-positive cells across different treatment groups. Error bars indicate SD. See also Figure S6.

Next, we investigated the anti-leukemic efficacy of ABT-199 in *MLLr* ALL in vivo using primary ALL xenograft models. In a highly aggressive primary xenograft ICN3 model, there was a marked reduction of circulating CD19-positive cells after 4 days of treatment initiated on day 45 post-cell injection (Figure 7A). Despite high levels of blasts in bone marrow (BM) on day 10 due to rapid disease progression, half of the treated mice appeared to benefit (Figure 7A). In the ALL-236-GFP model, treatment with ABT-199 for 10 days reduced tumor burden measured by bioluminescence or circulating GFP(+) cells (Figures S6A and S6B).

Next, the anti-leukemic effects of ABT-199 in combination with an induction-type regimen, VXL, comprising VCR, L-ASP, and DEXA, were evaluated in mice injected with cells from two t(4;11)-positive ALL patients (#542 and #682). Leukemic cells from both of these patients were found to be BCL-2 dependent by BH3 profiling, with sample #682 more sensitive to BIM peptide (Figure 7B). Leukemia burden (human CD45-positive cells) was determined serially in peripheral blood for case #682. Because case #542 showed a more aggressive disease course, leukemia burden was determined only on day 42 of treatment.

In mice engrafted with #542, ABT-199 alone had minimal effects on peripheral blood leukemia burden (Figure 7C). VXL alone modestly reduced leukemia burden by 23% (p = 0.007). Unexpectedly, the combination of ABT-199 with VXL reduced leukemia burden >70% (p < 0.0001; Figure 7C), revealing striking synergy in this ALL PDX model.

In case #682 mice, both VXL and ABT-199 showed anti-leukemia effects (Figure 7D), consistent with higher sensitivity by BH3 profiling. On day 60 (Figure S6C), all six control mice carried circulating human CD45-positive cells (mean ± SD, 81.8% ± 7.1%), while one mouse from each of the six treated with ABT-199 and the six treated with VXL was leukemia free. Strikingly, circulating human CD45-positive cells were not detected in any of the mice in the combination cohort up to day 69, although positive cells were detected again on day 103 (data not shown). Two mice in the combination-treatment group died from chemotherapy-related toxicity and infection but were leukemia-free on autopsy.

## Discussion

Lymphoid malignancies utilize the anti-apoptotic BCL-2 family proteins to maintain viability under conditions of oncogenic stress. Because of this dependence, the leukemia cells are susceptible to inhibition of anti-apoptotic BCL-2 family proteins. It has been reported that the BCL-2/BCL-X_L_ inhibitors ABT-737, ABT-263, and ABT-199 induce rapid and robust apoptosis in ALL cells, both in vitro (Alford et al., 2015, Del Gaizo Moore et al., 2008, High et al., 2010) and in human-derived xenografts (Suryani et al., 2014). In this context, we investigated the expression of BCL-2 family members in a large series of ALL patient samples by proteomic profiling. In accordance with molecular heterogeneity of ALL, the proteomic profiles were widely dispersed but closely associated with cytogenetic abnormalities. t(4;11) ALL was closely associated with high levels of BCL-2, BAX, and BIM. BCL-2 has been previously implicated in the pathogenesis of *MLLr* leukemias, and disruption of MLL-fusion-driven *BCL-2* expression has been proposed as a major mechanism of action for the bromodomain inhibitor I-BET151 (Dawson et al., 2011). Examination of publicly available gene expression datasets demonstrated that pediatric *MLLr* ALL expresses high *BCL-2* mRNA levels, consistent with previous findings in a smaller subset of pediatric ALL patients (Robinson et al., 2008). In this study, we demonstrate a direct role for MLL/AF4 in maintaining *BCL-2* expression, providing a plausible explanation for dependency of *MLLr* ALL cells on BCL-2 anti-apoptotic activity.

Interestingly, although we show that *BCL-2* expression is MLL/AF4 dependent, other *BCL-2* family members such as *MCL-1*, *BIM*, *BAX*, and *BCL-2L1* show no direct dependence on MLL/AF4 for their expression. This is despite the fact that we detect low levels of MLL/AF4 binding and H3K79me2 at both the *MCL1* and the *BIM* genes. These results highlight the fact that it is important to functionally validate ChIP-seq experiments, since the presence of binding is not necessarily functionally relevant. Importantly, we also show that wild-type MLL has no direct role in activating *BCL-2* in t(4;11) cells, indicating that *BCL-2* overexpression is an MLL/AF4-specific regulatory event. Therefore, treatment with *BCL-2* inhibitors targets a direct pathway of the MLL/AF4 driver mutation, indicating that this could be a specific vulnerability of these poor-prognosis t(4;11) leukemias.

Although the correlation between MLL/AF4 binding and H3K79 methylation levels has been observed before (Guenther et al., 2008, Krivtsov et al., 2008, Wilkinson et al., 2013), there was previously little direct evidence that MLL/AF4 acted primarily through H3K79me2/3 levels. Instead, past work has suggested that MLL/AF4 functions primarily by recruiting a large transcription elongation complex that includes AFF4, P-TEFb, ENL, AF9, and other proteins (Lin et al., 2010, Mueller et al., 2007, Yokoyama et al., 2010). It is unknown what the exact role of H3K79me2/3 is, but recent work has suggested that it functions, in part, by disrupting SIRT1-mediated silencing (Chen et al., 2015). DOT1L inhibitors were able to reduce *BCL-2* expression, while *MCL1*, *BIM*, *BAX*, and *BCL-2L1* show almost no sensitivity to DOT1L inhibitors. This suggests that the role of H3K79me2/3 is very gene and context specific and underscores the importance of future work designed to further explore the function of this important histone mark and the complexes that regulate it.

The AF4 protein interacts directly with ENL, but the ENL:AF4 interaction and the ENL:DOT1L interactions are mutually exclusive, so how could MLL/AF4 cause recruitment of DOT1L and increased H3K79me2 levels? Structural analysis of the AF9-DOT1L interaction has shown that AF9 (and, by extension, ENL) interacts with AF4 and DOT1L through the same intrinsically disordered domain (Kuntimaddi et al., 2015, Leach et al., 2013). Intrinsic disorder allows for rapid association kinetics and the possibility of a rapid, dynamic exchange of binding partners between ENL:AF4 and ENL:DOT1L. Thus, it is possible that MLL/AF4 has a direct impact on binding of DOT1L by increasing the local concentration of ENL and/or AF9 proteins. To fully understand this possibility, further work is needed that studies the dynamic interactions of these protein complexes in vivo.

Our observation that treatment of SEM cells with DOT1L inhibitors appears to cooperate with ABT-199 treatment provides an interesting proof of principle that DOT1L inhibitors could potentially be used to sensitize *MLLr* leukemias to treatment with ABT-199. DOT1L inhibition affects gene targets other than *BCL-2* (e.g., *HOXA9* and *RUNX1*); therefore, the nature of this cooperative effect could be due to a general inhibition of a range of different MLL/AF4 targets rather than one or a few MLL/AF4 targets. However, this provides an important proof of principle that combining inhibitors that target MLL/AF4 complex activity can be used in combination with inhibitors of important MLL/AF4 target gene products.

Through BH3 profiling, we demonstrate the predominant dependence of *MLLr* B-lineage ALL cells on BCL-2, as mitochondrial sensitivity to BCL-2-selective BAD peptide was more potent than BCL-X_L_-selective HRK, MCL-1-selective NOXA, and non-specific BIM peptide. Further, the robust response observed upon treatment with the BAD peptide showed excellent correlation with mitochondrial depolarization achieved in primary ALL blasts with ABT-199, confirming the on-target BCL-2-dependent activity of this agent. Notably, an additional four B-ALL and two out of five precursor T cell ALL (T-ALL) samples likewise demonstrated BCL-2 dependence. These data indicate that several phenotypically and genetically distinct ALL subtypes utilize BCL-2 as a primary pro-survival mechanism (Chonghaile et al., 2014).

Our results suggest that the presence of t(4;11) may predict response to ABT-199 in ALL. We found that the human *MLLr* B-ALL cell lines expressed the highest levels of BCL-2 protein and exhibited the greatest sensitivity to ABT-199 among the cell lines tested. Among the primary B-ALL samples tested, all *MLLr* were highly sensitive. In addition to its single-agent efficacy, ABT-199 showed beneficial results when combined with induction-type conventional chemotherapy in PDX models of B-lineage *MLLr* ALL established from patient-derived leukemia cells. In four separate in vivo experiments, short-term ABT-199 treatment (7–10 doses) had only transient anti-leukemia effects, yet it profoundly enhanced efficacy of the VXL chemotherapy regimen. The synergistic response between ABT-199 and chemotherapy treatment is likely to be complex and not due to a single factor. Previous studies have documented synergistic anti-leukemia efficacy of dual BCL-2/X_L_ inhibitor ABT-737 in ALL cells, including xenograft models upon combination with VXL, the regimen used here (Kang et al., 2007); this synergy was attributed to the ability of L-ASP to downregulate MCL-1 protein levels (High et al., 2010). Further, anti-mitotic agents have been shown to synergize with BH3 mimetics and reduce MCL-1 protein levels (Chen et al., 2011, Leverson et al., 2015, Tan et al., 2011, Wong et al., 2012), at least in part through phosphorylation and proteasomal degradation of MCL-1 during mitotic arrest (Wertz et al., 2011). Our data in *MLLr* cell lines support these findings, demonstrating downregulation of both MCL-1 and BCL-X_L_ by these agents. The regulation of the *FAF-1* locus by MLL/AF4 presents another interesting possibility. High FAF1 protein levels are able to enhance apoptosis, and FAF1 protein is degraded upon induction of apoptosis (Menges et al., 2009). This suggests that MLL/AF4-mediated FAF1 overexpression could sensitize t(4;11) cells to induction of apoptosis by factors such as L-ASP, as long as the anti-apoptotic activity of BCL-2 is also inhibited by ABT-199. This could further explain synergy between BCL-2 antagonist and standard chemotherapeutic agents used in ALL regimens seen in our studies.

In summary, our findings demonstrate that *BCL-2* is a direct target of the rearranged MLL in ALL cells, translating into BCL-2 dependence and vulnerability to selective, on-target BCL-2 inhibition by the clinically active agent ABT-199. These findings strongly advocate introduction of ABT-199, which recently demonstrated impressive efficacy in CLL trials, into the clinical armamentarium of ALL therapy.

## Experimental Procedures

### Reagents

ABT-199 and ABT-737 were provided by AbbVie.

### Cell Lines, siRNA, Primary Samples, and Cultures

Cell lines used for this study are detailed in the Supplemental Experimental Procedures. MLL/AF4 siRNA experiments were performed as described by Thomas et al. (2005), with differences noted in the Supplemental Experimental Procedures. All animal experiments were reviewed by institutional animal committees. All work with human samples was approved by the institutional review board at the University of Texas MD Anderson Cancer Center and the Dana-Farber Cancer Institute. Primary samples were derived from patients with ALL after informed consent was obtained in accordance with institutional guidelines set forth by the MD Anderson Cancer Center and Dana-Farber Cancer Institute. Clinical sample information is summarized in Table S1.

### Western Blot Analysis

Western blot analysis was performed as previously described (Pan et al., 2014, Wilkinson et al., 2013). Antibody sources are listed under Supplemental Experimental Procedures.

### RPPA

Expression of pro- and anti-apoptotic BCL-2 family proteins was studied in 186 patients diagnosed with ALL by RPPA. Antibodies used are listed in Table S4. The methodology and validation of RPPA are described elsewhere (Kornblau et al., 2011).

### BiFC

Interactions between anti-apoptotic proteins BCL-2, BCL-X_L_, and MCL-1 and pro-apoptotic proteins BIM and NOXA were studied by BiFC (Vela et al., 2013).

### ChIP Assays and ChIP-Seq

ChIP and ChIP-seq experiments were performed as described in the Supplemental Experimental Procedures and as previously described (Milne et al., 2009, Wilkinson et al., 2013).

### Intracellular BH3 Profiling of Primary ALL Cells

Intracellular BH3 profiling on primary ALL cells was performed as previously described (Pan et al., 2014).

### In Vivo Murine Leukemia Models

ALL-236-GFP/LUC cells generated from pre-B-ALL with t(4;11) (Terziyska et al., 2012) and ICN3 xenograft cells generated from a child with relapsed *MLLr* pre-B-ALL (Duy et al., 2011) were injected intravenously into nonobese diabetic-severe combined immunodeficiency (NOD SCID)/IL2Rγ-KO (NSG) mice. Mice were treated with vehicle (Phosal 50 PG/polyethylene glycol [PEG]40/ethanol, 60/30/10 v/v) or ABT-199 (100 mg/kg/day) by oral gavage. For the combination model, an induction-type regimen consisting of VCR, L-ASP, and DEXA (VXL) (Szymanska et al., 2012) was used in NOD Cg-Rag1^tm1Mom^ IL2rg^tm1Wjl^/SzJ (NRG) mice.

### Statistical Analysis

Data were analyzed by the two-tailed Student’s t test or the Mann-Whitney test, if appropriate. Differences were considered statistically significant at p < 0.05. Unless otherwise indicated, data are expressed as mean ± SD.

Additional details on experimental procedures are included in the Supplemental Experimental Procedures.

## Author Contributions

J.M.B.: designed, performed, and analyzed experiments; wrote and edited manuscript. K.K.: wrote and edited manuscript. L.H., I.M., and R.J.: designed, performed, and analyzed experiments; edited manuscript. M.W., K.G.H., L. Golfman, T.C., O.G., E.B., Y.Q., E.O., L. Godfrey, P.N., J.K., E.B., P.Z., and E.P.: designed, performed, and analyzed experiments. H.G., K.R.C., and N.Z.: analyzed data. L.D. and H.M.: performed and analyzed experiments. D.A.T., M.A., S.O., H.M.K.: edited manuscript. J.D.L., M.M., and I.J.: provided reagents and edited manuscript. S.M.K.: designed experiments and analyzed data. P.A.Z.-M., T.A.M., J.C.M., and A.L.: designed and analyzed experiments and edited manuscript. T.M. and M.K.: designed and analyzed experiments; wrote and edited manuscript.

## Conflicts of Interest

J.D.L. has ownership interest in AbbVie, Inc.; M.A. has commercial research support from Daiichi-Sankyo, has received honoraria from the speakers’ bureau of Tetralogic, and is a consultant/advisory board member of Amgen and Eutropics; M.K. has received a commercial research grant from AbbVie, Inc., and is a consultant/advisory board member of the same; A.L. is a consultant/advisory board member of AbbVie Pharmaceuticals. No potential conflicts of interest were disclosed by the other authors.

## Accession Numbers

The accession number for the data discussed in this publication is GSE: GSE74812 (http://www.ncbi.nlm.nih.gov/geo/query/acc.cgi?acc=GSE74812).

## Supplemental Information

Document S1. Supplemental Experimental Procedures, Figures S1–S6, and Tables S1–S4

Document S2. Article plus Supplemental Information
