# Supplementary material for: MLL-Rearranged Acute Lymphoblastic Leukemias Activate BCL-2 through H3K79 Methylation and Are Sensitive to the BCL-2-Specific Antagonist ABT-199
Source: Cell Rep. 2015 Dec 17;13(12):2715–27. doi: 10.1016/j.celrep.2015.12.003 (PMC4700051; doi:10.1016/j.celrep.2015.12.003)
Supplement: Document S1. Supplemental Experimental Procedures, Figures S1–S6, and Tables S1–S4 [file mmc1.pdf]

Cell Reports

Supplemental Information

MLL-Rearranged Acute Lymphoblastic Leukemias Activate BCL-2 through H3K79 Methylation and Are Sensitive to the BCL-2-Specific Antagonist ABT-199

Juliana M. Benito, Laura Godfrey, Kensuke Kojima, Leah Hogdal, Mark Wunderlich, Huimin Geng, Isabel Marzo, Karine G. Harutyunyan, Leonard Golfman, Phillip North, Jon Kerry, Erica Ballabio, Triona Ní Chonghaile, Oscar Gonzalo, Yihua Qiu, Irmela Jeremias, LaKiesha Debose, Eric O'Brien, Helen Ma, Ping Zhou, Rodrigo Jacamo, Eugene Park, Kevin R. Coombes, Nianxiang Zhang, Deborah A. Thomas, Susan O'Brien, Hagop M. Kantarjian, Joel D. Leverson, Steven M. Kornblau, Michael Andreeff, Markus Müschen, Patrick A. Zweidler-McKay, James C. Mulloy, Anthony Letai, Thomas A. Milne, and Marina Konopleva

Table S1, Related to Experimental Procedures. Primary samples information

Arbitrary sample #	Diagnosis	Source	% Blasts	Cytogenetics	Assay
1	Rel/Refr T-ALL	PB	95	del(5q), del(6q), and i(17q)	BH3 prof, comb, WB
2	Rel/Refr B-ALL	PB	91	insufficient metaphases	BH3 prof, single treat, WB
3	Rel T-ALL	PB	9	del(6)	BH3 prof, single treat
4	Refr T-ALL	BM	25	del(5q), del(7q) and +21	BH3 prof, single treat, WB
5	Refr preB-ALL	PB	100	t(4;11)	single treat, WB
6	New B-ALL	PB	94	t(9;22)	BH3 prof, comb
7	T-ALL	PB	95	inv(17)	BH3 prof
8	T-ALL	PB	74	ND	BH3 prof, WB
9	New B-ALL	BM	94	t(9;22)	BH3 prof, comb, WB
11	Rel/Refr B-ALL	PB	90	Diploid	BH3 prof, comb, WB
12	ICN3 (P9)	Xenograft		MLL-rearranged ALL	BH3 prof
13	ICN13	Xenograft		MLL-rearranged ALL	BH3 prof
14	ALL-236	Xenograft		MLL-rearranged ALL	BH3 prof
15	ALL 542	Xenograft		t(4;11)	BH3 prof
16	ALL 682	Xenograft		MLL-rearranged ALL	BH3 prof
17	PS 2013-25	Xenograft		N/A	BH3 prof
18	New PreB-ALL	BM	94%	46,XY,del(9)(q13),add(10)(q24)[3]/46,XY[4]	BH3 prof, Single treat
19	New PreB-ALL	BM	88%	45,XX,der(7)t(7;9)(p11;q11),-9,t(9;22)(q34;q11.2)[12]/46,idem,+der(22), t(9;22)[cp2]/46,XX[6], Ph+ ALL,	BH3 prof, Single treat
20	New PreB-ALL	BM	89%	46,XY,t(4;19)(q31;q13)[20].nuc ish(ABLx2)[100]	BH3 prof, Single treat
21	New PreB-ALL	BM	81%	46,XY[20].nuc ish(ABL1,BCR)x2[100]	BH3 prof, Single treat
22	New B-ALL	BM	74%	45,XX,-7,t(9;22;16)(q34;q11;q24)[cp19]/46,XX[1]	BH3 prof, Single treat
23	Pediatric New B-ALL	BM	97%	56XY,+X,dup(1)(q21q42),+4,+6,+9,+10,+14,+14,+17,+21,+21	BH3 prof, Single treat
24	Pediatric New B-ALL	BM	96%	54-55XX,+X,+4,+4,i(7)9q10),+15,i(17)(q10),-19,+21,+22,+1-3 marker	BH3 prof, Single treat
25	Pediatric New B-ALL	BM	90%	53-55XXX,add(1)(q4?2),+4,+6,+10,+14,+17,+21,+21	BH3 prof, Single treat
26	Pediatric New B-ALL	BM	89%	55XX,+X,+4,+6,+10,+14,+18,+18,+19,+21,+21	BH3 prof, Single treat
27	Pediatric New B-ALL	BM	95%	Normal	BH3 prof, Single treat

28	Pediatric New B-ALL	BM	80%	51XX,+X,+4,-7,+9,+14,+der(7)t(7;17)(q11.2;p11.2)+21	BH3 prof, Single treat
29	Pediatric New B-ALL	BM	73%	normal	BH3 prof, Single treat
30	Rel preB-ALL	PB	69%	t(4;11)	Single treat
31	New preB-ALL	PB	90%	Diploid	Single treat
32	Rel/refr T-ALL	PB	99%	del(9)	Single treat
33	Rel/refr preB-ALL	PB	56%	t(2;9)(p23;p22),add(7)(q36),del(13)(q12q14),del(17)(p 11.2),+mar[19]	Single treat, WB
34	Rel/Refr T-ALL	PB	95%	del(5q), del(6q), and i(17q)	Single treat
35	New preB-ALL	PB	62%	t(9;11)	Single treat
36	Rel/refr preB-ALL ph(-)	PB	31%	t(4;11)	Single treat
37	Rel/Refr preB-ALL	PB	95%	t(9;22)	Single treat
38	Rel preB-ALL	PB	86%	insufficient metaphases	Single treat
39	New preB-ALL. Ph(+)	PB	87%	t(9;22)	Single treat
40	New preB-ALL	PB	46%	Diploid	Single treat
41	Rel/Refr T-ALL	BM	6%	Diploid	Single treat
42	New preB-ALL	PB	58%	t(9;22)	Single treat
43	Refr T-ALL	PB	86%	del (6)	Single treat
44	New preB-ALL	PB	83%	t(9;22)	Single treat, WB
45	Rel/Refr AML (MLL)	PB	80%	46,XY,t(6;11)(q27;q23)[20]; MLL	Comb
46	Rel/Refr B-ALL	BM	97%	t(14;19)(q32;p13.1),del(17)(p11.2),-20,+mar[9]	Comb, WB
47	New B-ALL	PB	87%	t(9;22)(q34;q11.2)	Comb
48	New B-ALL	BM	63%	t(4;11)(q21;q23) (FISH-MLL+)	Comb

BH3 prof: BH3 profiling; single treat: single treatment; WB: western blot; comb: combination

Table S2, Related to Figure 5. BH3 profiling of primary ALL samples and primary derived xenografts.

	B-ALL				T-ALL					MLL-r					
Sample number	6	9	11	2	1	3	4	7	8	12	13	14	15	16	17
DMSO	0	0	0	0	0	0	0	0	0	0	0	0	0	0	0
Bim 80 uM	102.89	101.73	102.22	105.2	106.93	108.41	104.48	103.87	98.7	79.33	105.66	105.02	99.17	107.1	104.34
Bim 1 uM	98.82	97.01	53.53	95.77	69.92	103.65	83.05	91.81	73.78	18.45	99.29	53.62	40.14	90.41	57.19
Bim 0.3 uM	67.9	65.12	17.07	47.55	17.69	86.8	26.25	64.55	25.49	-3	81.71	16.85	20.81	47.03	-7.84
Bim 0.1 uM	20.97	17.8	11.25	8.96	12.04	66.84	4.51	36.06	0.17	-9.21	44.56	6.47	14.16	16.65	-11.95
Bim 0.03 uM	9.98	-6.19	4.64	2.12	12.23	47.38	2.95	17.36	-8.65	-5.62	25.26	3.22	6.11	10.12	-10.68
Bad 80 uM	89.8	91.37	45.42	62.41	56.48	-0.69	69.3	84.66	69.53	65.73	99.41	65.32	60.11	58.38	41.33
Bad 30 uM	91.7	93.02	49.48	62.31	53	7.47	73.59	89.57	76.14	65.97	99.27	66.21	64.23	71.48	47.09
Bad 8 uM	86.6	93	53.56	57.25	52.5	12.96	71.66	90.91	74.06	65.16	98.9	60.99	72.9	66.56	49.41
Noxa 80uM	-31.15	-11.75	1.57	5.54	12.35	68.16	-1	10.09	-28.31	-4.76	11.92	6.07	6.52	22.83	-18.49
HRK 80 uM	4.18	-4.19	3.43	4.12	1.12	30.88	-3.55	46.21	22.34	-9.09	65.6	0.02	1.34	10.12	4.07
Puma 80 uM	93.75	95.23	58.86	90.19	79.67	85.16	62.8	91.87	77.41	45.55	96.21	74.94	47.06	62.31	49.29
Puma 8 uM	82.84	91.33	38.91	66	56.68	58.67	12.41	88.89	76.27	32.03	93.64	49.93	45.67	76.29	36.02
Puma 0.8 uM	28.03	47.7	8.81	17.67	18.39	26.81	76.58	50.96	43.41	-3.78	65.85	9.91	12.08	28.13	-11.31
ABT-199 10 uM	86.77	94.12	77.68	78.83	81.49	-21.1	81	96.55	77.74	70.28	99.88	73.34	77.89	84.09	74.9
ABT- 199 1 uM	73.25	91.76	27.2	50.12	40.57	7.82	65.97	70.47	40.64	57.22	84.08	49.93	78.68	66.39	39.94
ABT-199 0.1 uM	28.62	78.5	20.44	28.39	23.62	12.24	39.04	39.83	15.93	33.62	51.22	29.54	48.01	48.86	10.05
ABT-737 1 uM	82.79	93.29	44.95	54.53	35.73	11.52	67.12	85.51	73.3	49.81	91.79	56.15	64.31	64.21	52.04
ABT-737 0.1 uM	73.17	86.91	32.19	43.13	23.81	7.02	49.02	71.79	54.07	32.72	85.2	32.56	50.22	59.5	20.42
Puma2a 80 uM	-15.5	-14.93	10.32	-14.27	11.42	-4.94	-24.11	8.23	46.4	-13.24	0	5.85	-0.14	-1.33	-14.33

Cytochrome C release in response to various concentrations of BH3 peptides and ABT-737 or ABT-199 in B-ALL samples, T-ALL samples and primary derived MLLr xenografts. For clinical information, please refer to Table S1.

Table S3, Related to Figure 6. Combination indices for indicated drug combinations at ED50, ED75 and ED90.

Cell line	Combination	ED50	ED75	ED90
REH	VCR/ABT-737	0.38	0.29	0.22
	VCR/ABT-199	0.73	0.40	0.22
	AraC/ABT-737	2.50	1.02	0.41
	AraC/ABT-199	0.40	0.22	0.12
	DEXA/ABT-737	1.8	2.2	2.8
	DEXA/ABT-199	1.44	3.50	10.58
	L-ASP/ABT-737	<0.001	<0.001	<0.001
	L-ASP/ABT-199	0.06	0.05	0.04
	DOX/ABT-737	0.23	0.12	0.06
	DOX/ABT-199	0.12	0.07	0.04
SEMK2	VCR/ABT-737	0.8	0.6	0.4
	VCR/ABT-199	0.8	0.4	0.2
	AraC/ABT-737	>10	>10	0.4
	AraC/ABT-199	>10	1.1	0.02
	DEXA/ABT-737	0.5	0.4	0.3
	DEXA/ABT-199	0.4	0.2	0.1
	L-ASP/ABT-737	0.002	0.004	0.008
	L-ASP/ABT-199	0.003	0.002	0.001
	DOX/ABT-737	0.7	0.6	0.5
	DOX/ABT-199	0.8	0.4	0.2
RS4;11	VCR/ABT-737	1.00	0.84	0.70
	VCR/ABT-199	0.63	0.57	0.52
	AraC/ABT-737	>10	0.35	0.34
	AraC/ABT-199	>10	0.12	0.26
	DEXA/ABT-737	6.23	6.01	5.80
	DEXA/ABT-199	0.32	0.42	0.55

Table S4, Related to Experimental Procedures. List of antibodies used in RPPA

**Antibody List**	**Company**	**cat#**
BAX	Cell Signaling	2772
BCL-2	DAKO	M0887
BIM	Epitomics	1036-1
BCL-X_L	BD biosciences	559027
MCL-1	Cell Signaling	2762S

Figure S1, Related to Figure 1, Association of high BCL-2 protein expression in MLLr with transcript levels of *BCL2.*

Adult ALL: ECOG E2993

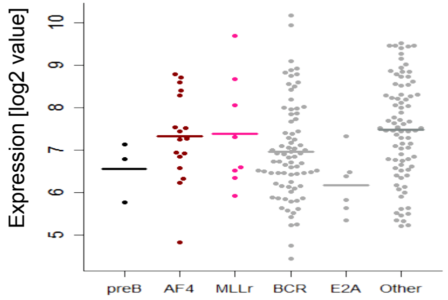

P values							
BCL2_ave	BCR	E2A	rMLL	AF4	other rMLL	others	preB
BCR	1	0.0669	0.0958	0.1146	0.4264	0.0036	0.6617
E2A	0.0669	1	**0.0145**	**0.0197**	0.0593	0.0091	0.5476
other rMLL	0.0958	0.0145	1	0.9285	0.8830	0.5147	0.2186
AF4	0.1146	0.0197	0.9285	1	0.8419	0.5746	0.1789
**MLL.8**	0.4264	0.0593	0.8830	0.8419	1	0.6966	0.4970
others	0.0036	0.0091	0.5147	0.5746	0.6966	1	0.1784
preB	0.6617	0.5476	0.2186	0.1789	0.4970	0.1784	1

191 ECOG (E2993):
BCR/ABL1: (78)
E2A/PBX1: (6)
MLL (25) : MLL/AF4 (17) + other MLLr (8)
Other B-ALL: (82)
normal preB: (3)

GEO#: GSE34861

ECOG E2993 clinical trail, n=191
Huimin Geng, et al. Cancer Discovery 2012

Childhood ALL: COG P9906

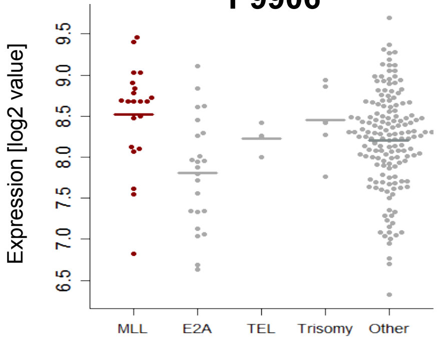

P values					
BCL2_ave	E2A	MLLr	TEL	others	Trisomy
E2A	1	**0.0005**	0.2415	0.0062	0.0615
**MLLr**	0.0005	1	0.1719	0.0101	0.8010
TEL	0.2415	0.1719	1	0.9188	0.5714
Trisomy	0.0615	0.8010	0.5714	1	1.0000
Other	0.0062	**0.0101**	0.9188	1.0000	1

COG P9906 clinical trail, n=207
Rechard Harney et al, Blood 2010

207 COG (P9906):
MLLr: (21)
E2A/PBX1: (23)
Other B-ALL: (155)
TEL/AML1: (3)
Trisomy 4 or 10: (5)

GEO#: GSE28460

Childhood ALL: St. Jude ALL

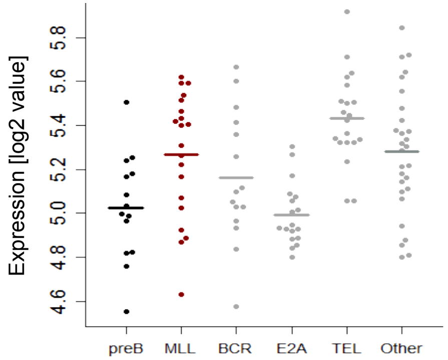

P values						
BCL2_ave	BCR	E2A	MLL	TEL	others	preB
BCR	1	0.0437	0.3299	0.0068	0.2193	0.2340
E2A	0.0437	1	**0.0019**	0.0000	0.0003	0.6395
**MLL**	0.3299	0.0019	1	0.1081	0.8766	0.0150
TEL	0.0068	0.0000	0.1081	1	0.0499	0.0000
others	0.2193	0.0003	0.8766	0.0499	1	0.0088
preB	0.2340	0.6395	**0.0150**	0.0000	0.0088	1

St Jude:
BCR: (15)
E2A: (18)
MLL: (20)
TEL: (20)
Hyperdip: (17)
Other B-ALL: (28)
normal preB: (14)

St Jude ALL:http://www.stjuderesearch.org/data/ALL3. (no GEO number, but raw data can be downloaded from the above website). # normal preB (14): http://franklin.et.tudelft.nl/.

Figure S2,Related to Figure 2,MLL/AF4 controls activation of the *BCL-2* gene.

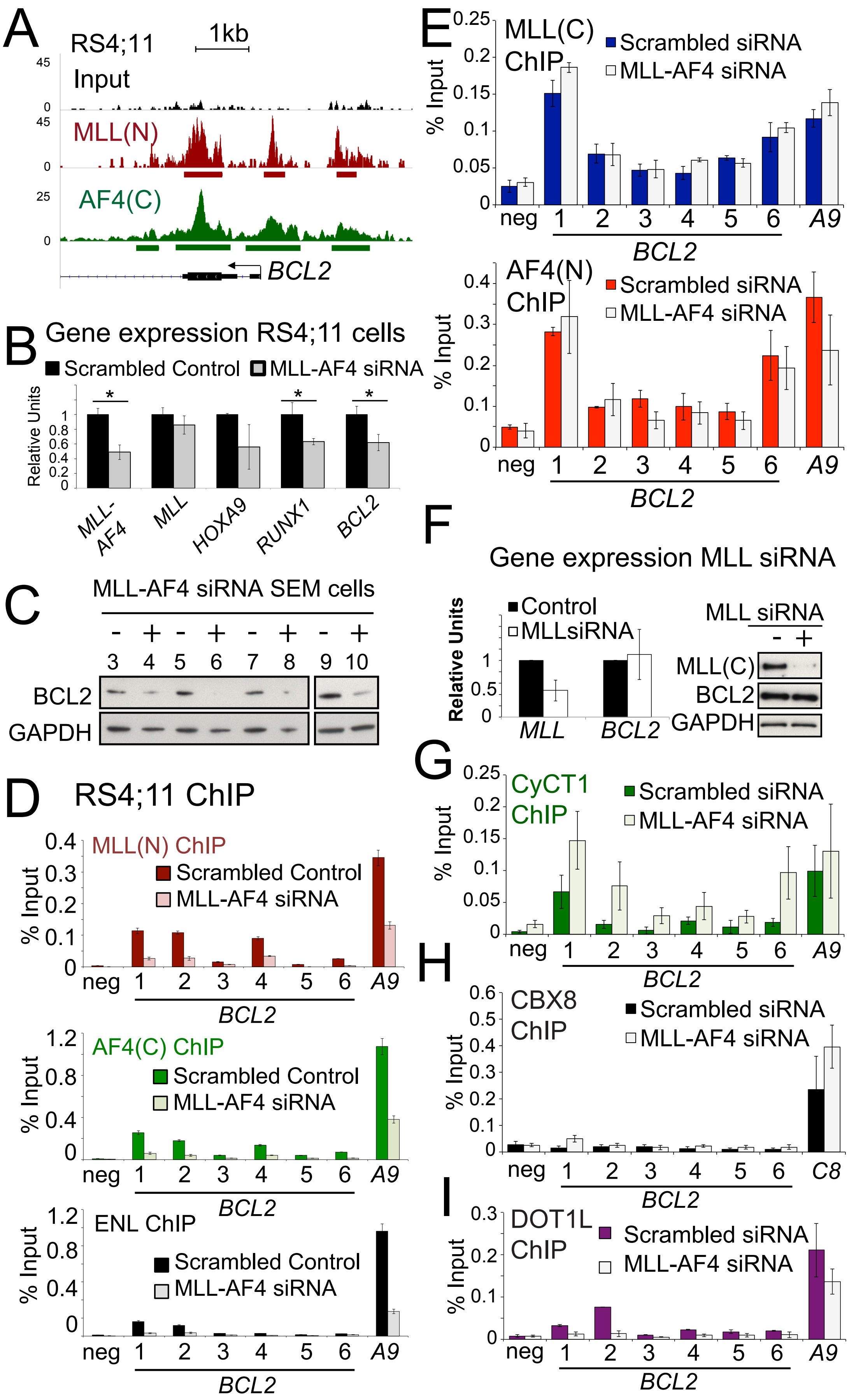

Figure S3, Related to Figure 3, Loss of BCL-2 reduces SEM cell growth and the DOT1L inhibitor EPZ5676 has minimal effect on the survival of Nalm-6 cells.

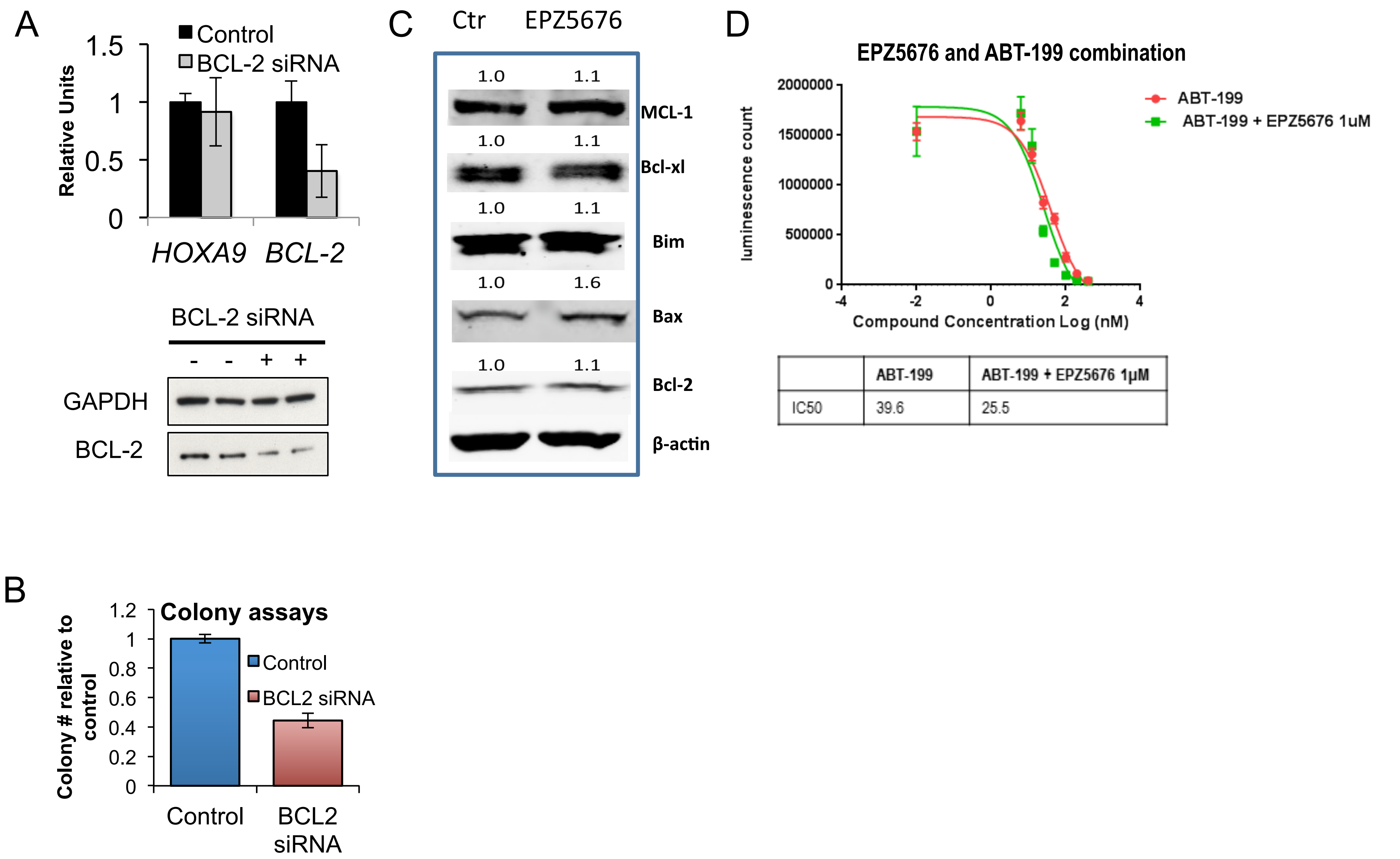

	ABT-199	ABT-199 + EPZ5676 1μM
IC50	39.6	25.5

Figure S4, related to Figure 5, ABT-737 or ABT-199 in ALL cell lines and primary samples.

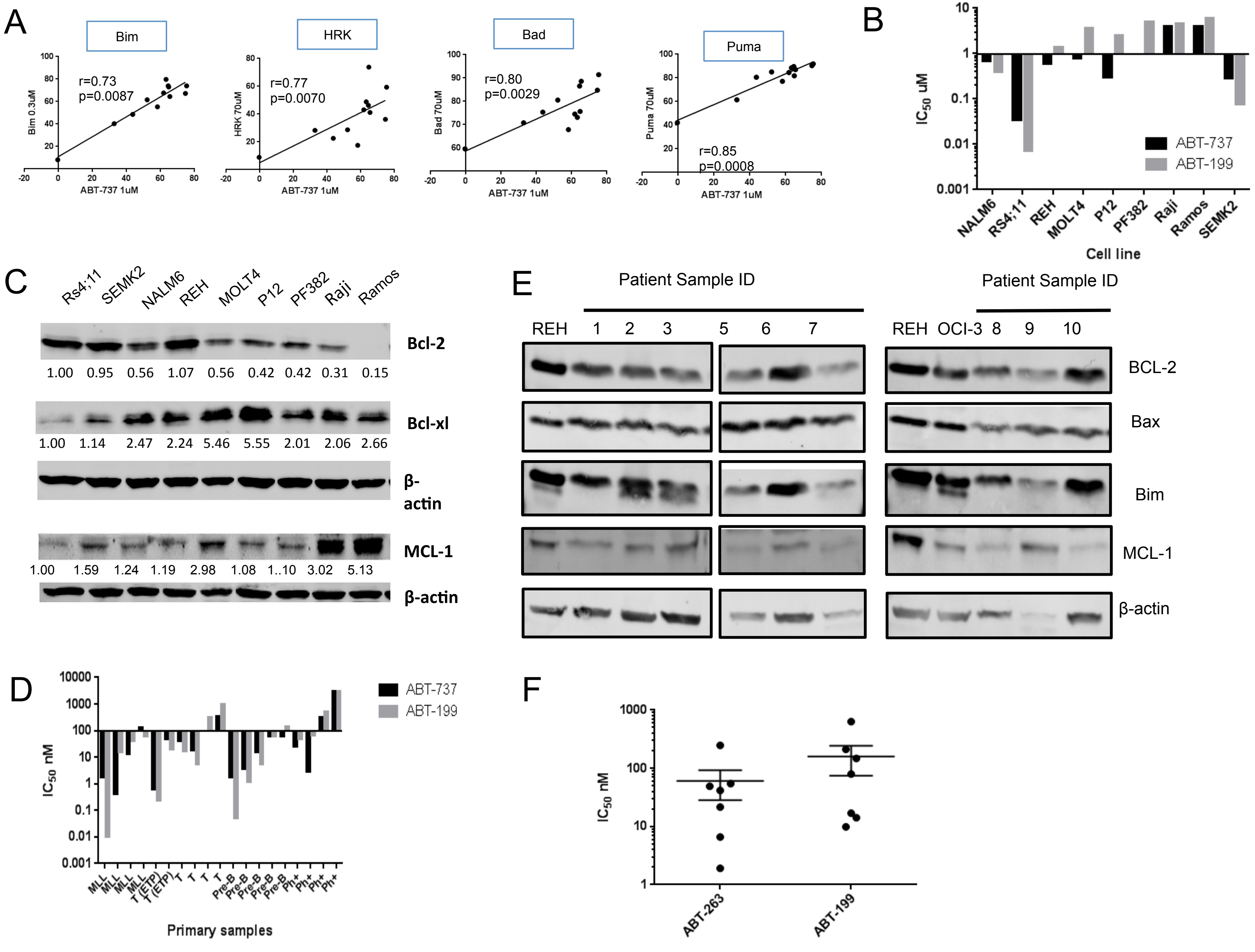

Figure S5, related to Figure 6, FAF1 is a target of MLL/AF4 but does not contribute to leukemic growth.

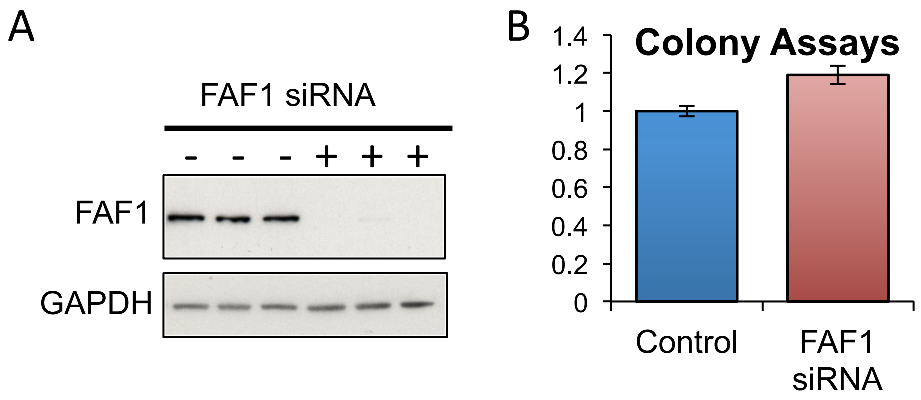

Figure S6, related to Figure 7, ABT199 Inhibits Leukemia Progression in ALL xenograft Model In Vivo.

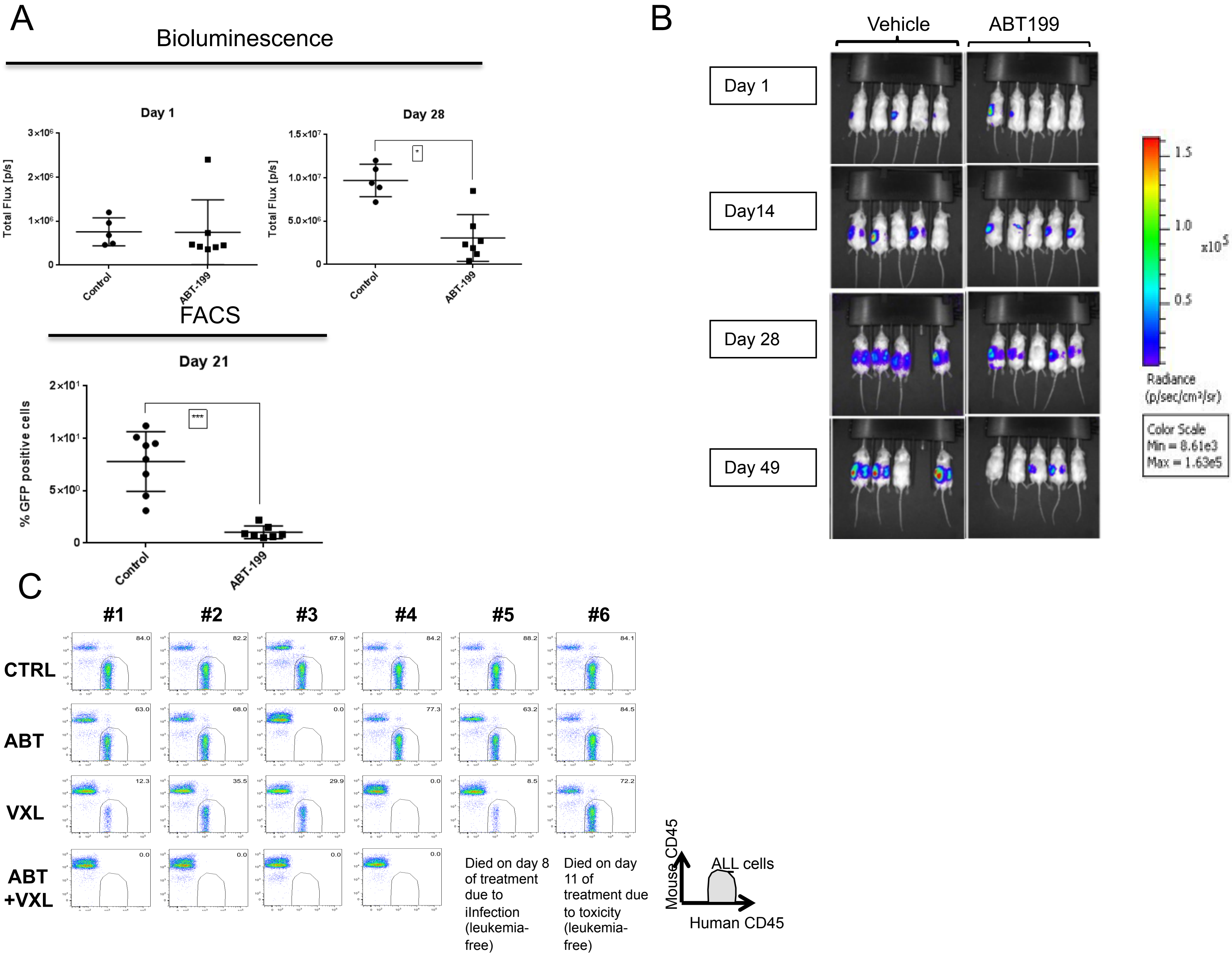

Supplemental Figure Legends.

Figure S1, Related to Figure 1. Association of high BCL-2 protein expression in MLLr with transcript levels of *BCL2. G*ene expression microarray data from 3 large cohorts of patients with ALL were analyzed. A significant increase in *BCL2* mRNA expression in MLLr samples versus normal B-cell controls was detected in the St. Jude cohort ($P = 0.015$). *BCL2* mRNA expression levels were significantly higher in the MLLr samples than E2A/PBX1 samples in all the three studies ($P = 0.0145$, 0.0005 and 0.0019) and higher than molecularly non-designated B-ALL samples in one study ($P = 0.010$).

Figure S2, Related to Figure 2. MLL/AF4 controls activation of the *BCL-2* gene. (A) ChIP-sequencing (ChIP-seq) for MLL-N (red) and AF4-C (green) in RS4;11 cells at the *BCL-2* promoter and first exon. (B) Real time PCR of MLL-AF4, wild type MLL, *HOXA9*, *RUNX1* and *BCL-2* expression in RS4;11 cells treated with either a control (black bars) or an MLL-AF4 specific siRNA (gray bars). In each case, signal was normalized to control treated cells and is the average of three independent knockdown experiments. Error bars represent the standard deviation and * indicates at least $p < 0.02$ (C) Western blots for the indicated proteins in SEM cells treated with either a control (-) or MLL-AF4 specific (+) siRNA. Four biological replicate pairs are shown. (D) ChIP experiments for MLL(N), AF4(C) and ENL in RS4;11 cells treated with either control (dark colored bars) or MLL-AF4 siRNAs (light colored bars). PCR primers are as indicated in Figure 2. Values represent the average of 2 independent knockdown experiments and error bars represent the standard error of the mean. (E) ChIP experiments for MLL(C) and AF4(N) and ENL in SEM cells treated with either control (dark colored bars) or MLL-AF4 siRNAs (light colored bars). Values represent the average of three independent knockdown experiments and error bars represent the standard error of the mean. (F) Real time PCR (left panel) and western blots (right panel) of wild type MLL and BCL-2 normalized to GAPDH in SEM cells treated with either a control (black bars) or an MLL specific siRNA (white bars). (G-I) ChIP experiments for Cyclin T1, CBX8 and DOT1L in SEM cells treated with either control (dark colored bars) or MLL-AF4 siRNAs (light colored bars). PCR primers are as indicated in Figure 2. Values represent the average of 4 (CycT1, CBX8) or 2 (DOT1L) independent knockdown experiments and error bars represent the standard error of the mean.

Figure S3, Related to Figure 3. Loss of BCL-2 reduces SEM cell growth and the DOT1L inhibitor EPZ5676 has minimal effect on the survival of Nalm-6 cells. (A) RT-PCR and western blots of SEM cells treated with BCL-2 siRNA's. (B) Colony assays of the SEM treated samples from C representing the average of two separate knockdowns. Error bars = sd. (C) Western blot analysis of SEMK2 cells treated with EPZ5676 1μM for 7 days. The numbers indicate the relative density of the bands relative to untreated control. (D) Nalm-6 cells were treated with increasing concentrations of ABT-199 and 1uM EPZ5676 and growth-inhibitory effects determined using the Cell viability luminescence assay (CellTiter-Glo®, Promega). The effective dose for 50% cell killing (IC50) was determined using Graph Pad Prism 5.0.

Figure S4, Related to Figure 5. ABT-737 or ABT-199 in ALL cell lines and primary samples. (A) Correlation between percentage (%) of mitochondrial depolarization in B-ALL cells induced by the indicated BH3 peptides and ABT-737 (Sample #18-29 in Supplementary Table 1). R values results from Spearman analysis of data (B and D). IC50 for ABT-737 (black bars) or ABT-199 (grey bars) in ALL cell lines (B) and primary samples (D). Values were determined based on the number of live cells remaining after treatment for 48 h. Among ALL cell lines, RS4;11 and SEM-K2 are t(4;11)-positive; P12-ICHIKAWA, MOLT-4, and PF382 are T-cell lines; Ramos and Raji are Burkitt cell lines. (C and E) Expression of BCL-2, BCL-X_L and MCL-1 in ALL cell lines and primary samples determined by WB analysis. Values indicate protein quantification relative to β-actin. (F) Sensitivity of pediatric B-ALL samples to ABT-199 and ABT-263. IC_{50} values of primary B-cell ALL samples is graphed following short term ex-vivo culture with ABT-199 and ABT-263 for 8hr. Values were determined based on the number of live cells remaining after treatment for 8 h. For clinical information, please refer to Table S1 (Samples #23-29).

Figure S5, related to Figure 6. FAF1 does not contribute to leukemic growth. (A) FAF1 siRNA knockdowns in SEM cells reduce FAF1 protein levels (western blot). (B) FAF1 siRNA knockdowns have no effect on SEM cell growth (colony assay).

Figure S6, related to Figure 7. ABT199 Inhibits Leukemia Progression in ALL xenograft Model In Vivo. (A) ALL-236-GFP/Luciferase cells generated from pre-B-ALL with t(4;11) were injected intravenously into the recipient NSG mice. On day 35 post-injection mice were randomized (N=5/group, Day =1) and treated with vehicle

or ABT199 (100mg/kg/day) for 10 days. Bioluminescence quantification performed at the treatment start (day 1) and on day 28 after treatment onset is shown. Leukemia burden was confirmed by detection of circulating GFP + cells on day 21. (B) BLI of NSG mice engrafted with ALL-236-GFP and treated with ABT-199 for 10 days. Time refers days relative to treatment onset. (C) Selective inhibition of BCL-2 by ABT-199 and induction type chemotherapy synergistically eradicate patient derived ALL cells in vivo. Leukemia cells from a patient with t(4;11) ALL (#682) were injected to NRG mice via tail vein. On day 24 post engraftment, mice were randomly divided into cohorts to receive VXL (Vincristine, Dexamethasone, L-asparaginase) or ABT-199 alone or in combination, or vehicle controls (n = 6/arm). Time course changes in percentages of circulating ALL cells in case #682. Leukemia progression was evaluated by determining the percentage of circulating human CD45-positive cells across different treatment groups.

Supplemental Experimental Procedures

Cell lines, primary samples, and cultures

Raji, RS4;11, Ramos, REH, NALM-6, and OP-9 cells were purchased from American Type Culture Collection (Manassas, VA). MOLT-4, P12-ICHIKAWA, and PF382 were kindly provided by Dr. Adolfo Ferrando (Columbia University, New York, NY) and SEM-K2 by Dr. Carolyn Felix (University of Pennsylvania, Philadelphia, PA). MLLr ICN3 cells were provided by Dr. Markus Muschen (UCSF, San Francisco, CA).(Duy et al., 2011) SEM cells(Greil et al., 1994) were purchased from DSMZ (www.cell-lines.de) and cultured in IMDM (Gibco) supplemented with 15% FCS. Cell lines were validated by the MD Anderson Cancer Center Cell Line Validation Core Facility. RS4;11, REH, NALM-6, and SEM-K2 represent precursor B-cell ALL, Raji and Ramos mature B-cell ALL, and MOLT-4, P12-ICHIKAWA, and PF382 T-cell ALL. RS4;11, SEM and SEM-K2 carry t(4;11), SEM-K2 is a subclone of SEM with identical features.(Zweidler-McKay et al., 2005) Except for SEM, cell lines were maintained in RPMI 1640 medium containing 10% heat-inactivated FBS. For treatment studies, REH, SEMK2 and RS4;11 were treated with ABT-737 or ABT-199 in combination with vincristine (VCR), doxorubicin (DOX), cytarabine (AraC), dexamethasone (DEXA), or L-asparaginase (L-ASP) for 48 h (REH, SEMK2) or 24 h (RS4;11). For Figure 3H and S3C SEMK2 cells were treated with ABT-199 in combination with DOT1L inhibitors SGC0946 (Sigma) or EPZ5676 (BioVision, Inc.) for 4 or 7 days, and effects on cell viability determined using the Cell viability luminescence assay (CellTiter-Glo®, Promega). The effective dose for 50% cell killing (IC50) was determined using Graph Pad Prism 5.0. For treatment of primary samples, patient mononuclear cells were plated on a monolayer of OP-9 stromal cells in RPMI 1640 medium containing 10% FBS and supplemented with the following recombinant human cytokines (all from Gemini Bio-Products): TPO, FLT3 ligand, and IL-3 (10 ng/mL); IL-6 (20 ng/mL); and SCF (100 ng/mL). Cells were treated with ABT-737, ABT-199, a chemotherapy agent, or a combination of ABT-199 and a chemotherapy agent for 24 h. For assessment of pediatric and adult ALL samples from Dana-Farber Cancer hospital primary patient mononuclear cells were grown in RPMI 1640 with 10% FBS and supplemented with IL-3 (10 ng/mL); IL-6 (20 ng/mL); and SCF (100 ng/mL). Cells were treated with ABT-199 and ABT-263 for 8hr prior to measuring cell viability.

Cell viability, apoptosis analysis and BH3 profiling

Viable cells were enumerated by flow cytometry using counting beads (Invitrogen, Carlsbad, CA) with concurrent Annexin-V and DAPI or 7-aminoactinomycin D (7-AAD) staining. IC_{50} values for ABT-199 or ABT-737 were calculated from numbers of live (Annexin V-negative/DAPI or 7AAD-negative) cells after treatment (at 48 h for cell lines or 24 h for primary samples) by using Calcusyn software (Biosoft, Ferguson, MO). Calcusyn was utilized to determine combination index values based on the percentage of cell death induced by each treatment.

For intracellular BH3 profiling of primary ALL cells, thawed primary ALL cells were washed once with PBS and stained with 1:100 Invitrogen Live/Dead – Aqua stain (#34957, Life Technologies) in FACS buffer (2% FBS in PBS, 1:100) for 20 min on ice, washed with FACS buffer, and subsequently stained with CD45-V450 (#642275; BD Biosciences; 1:100) in FACS buffer on ice for 20 min. ALL primary cells were identified as CD45 mid, SSC-A low. Intracellular BH3 profiling was performed as described in Pan et al (Pan et al., 2014).

siRNA experiments

Briefly, using a rectangle pulse EPI 2500 electroporator (Fischer, Heidelberg), 7 X 10^7 SEM or RS4;11 cells were subjected to a 10msec 350V (SEM) or 370V (RS4;11) electroporation in the presence of 300 pmol siRNA. MLL-AF4 siRNA sequences were obtained from(Thomas et al., 2005) and are the following: siMA6 (sense, AAGAAAAGCAGACCUACUCCA;antisense, UGGAGUAGGUCUGCUUUUCUUUU), targeting the MLL exon 9 and AF4 exon 4 MLL-AF4 fusion site present in SEM cells, and siMARS (sense, ACUUUAAGCAGACCUACUCCA-; antisense, UGGAGUAGGUCUGCUUAAAGUCC-), targeting the exon 10–exon 4 fusion site variant present in RS4;11 cells. As control siRNAs we used the mismatch control siMM (sense, AAAAGCUGACCUUCUCCAAUG; antisense, CAUUGGAGAAGGUCAGCUUUUCU). Wild type MLL siRNAs (Dharmacon on Target Plus Smartpool, L-009914-00), FAF1 siRNAs (Dharmacon on Target Plus Smartpool, L-009106-00) and BCL-2 siRNAs (Dharmacon on Target Plus Smartpool, L-003307-00) were all compared to a non-targeting smartpool control (Dharmacon On Target plus non targeting pool D001801020).

Colony forming assays

24 hours post second transfection cells were plated at a density of 1, 2 or 2.5 x 10^5 cells per ml, in triplicate, plated in IMDM MethoCult media (H4100; STEMCELL Technologies) supplemented with FCS and cultured for 14 days (37 °C, 5% CO_2) before counting. Colony forming assays were run in triplicate with at two-three biological repeats.

Western blot analysis

The following mAb were used: mouse anti-BCL-2 (Dako Cytomation, Carpinteria, CA); rabbit anti-BCL-X_L, BAX, BIM, FAF1 (Cell Signaling Technology, Beverly, MA); mouse MCL-1 (BD Pharmingen, San Diego, CA); and mouse anti-β-actin (Sigma-Aldrich). An Odyssey Infrared Imaging System and Odyssey software v2.0 (LI-COR Biosciences, Lincoln, NE) were used to scan the blots and quantify band intensities, respectively. The ratio of band intensity of each BCL-2 family protein relative to that of loading control was normalized to the ratio in untreated NALM-6 cells. For Figures 2, 3, 4, S2 and S5 the following antibodies were used for western blotting: a-AF4-C (For both MLL/AF4 and wild type AF4, Abcam, ab31812); a-MLL-C (Active Motif, 61295); a-ENL (Bethyl, A302-268A); a-BCL-2 (Cell Signaling, 2870); a- BCL-X_L (Cell Signaling, 2764); a-MCL-1 (Cell Signaling, 5453); a-BIM (Abcam, ab32158); a-BAX (Cell Signaling,2772); a-GAPDH (Bethyl, A300-641A); a-H3 (Abcam, ab1791); a-RUNX1 (Cell Signaling, 4334); a-H3K79me2 (Active Motif 39143); a-H3K79me3 (Diagenode pAb-068-050), a-FAF1 (Bethyl, A302-810A).

Bimolecular fluorescence complementation

The coding sequences for human BCL-2, BCL-X_L, MCL-1, BIM, and NOXA were subcloned by standard PCR strategies into BiFC plasmids containing Venus fragments (FLAG/VN173 or HA/VC155; VN and VC are the N- and C-terminal fragments of the Venus protein, respectively). HeLa cells were grown to at least 50% of confluence in 24-well plates. One hour before transfection, 2.5 µM of ABT-199 or vehicle (DMSO) was added. Cells then were transfected with a pBiFC vector (0.3 µg/10^5 cells) containing the cDNA for a VN-BCL-2/BCL-X_L/MCL-1 fusion and a pBiFC vector expressing a VC-BIM fusion. Lipofectamine 2000 (Invitrogen) was used for transfection according to the manufacturer's instructions. Cells were co-transfected with an equal amount of the pAL2-mRFP vector containing *mRFP* cDNA to assess transfection efficiency and relative fluorescence intensities. Z-VAD-fmk (50 µM) was added to delay cell death and preserve cell morphology. After transfection, cells were cultured for 24 h and subjected to trypsinization; Venus and mRFP signals were quantified in a FACSCalibur flow cytometer using 488-nm and 635-nm excitation lasers, respectively. A gating analysis based on mRFP fluorescence was performed to exclude non-transfected cells. The mean fluorescence intensities of the BiFC complexes were normalized to the mean fluorescence intensity of mRFP. At least 10,000 cells were analyzed in each experiment. Results are expressed as the fold-change induced by ABT-737 or ABT-199 in the Venus/RFP intensity ratio for each protein pair. Results are the mean ± SD of two independent experiments, with duplicates (n=4).

Chromatin immunoprecipitation assays

For RS4;11 ChIP-seq, fixed chromatin samples were fragmented by a Bioruptor sonicator (Diagenode, Denville, NJ) for 20 min at high in a constantly circulating 4°C water bath to an average size of 200-500 bp. For all other ChIP and ChIP-seq, samples of up to 10^8 cells were sonicated on a Covaris (Woburn, MA) according to the manufacturers' recommendations. Ab:chromatin complexes were collected with a mixture of Protein A and Protein G Dynabeads (Life Technologies, Grand Island, NY) by using a magnet and were then washed twice with a solution of 50mM Hepes-KOH, pH 7.6, 500mM LiCl, 1mM EDTA, 1% NP-40, and 0.7% Na-deoxycholate. After a Tris-EDTA wash, samples were eluted, treated with RNAse and proteinase K, and purified by using a Qiagen PCR purification kit. ChIP samples were quantified relative to inputs (Milne et al., 2009). Briefly, the amount of genomic DNA co-precipitated with antibody was calculated as a percentage of total input using the following formula: $\Delta C_T = C_T$ (input) − C_T (ChIP), total percentage = $2^{\Delta CT} \times 5.0\%$. A 50-µL aliquot taken from each of 1 mL of sonicated, diluted chromatin before Ab incubation served as the input, and thus the signal from the input samples represents 5% of the total chromatin used in each ChIP. Histone modification % input ChIP signal was normalized to H3 % input ChIP signal. C_T values were determined by choosing threshold values in the linear range of each PCR reaction.

ChIP sequencing.

ChIP samples were submitted to the Wellcome Trust Centre for Human Genetics for library preparation(Lamble et al., 2013) and sequencing. Samples were sequenced using a HiSeq 2500 and 50bp paired-end sequencing. Data were mapped to the Homo sapiens hg18 genome using Bowtie. Conversion to bam files was done using samtools. Duplicate reads were removed and data was normalized to an input track, in SeqMonk. Peaks were called using the probe generator in SeqMonk. The data discussed in this publication have been deposited in NCBI's Gene Expression Omnibus (Edgar et al., 2002) and are accessible through GEO Series accession number GSE 74812.

(http://www.ncbi.nlm.nih.gov/geo/query/acc.cgi?acc=GSE74812).

Antibodies used for ChIP and ChIP-seq assays

a-MLL-N (Bethyl, A300-086A, ChIP and ChIP-seq); a-AF4-C (Abcam, ab31812, ChIP, ChIP-seq and western blot); a-AF4-N (Bethyl, A302-344A, ChIP); a-ENL (Bethyl, A302-268A, ChIP and ChIP-seq); a-AF9 (Bethyl, A300-595A, ChIP); a-CDK9 (Bethyl, A303-493A, ChIP); a-CyclinT1 (Bethyl, A303-496A, ChIP); a-AFF4 (Bethyl, A302-538A, ChIP); a-MLL-C (Active Motif, 61295, ChIP); a-CFP1 (Bethyl A303-161A); a-DOT1L (Bethyl, A300-953A, ChIP); a-H3K79me2 (Millipore 04-835, ChIP-seq; Active Motif 39143 ChIP) a-H3K79me3 (Diagenode pAb-068-050, ChIP and ChIP-seq) ; a-H3 (Abcam, ab1791); a-H3K4me3 (Diagenode, C1541003 ChIP and ChIP-seq); a-H3K27Ac (Diagenode, C15410196, ChIP and ChIP-seq); a-H3K4me1 (Diagenode, C15410194, ChIP-seq); a-CBX8 (Bethyl, A300-882A).

PCR primers for RT-PCR

The following Taqman primer-probe sets were purchased from Applied Biosytems (Waltham, MA, USA): b2M Hs99999007; GAPDH Hs03929097_g1; BCL-2 Hs00608023_m1; BAX Hs00180269_m1; MCL-1 Hs01050896_m1; BCL2L1 Hs00236329_m1; BCL2L11 Hs00708019; MLL Hs00172962_m1; ENL Hs00172962_m1; RUNX1 Hs00231079_m1. HOXA9 Taqman primer/probe set were as follows: HOXA9primer For: AAAACAATGCCGAGAATGAGAGCG, HOXA9 FAM/TAMRA probe: CCCCATCGATCCCAATAACCCAGC, HOXA9primer Rev: TGGTGTTTTGTATAGGGGGACC; MLL/AF4 SYBR green primers for both SEM and RS4;11 cells are as follows: MLL/AF4 For: AGGTCCAGAGCAGAGCAAAC; MLL/AF4 Rev: CGGCCATGAATGGGTCATTTC, GAPDH SYBR green primers are as follows: For: AACAGCGACACCCATCCTC; Rev CATACCAGGAAATGAGCTTGACAA.

PCR primers for ChIP

Negative control region For: GGCTCCTGTAACCAACCACTACC, Negative control region Rev: CCTCTGGGCTGGCTTCATTC; HOXA9 For: ATGCTTGTGGTTCTCCTCCAGTTG, HOXA9 Rev: CCGCCGCTCTCATTCTCAGC; HOXC8 For: AGACTTCTTCCACCACGGCAC, HOXC8 Rev: TAAGCGAGCACGGGTTCTGC; BCL2-1For: GAGGAGGGCTCTTTCTTTCTTC, BCL2-1Rev: GCCTGTCCTCTTACTTCATTCTC; BCL2-2For: CGATAACGCCTGCCATCTAA, BCL2-2Rev: CCACCACATCCTACTGGATTAC; BCL-2-3 primers are from,(Dawson et al., 2011) BCL2-3For: AGCCCCTGGAGAAGTATGGT, BCL2-3Rev: CATCCGTTAGCATGAAGCAA; BCL2-4For: GGCCAGGGTCAGAGTTAAATAG, BCL2-4Rev: GGAGGTTCTCAGATGTTCTTCTC; BCL2-5For: GGAAACCCAGACCAACTCAT, BCL2-5Rev: CCTTATCTCAGGAGGACGTAGA; BCL2-6For: GAGCCCTCAACCTTGTGATAG, BCL2-6Rev: AAGGTAGCCCTGACCATAGA; BAX-10For: CGTGGGCTATATTGCTAGATCC, BAX-10Rev: CTTCCAGGCAGGACGTTATAG; BAX-11For: TTTGCACTTGCTAATTCCTTCTG, BAX-11Rev: GCAGCTCTAATGCCTTCATTTATC; BIM-8For: CTCTTGGCAGAGACAGAAAGG, BIM-8Rev: CAGAGAACGCAGTGTGAGAA; BIM-9For: GAGGAAGTTGTTGGAGGAGAATAG, BIM-9Rev: CTCGCCACTTGTCCTTGTT; MCL1-4For: CCAAACATTGGACTGAGAGTAGAG, MCL1-4Rev: GTTCAGTGATGGATGGGAACA; MCL1-5For: TTCCGCCCATCTTGATTCTT, MCL1-5Rev: TCGCTACTGGGATTTACAGAAC; MCL1-6For: CCTGAGTTCTGTAAATCCCAGTAG, MCL1-6Rev: CGAAGCATGCCTGAGAAAGA; MCL1-7For: GGCATCTTTGGATTTCAGTCTTG, MCL1-7Rev: CTGTAGAGGGAGCAGAACAATC; BCLXL-12For: GGAAGGCATTTCGGAGAAGA, BCLXL-12Rev: TCTGGGTCTAGGTTCCAAGATA; BCLXL-13For: GAGCTGGTGGTTGACTTTCT, BCLXL-13Rev: CAGTCCTGTTCTCTTCCACATC; BCLXL-14For: GCTTCAGAGATCAGGCATCTT, BCLXL-14Rev: CCTCACAGGTTTGGGACTTAAT; BCLXL-15For: CAGTGTAGGCTGTGCAGATT, BCLXL-15Rev: GCAAGTGCTCCACAAACAAG.

Patient microarray gene expression data

Microarray gene expression data from three large cohorts of patients with ALL were analyzed. These cohorts included the Eastern Cooperative Oncology Group (ECOG) Clinical Trial E2993 (GEO#: GSE34861) cohort: 191 total samples comprising 78 BCR-ABL1 patients, 6 E2A-PBX1 patients, 25 MLLr patients (t(4;11): 17, other MLLr: 8), and 82 other B-ALL patients;(Geng et al., 2012) the Children's Oncology Group (COG) Clinical Trial P9906 (GEO#: GSE28460) cohort: 207 total samples, 23 E2A-PBX1 patients, 21 MLLr patients, 3 RUNX1-ETV6 patients, 155 other B-ALL patients (trisomy 4 or 10 patients);(Harvey et al., 2010) and the St. Jude Research Hospital pediatric ALL clinical trial cohort: 132 total samples, 15 BCR-ABL1 patients, 18 E2A-PBX1 patients, 20 MLLr patients, 20 RUNX1-ETV6 patients, 17 hyperdiploid patients, 28 other B-ALL patients, 14 T-ALL patients

(Ross et al., 2003). This last cohort has no GEO number, but raw data can be downloaded from the following site: http://www.stjuderesearch.org/site/data/ALL3/.

The microarray raw data were normalized by using the Robust Multi-array Average (RMA) method(Bolstad et al., 2003) with Expression Console software (Version 1.1, Affymetrix) for the Affymetrix arrays HG-U133 plus2 (COG data) or HG-U133 A and B (St. Jude data), or with NimbleScan software (version 2.5, Roche NimbleGen, Madison, WI) for the NimbleGen array HG18 60mer expression 385K platform (ECOG data). The patients in each cohort were grouped into subtypes according to their cytogenetic features: BCR-ABL, E2A-PBX1, MLLr, ETV6-RUNX1, or other ALLs, which were negative for these translocations. T-ALL samples were excluded from this analysis. MLL fusion partner information was available for the ECOG MLLr ALL data, which were therefore further separated into MLL/AF4 (n=17) or other MLLr (n=8). No MLL fusion partner information was available for the COG or St. Jude clinical trials, so MLLr ALL patients were treated as one group. Expression level of a gene in a sample was determined by the average of expression values from multiple probe sets on the array representing this gene. The *P*-values of differential expression of *BCL2* between MLLr and other ALL subtypes were determined by two-sided Wilcoxon test. All downstream microarray analysis was performed by using R version 2.14.0 (R Development Core Team. R: A Language and Environment for Statistical Computing. 2009; http://www.R-project.org).

RPPA statistical analysis
Expression of pro- and anti-apoptotic BCL-2 family proteins (BAX, BCL-2, MCL-1, BCL-X_L, and BIM) was studied in 186 newly diagnosed ALL. Associations between RPPA protein expression levels and categorical clinical variables were assessed in R using a standard *t*-test, linear regression, or mixed-effects linear model. Associations between continuous variable and protein levels were assessed by using Pearson and Spearman correlations and linear regression. Bonferroni corrections were performed to account for multiple statistical parameters for calculating statistical significance. RPPA antibody information is provided in Table S4.

***In vivo* murine leukemia models**
Three *in vivo* murine leukemia models were used. In ONE model, 10^6 ALL-236-GFP/LUC cells generated from pre-B-ALL with t(4;11) as described elsewhere (Terziyska et al., 2012) were injected intravenously into NOD SCID/IL2Rγ-KO (NSG) mice. On day 21 after injection, peripheral blood samples were collected by retro-orbital bleeding and the presence of GFP-positive cells determined by FACS. Engraftment was detected on day 32 after cell injection by bioluminescence imaging (BLI; IVIS-200, Xenogen, Cranbury, NJ) following administration of the LUC substrate coelenterazine (native; Biotium, Heyward, CA). Three days later, the mice began daily treatment with vehicle (Phosal 50PG/PEG 40/ethanol, 60/30/10 v/v) or ABT-199 (100 mg/kg per day) by oral gavage for 10 days (5 days/week with a drug-free weekend holiday). Antitumor activity was tested by BLI 2 and 4 weeks after initiation of treatment.

For the second model, 12 NSG mice were injected intravenously with ICN3 xenograft cells (2.5×10^5 cells/mouse) generated from a child with relapsed MLLr pre-B-ALL (Duy et al., 2011). Leukemia engraftment was established by detection of circulating human CD19-positive cells in peripheral blood. At day 45 after injection, mice were randomized into two treatment groups (n=6/group) for treatment with vehicle only or ABT-199 (100 mg/kg per day). Mice were treated by oral gavage for 5 consecutive days, given a drug-free weekend holiday and then were treated for 2 more days. Peripheral blood was collected on days 4 and 10 after treatment initiation. Mice were euthanized the morning after the last drug dose. BM, spleen, and peripheral blood were analyzed for leukemia burden by CD19 cytometry.

For the third model, we combined ABT-199 and an induction-type regimen consisting of VCR, L-ASP, and DEXA (VXL).(Szymanska et al., 2012) NOD.Cg-Rag1^{tm1Mom} IL2rg^{tm1Wjl}/SzJ (NRG) mice were obtained from Jackson Laboratory (Bar Harbor, ME) and were bred by standard procedures in accordance with a protocol approved by the IACUC. NRG mice were preconditioned with a single i.p. dose of busulfan (40 mg/kg) 24 h prior to tail vein injection of cryopreserved human patient xenograft pre-B-ALL cells with t(4;11), precoated with OKT3 Ab, as described previously.(Wunderlich et al., 2014) After engraftment of human CD45-positive ALL cells to peripheral blood was observed, mice were randomly divided into cohorts to receive treatment with VCR (0.15 mg/kg in PBS, i.p., 2 doses, days 1 and 8), DEXA (5 mg/kg in PBS, i.p., 7 doses, days 1-5, 8-9), and L-ASP (1000 IU/kg in PBS, i.p., 7 doses, days 1-5, 8-9); ABT-199 (100 mg/kg in 10% ethanol/30% PEG400/60% Phosal50 PG, by oral gavage,

8 doses, days 1-5, 8-9, 11); a combination of the two; or vehicle alone (controls). B-ALL grafts were measured as the percentage of human CD45+CD19+CD33- cells in the peripheral blood by flow cytometry.

Supplemental References

Bolstad, B.M., Irizarry, R.A., Astrand, M., and Speed, T.P. (2003). A comparison of normalization methods for high density oligonucleotide array data based on variance and bias. Bioinformatics *19*, 185-193.

Dawson, M.A., Prinjha, R.K., Dittmann, A., Giotopoulos, G., Bantscheff, M., Chan, W.I., Robson, S.C., Chung, C.W., Hopf, C., Savitski, M.M.*, et al.* (2011). Inhibition of BET recruitment to chromatin as an effective treatment for MLL-fusion leukaemia. Nature *478*, 529-533.

Duy, C., Hurtz, C., Shojaee, S., Cerchietti, L., Geng, H., Swaminathan, S., Klemm, L., Kweon, S.M., Nahar, R., Braig, M.*, et al.* (2011). BCL6 enables Ph+ acute lymphoblastic leukaemia cells to survive BCR-ABL1 kinase inhibition. Nature *473*, 384-388.

Edgar, R., Domrachev, M., and Lash, A.E. (2002). Gene Expression Omnibus: NCBI gene expression and hybridization array data repository. Nucleic Acids Res *30*, 207-210.

Geng, H., Brennan, S., Milne, T.A., Chen, W.Y., Li, Y., Hurtz, C., Kweon, S.M., Zickl, L., Shojaee, S., Neuberg, D.*, et al.* (2012). Integrative epigenomic analysis identifies biomarkers and therapeutic targets in adult B-acute lymphoblastic leukemia. Cancer Discov *2*, 1004-1023.

Greil, J., Gramatzki, M., Burger, R., Marschalek, R., Peltner, M., Trautmann, U., Hansen-Hagge, T.E., Bartram, C.R., Fey, G.H., Stehr, K.*, et al.* (1994). The acute lymphoblastic leukaemia cell line SEM with t(4;11) chromosomal rearrangement is biphenotypic and responsive to interleukin-7. Br J Haematol *86*, 275-283.

Harvey, R.C., Mullighan, C.G., Wang, X., Dobbin, K.K., Davidson, G.S., Bedrick, E.J., Chen, I.M., Atlas, S.R., Kang, H., Ar, K.*, et al.* (2010). Identification of novel cluster groups in pediatric high-risk B-precursor acute lymphoblastic leukemia with gene expression profiling: correlation with genome-wide DNA copy number alterations, clinical characteristics, and outcome. Blood *116*, 4874-4884.

Lamble, S., Batty, E., Attar, M., Buck, D., Bowden, R., Lunter, G., Crook, D., El-Fahmawi, B., and Piazza, P. (2013). Improved workflows for high throughput library preparation using the transposome-based Nextera system. BMC biotechnology *13*, 104.

Milne, T.A., Zhao, K., and Hess, J.L. (2009). Chromatin immunoprecipitation (ChIP) for analysis of histone modifications and chromatin-associated proteins. Methods Mol Biol *538*, 409-423.

Pan, R., Hogdal, L.J., Benito, J.M., Bucci, D., Han, L., Borthakur, G., Cortes, J., DeAngelo, D.J., Debose, L., Mu, H.*, et al.* (2014). Selective BCL-2 inhibition by ABT-199 causes on-target cell death in acute myeloid leukemia. Cancer Discov *4*, 362-375.

Ross, M.E., Zhou, X., Song, G., Shurtleff, S.A., Girtman, K., Williams, W.K., Liu, H.C., Mahfouz, R., Raimondi, S.C., Lenny, N.*, et al.* (2003). Classification of pediatric acute lymphoblastic leukemia by gene expression profiling. Blood *102*, 2951-2959.

Szymanska, B., Wilczynska-Kalak, U., Kang, M.H., Liem, N.L., Carol, H., Boehm, I., Groepper, D., Reynolds, C.P., Stewart, C.F., and Lock, R.B. (2012). Pharmacokinetic modeling of an induction regimen for in vivo combined testing of novel drugs against pediatric acute lymphoblastic leukemia xenografts. PLoS One *7*, e33894.

Terziyska, N., Castro Alves, C., Groiss, V., Schneider, K., Farkasova, K., Ogris, M., Wagner, E., Ehrhardt, H., Brentjens, R.J., zur Stadt, U.*, et al.* (2012). In vivo imaging enables high resolution preclinical trials on patients' leukemia cells growing in mice. PLoS One *7*, e52798.

Thomas, M., Gessner, A., Vornlocher, H.P., Hadwiger, P., Greil, J., and Heidenreich, O. (2005). Targeting MLL-AF4 with short interfering RNAs inhibits clonogenicity and engraftment of t(4;11)-positive human leukemic cells. Blood *106*, 3559-3566.

Wunderlich, M., Brooks, R.A., Panchal, R., Rhyasen, G.W., Danet-Desnoyers, G., and Mulloy, J.C. (2014). OKT3 prevents xenogeneic GVHD and allows reliable xenograft initiation from unfractionated human hematopoietic tissues. Blood *123*, e134-144.

Zweidler-McKay, P.A., He, Y., Xu, L., Rodriguez, C.G., Karnell, F.G., Carpenter, A.C., Aster, J.C., Allman, D., and Pear, W.S. (2005). Notch signaling is a potent inducer of growth arrest and apoptosis in a wide range of B-cell malignancies. Blood *106*, 3898-3906.
