# Supplementary material for: MLL-Rearranged Acute Lymphoblastic Leukemias Activate BCL-2 through H3K79 Methylation and Are Sensitive to the BCL-2-Specific Antagonist ABT-199
Source: Cell Rep. 2015 Dec 17;13(12):2715–27. doi: 10.1016/j.celrep.2015.12.003 (PMC4700051; doi:10.1016/j.celrep.2015.12.003)
Supplement: Document S2. Article plus Supplemental Information [file mmc2.pdf]

Cell Reports

Article

MLL-Rearranged Acute Lymphoblastic Leukemias Activate BCL-2 through H3K79 Methylation and Are Sensitive to the BCL-2-Specific Antagonist ABT-199

Graphical Abstract

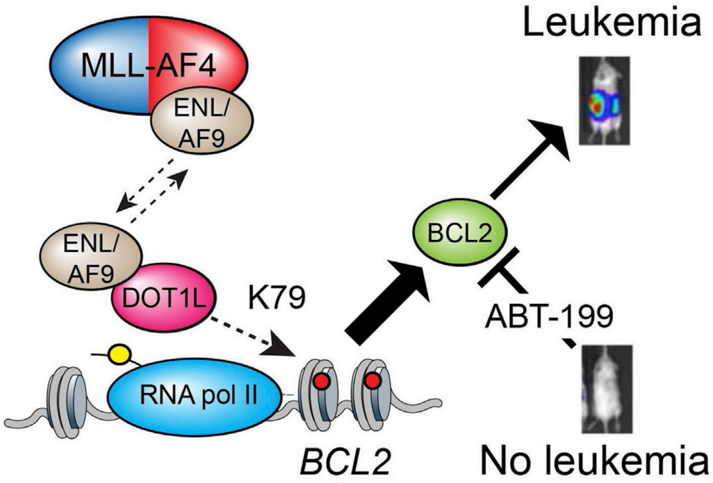

Highlights

- *MLLr* ALL blasts express high levels of BCL-2, BAX, and BIM
- MLL/AF4 activates *BCL2* through H3K79 methylation
- *MLLr* ALL cells are exquisitely sensitive to BCL-2 antagonist ABT-199
- ABT-199 treatment synergizes with H3K79 methylation inhibitors on *MLLr* samples

Authors

Juliana M. Benito, Laura Godfrey, Kensuke Kojima, ..., Anthony Letai, Thomas A. Milne, Marina Konopleva

Correspondence

thomas.milne@imm.ox.ac.uk (T.A.M.), mkonople@mdanderson.org (M.K.)

In Brief

Therapies designed to exploit specific molecular pathways in aggressive cancers are an exciting area of research. Mutations in the MLL gene cause aggressive incurable leukemias. Benito et al. show that MLL leukemias are highly sensitive to BCL-2 inhibitors, especially when combined with drugs that target mutant MLL complex activity.

Accession Numbers

GSE74812

Benito et al., 2015, Cell Reports *13*, 2715–2727
December 29, 2015 ©2015 The Authors
http://dx.doi.org/10.1016/j.celrep.2015.12.003

Cell Reports
Article

OPEN ACCESS
CellPress

MLL-Rearranged Acute Lymphoblastic Leukemias Activate BCL-2 through H3K79 Methylation and Are Sensitive to the BCL-2-Specific Antagonist ABT-199

Juliana M. Benito,[1] Laura Godfrey,[2] Kensuke Kojima,[3] Leah Hogdal,[4] Mark Wunderlich,[5] Huimin Geng,[6] Isabel Marzo,[7] Karine G. Harutyunyan,[1] Leonard Golfman,[8] Phillip North,[2] Jon Kerry,[2] Erica Ballabio,[2] Triona Ní Chonghaile,[9] Oscar Gonzalo,[7] Yihua Qiu,[1] Irmela Jeremias,[10] LaKiesha Debose,[1] Eric O'Brien,[5] Helen Ma,[1] Ping Zhou,[1] Rodrigo Jacamo,[1] Eugene Park,[6] Kevin R. Coombes,[11] Nianxiang Zhang,[11] Deborah A. Thomas,[1] Susan O'Brien,[1] Hagop M. Kantarjian,[1] Joel D. Leverson,[12] Steven M. Kornblau,[1] Michael Andreeff,[1] Markus Müschen,[6] Patrick A. Zweidler-McKay,[8] James C. Mulloy,[5] Anthony Letai,[4] Thomas A. Milne,[2,*] and Marina Konopleva[1,*]
[1]Department of Leukemia, The University of Texas MD Anderson Cancer Center, Houston, TX 77030, USA
[2]Weatherall Institute of Molecular Medicine, Molecular Haematology Unit, NIHR Oxford Biomedical Research Centre Programme, University of Oxford, Headington, Oxford OX3 9DS, UK
[3]Division of Hematology, Respiratory Medicine and Oncology, Department of Internal Medicine, Faculty of Medicine, Saga University, Saga 840-8502, Japan
[4]Department of Medical Oncology, Dana-Farber Cancer Institute, Boston, MA 02215, USA
[5]Cancer and Blood Diseases Institute, Cincinnati Children's Hospital Medical Center, Cincinnati, OH 45229, USA
[6]Department of Laboratory Medicine, University of California, San Francisco, San Francisco, CA 94143, USA
[7]Department of Biochemistry, Molecular and Cell Biology, University of Zaragoza, 50018 Zaragoza, Spain
[8]Division of Pediatrics, The University of Texas MD Anderson Cancer Center, Houston, TX 77030, USA
[9]Department of Physiology and Medical Physics, Royal College of Surgeons in Ireland, York House, Dublin 2, Ireland
[10]German Research Center for Environmental Health (GmbH), 85764 Neuherberg, Germany
[11]Department of Bioinformatics and Computational Biology, The University of Texas MD Anderson Cancer Center, Houston, TX 77030, USA
[12]Department of Oncology Development, AbbVie Inc., North Chicago, IL 60064, USA
*Correspondence: thomas.milne@imm.ox.ac.uk (T.A.M.), mkonople@mdanderson.org (M.K.)
http://dx.doi.org/10.1016/j.celrep.2015.12.003
This is an open access article under the CC BY license (http://creativecommons.org/licenses/by/4.0/).

SUMMARY

Targeted therapies designed to exploit specific molecular pathways in aggressive cancers are an exciting area of current research. *Mixed Lineage Leukemia* (*MLL*) mutations such as the t(4;11) translocation cause aggressive leukemias that are refractory to conventional treatment. The t(4;11) translocation produces an MLL/AF4 fusion protein that activates key target genes through both epigenetic and transcriptional elongation mechanisms. In this study, we show that t(4;11) patient cells express high levels of BCL-2 and are highly sensitive to treatment with the BCL-2-specific BH3 mimetic ABT-199. We demonstrate that MLL/AF4 specifically upregulates the *BCL-2* gene but not other BCL-2 family members via DOT1L-mediated H3K79me2/3. We use this information to show that a t(4;11) cell line is sensitive to a combination of ABT-199 and DOT1L inhibitors. In addition, ABT-199 synergizes with standard induction-type therapy in a xenotransplant model, advocating for the introduction of ABT-199 into therapeutic regimens for MLL-rearranged leukemias.

INTRODUCTION

Mixed-lineage-leukemia (*MLL*) is one of the most frequently translocated genes (*MLL*-rearranged or *MLLr*) in hematologic malignancies and produces aggressive leukemias where more targeted therapeutic approaches are particularly needed. Translocation t(4;11)(q21;q23) generates *MLL/AF4* and *AF4/MLL* fusion products, both of which function as transcriptional activators. The role of AF4/MLL in t(4;11) leukemias is controversial, as it has transformation potential (Bursen et al., 2010) but is not expressed in all t(4;11) patients (Andersson et al., 2015). Conversely, the MLL/AF4 fusion protein is expressed in all t(4;11) patients, and knockdowns of MLL/AF4, even in the presence of AF4/MLL, are sufficient to stop t(4;11) leukemias from growing (Thomas et al., 2005).

t(4;11) leukemias are diagnosed mainly as precursor B cell acute lymphoblastic leukemia (B-ALL) in both infants, children, and adults, and they predict poor long-term outcomes, even with aggressive chemotherapy or therapy combined with stem cell transplantation (Beldjord et al., 2014; Dreyer et al., 2015; Pieters et al., 2007). t(4;11) leukemias have very few cooperating mutations, especially in infants (Andersson et al., 2015), suggesting that MLL/AF4 is the primary driver of continued leukemogenesis. Therefore, understanding the function of the MLL/AF4 fusion protein and the genes that it regulates will be essential for the development of targeted t(4;11) therapies.

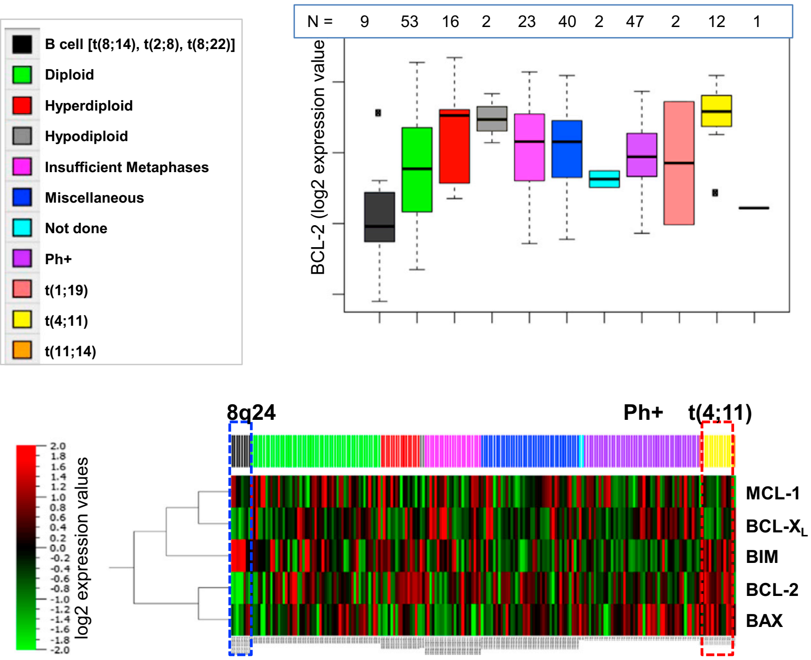

Figure 1. RPPA Profiling of BCL-2 Proteins in ALL, Showing Heatmaps of Differentially Expressed Proteins Based on Cytogenetics

Black bars indicate 8q24 leukemia samples and their expression patterns are shown in the blue dashed line box. Yellow bars indicate t(4;11)-positive samples, and their expression patterns are shown in the red dashed line box.
See also Figure S1.

BCL-2 family proteins mediate an intrinsic, mitochondrial apoptosis pathway. BCL-2, BCL-X_L, and MCL-1 are anti-apoptotic BCL-2 family proteins, while BCL-2 homology 3 (BH3) proteins BIM, BID, BAD, NOXA, PUMA, and HRK are pro-apoptotic proteins that trigger cell death. Previous studies demonstrated high expression of *BCL-2* in *MLLr* pediatric ALL (Robinson et al., 2008). Using chromatin immunoprecipitation sequencing (ChIP-seq), we and others have detected direct binding of MLL/AF4 (Guenther et al., 2008; Wilkinson et al., 2013) to the *BCL-2* gene. This suggests, but does not completely establish, that MLL/AF4 and other fusion proteins could be the cause of increased BCL-2 levels through direct upregulation of *BCL-2* transcription. Supporting the potential importance of this observation, activity of the first-generation BCL-2 antagonists has indicated that BCL-2 inhibition could be exploited for *MLLr* leukemias (Robinson et al., 2008; Urtishak et al., 2013). ABT-199/GDC-0199 (venetoclax) is a BH3 mimetic that specifically targets BCL-2 while sparing BCL-X_L, thus avoiding thrombocytopenia (Chonghaile et al., 2014; Pan et al., 2014; Souers et al., 2013; Vaillant et al., 2013; Vandenberg and Cory, 2013). ABT-199 has achieved promising anti-leukemia activity in patients with chronic lymphocytic leukemia (CLL) (Molica, 2015), and it has been reported to have preclinical activities in estrogen-receptor-positive breast cancer, acute myeloid leukemia (AML), early T cell progenitor leukemia, Myc-driven B cell lymphomas, and acute lymphoblastic leukemia (Alford et al., 2015; Chonghaile et al., 2014; Pan et al., 2014; Souers et al., 2013; Vaillant et al., 2013; Vandenberg and Cory, 2013).

Recruitment of P-TEFb (a heterodimer consisting of Cyclin T1 or T2 and the CDK9 kinase) and transcription elongation factors such as ENL and AF9 (Lin et al., 2010; Mueller et al., 2007; Yokoyama et al., 2010) are thought to be major ways in which MLL/AF4 activates gene targets. Other mechanisms have been proposed, including an ENL/AF9 direct interaction with the polycomb group (PcG) protein CBX8 (Maethner et al., 2013). In addition, ENL and AF9 interact directly with DOT1L (Biswas et al., 2011; Leach et al., 2013; Mohan et al., 2010), a histone methyltransferase that specifically methylates lysine 79 on histone 3. Since ENL or AF9 and DOT1L exist in a separate, distinct complex from MLL/AF4 (Biswas et al., 2011; Leach et al., 2013), it is unclear whether or how MLL/AF4 has any direct effect on recruitment of the DOT1L protein, but increased H3K79me2/3 levels are strongly associated with MLL/AF4 binding and with high levels of gene activation (Krivtsov et al., 2008).

In this study, we explored the dependence of ALL subtypes on BCL-2 family proteins and examined the antitumor efficacy of ABT-199 in ALL, with a special focus on the *MLLr* types. Our findings indicate that direct transcriptional upregulation of *BCL-2* by MLL/AF4 confers sensitivity to the selective BCL-2 antagonist ABT-199. We also show that MLL/AF4 promotes high levels of *BCL-2* expression by binding directly to the locus and keeping it active via maintenance of H3K79me2/3 without affecting P-TEFb recruitment. This MLL/AF4 regulatory activity is specific to *BCL-2* and has no effect on other BCL-2 family members. This led to the finding that the DOT1L inhibitors sensitize *MLLr* leukemias to BCL-2 inhibition with ABT-199. Importantly, we were also able to show that ABT-199 synergizes with standard-induction-type chemotherapeutic agents, suggesting that ABT-199 could be a useful addition to *MLLr* therapeutic regimens.

RESULTS

t(4;11) ALL Is Associated with High Levels of BCL-2, BAX, and BIM

Expression of 12 pro- and anti-apoptotic proteins was studied in 186 ALL cases by reverse-phase protein analysis (RPPA). Supervised clustering demonstrated distinct differences in acute lymphoblastic leukemia (ALL) with different cytogenetic characteristics ($p < 0.005$; false discovery rate [FDR], <0.2%). Patients with 8q24 (*CMYC*) translocation (Figure 1; n = 9) expressed low levels of BCL-2 and BAX while maintaining high expression of BIM and intermediate levels of MCL-1. No specific patterns were seen in t(9;22) (Ph+) ALL. Patients with

OPEN ACCESS
CellPress

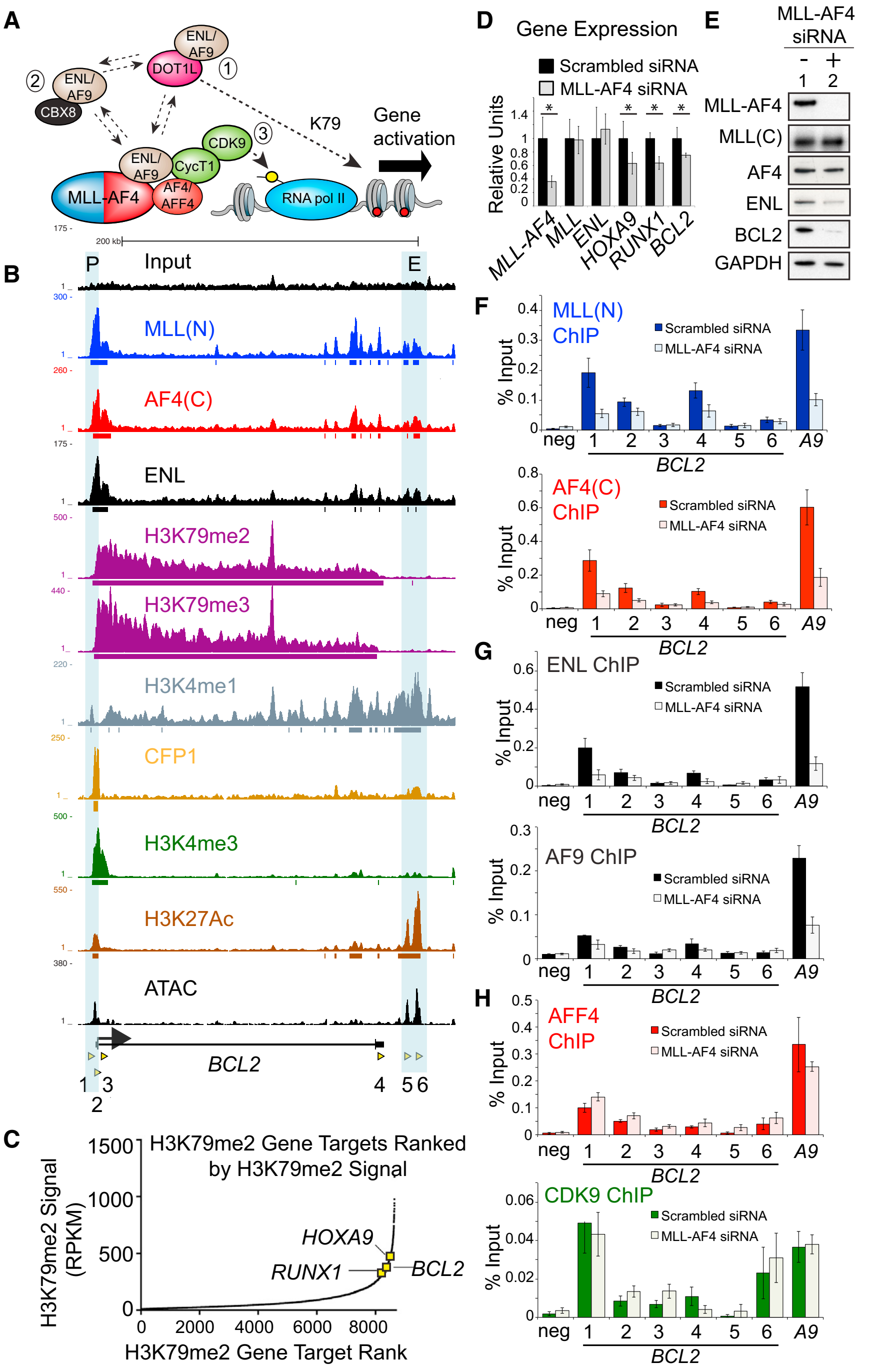

Figure 2. MLL-AF4 Binds to the *BCL-2* Gene and Keeps It Active

(A) Schematic of MLL/AF4 direct interactions and mutually exclusive complexes (dotted arrows). Gene activation could occur (1) by promoting H3K79 methylation, (2) by inhibiting CBX8 activity, or (3) AF4/AFF4 direct recruitment of P-TEFb (ie. Cyclin T1 and CDK9) and serine 2 phosphorylation of RNA polymerase II.

(B) ChIP-seq and ATAC-seq peaks in SEM cells across the *BCL-2* locus. PCR primers (1–6) used in subsequent experiments are shown as yellow arrowheads. P, promoter; E, enhancer.

(C) *BCL-2* is ranked 8,381 out of 8,647 genes (top 5%) marked with H3K79me2 in SEM cells.

(D) Real-time PCR for different targets in SEM cells treated with either a control (black bars) or an *MLL-AF4*-specific siRNA (gray bars). Signal was normalized to control treated cells and is the average of five independent knockdown experiments. Error bars indicate SD. *p < 0.02.

(E) Western blots for the indicated proteins in SEM cells treated with either a control (–) or *MLL-AF4* specific (+) siRNA.

(F–H) ChIP experiments (the average of three to five independent knockdowns) for MLL(N), AF4(C), ENL, AF9, AFF4, or CDK9 in SEM cells treated with either control (dark colored bars) or *MLL-AF4* siRNAs (light colored bars). PCR primers are as indicated in (A). *A9* = a primer set in the well-known MLL/AF4 target gene *HOXA9*, used as a positive control for ChIP. Error bars indicate SEM.

See also Figure S2.

t(4;11) (n = 12) expressed high levels of BCL-2, BAX, and BIM (Figure 1) but relatively low levels of BCL-X_L and MCL-1, although the latter differences did not reach statistical significance.

To investigate whether high BCL-2 protein levels in *MLLr* ALL is associated with high transcript levels of BCL-2, gene expression microarray data from three large cohorts of patients with ALL were analyzed (Figure S1): the St. Jude Children's Research Hospital pediatric ALL clinical trial cohort; the Eastern Cooperative Oncology Group (ECOG) Clinical Trial E2993, and the Children's Oncology Group (COG) Clinical Trial P9906. *BCL-2* mRNA expression was significantly higher in *MLLr* samples than in normal B cell controls in the St. Jude cohort (p = 0.015). *BCL-2* mRNA expression levels were significantly higher in the *MLLr* samples than in the E2A/PBX1 samples in all three cohorts (p = 0.015 for ECOG E2993, 0.0005 for COG P9906, and 0.002 for St. Jude's) and higher than in molecularly undesignated B-ALL samples in the COG P9906 study (p = 0.01). These results suggest that *BCL-2* is highly expressed in t(4;11) and other *MLLr* ALL and, therefore, that BCL-2 is a potential therapeutic target in these ALL subtypes.

MLL/AF4 Directly Controls Activation of the *BCL-2* Gene

Confirming previously published data (Guenther et al., 2008; Wilkinson et al., 2013), ChIP-seq using an MLL N-terminal antibody (Ab) (MLLN) and an AF4 C-terminal Ab (AF4C) in the *MLL/AF4*-positive B-ALL cell lines SEM and RS4;11 shows that MLL/AF4 binds to the *BCL-2* locus (Figure 2B; Figure S2A). ChIP-seq for

specific MLL/AF4 complex components (summarized in Figure 2A) shows that ENL binding closely matches the profile of MLL/AF4 binding, while H3K79me2/3 creates a broad domain across the locus (Figure 2B). Comparable to two canonical MLL/AF4 target genes, *BCL-2* is a typical MLL/AF4 target gene in that it is marked with very high levels of H3K79me2 (Figure 2C). A potential downstream enhancer—identified by its enrichment for H3K4me1, H3K27Ac, and the presence of ATAC sequencing (ATAC-seq) peaks—is also bound by the MLL/AF4/ENL complex (Figure 2B, blue shaded region marked with an E). A peak of H3K4me3 and binding of the SET1 complex (CFP1) are detected at the *BCL-2* promoter (Figure 2B), suggesting that the locus could also be regulated by H3K4 methyltransferase complexes.

For a functional analysis of MLL/AF4 activity, SEM and RS4;11 cells were treated with unique *MLL/AF4*-specific small interfering RNAs (siRNAs) (Thomas et al., 2005). MLL/AF4 siRNA knockdowns led to a reduction of *BCL-2* gene expression that was comparable to that of *HOXA9* and *RUNX1* (Figure 2D; Figure S2B) and also resulted in reduced BCL-2 protein levels in replicate experiments (Figures 2E and S2C). MLL/AF4 siRNA treatment also reduced MLL/AF4 binding to *BCL-2* (Figures 2F and S2D) without affecting wild-type MLL levels (Figures 2D, 2E, and S2B) or wild-type MLL or AF4 binding to BCL-2 (Figure S2E). Conversely, wild-type MLL knockdowns had no effect on *BCL-2* expression (Figure S2F), indicating that MLL/AF4 directly activates *BCL-2* while wild-type MLL is dispensable for *BCL-2* activation in t(4;11) cells.

MLL/AF4 knockdowns were associated with reduced ENL binding to *BCL-2* in both SEM and RS4;11 cells (Figures 2G and S2D), but there was only a marginal effect on AF9 binding, especially compared to *HOXA9* (Figure 2G). ENL mRNA levels are unaffected by MLL/AF4 knockdowns (Figure 2D), but ENL protein levels are reduced (Figure 2E), suggesting that the direct interaction between MLL/AF4 and ENL may somehow stabilize the ENL protein. In contrast to the results observed with ENL, no effect was seen on the binding of AFF4, CDK9, or Cyclin T1 in SEM cells (Figures 2H and S2G), suggesting that MLL/AF4-mediated activation of *BCL-2* does not occur through P-TEFb recruitment and stabilization. Although CBX8 binding was easily detected at *HOXC8* (a known Polycomb target in SEM cells), no change in CBX8 binding was seen at *BCL-2* (Figure S2H). Instead, MLL/AF4 knockdowns were associated with a loss of DOT1L binding at *BCL-2* (Figure S2I). Together, these results suggest that neither pathway 2 nor pathway 3 (see Figure 2A) are important components of MLL/AF4-mediated regulation of *BCL-2* but that MLL/AF4, instead, stabilizes both ENL and DOT1L binding (pathway 1) at *BCL-2*.

MLL/AF4 Controls *BCL-2* Gene Activation by Promoting Increased H3K79me2/3 Levels

To explore MLL/AF4 function further, we performed ChIP for several different histone marks in MLL/AF4 knockdowns. Consistent with the observed loss of DOT1L binding, both H3K79me2 and me3 levels are reduced across *BCL-2* (Figure 3A). We also observed reductions of H3K27Ac, especially at the downstream enhancer region of *BCL-2* (Figure 3B), whereas no significant changes in H3K4me3 levels were seen (Figure 3C).

To determine whether reduction of H3K79me2/3 levels alone could impact expression of *BCL-2* or other gene targets, SEM cells were treated with either 2 μM or 5 μM of the DOT1L inhibitor EPZ5676 for 7 days. The MLL/AF4 target genes *HOXA9*, *RUNX1*, and *BCL-2* (but not any other BCL-2 family members) all showed reduced expression with the 2- and 5-μM treatment at day 7 (Figure 3D), and this correlated with a loss of H3K79me2/3 globally and at the *BCL-2* and *HOXA9* loci (Figures 3E and 3F). Although both the 2-μM and 5-μM treatments impacted *BCL-2* expression, only the 5-μM treatment produced an observable reduction in BCL-2 protein levels (Figure 3E), comparable to that seen when *BCL-2* is directly targeted with siRNAs that can disrupt leukemic growth (Figures S3A and S3B). Together with the data in the previous section, these results indicate that the primary way that MLL/AF4 controls activation of *BCL-2* is through maintaining H3K79Me2/3 levels (see Figure 3G for a summary).

To determine whether DOT1L activity and BCL-2 inhibition were cooperative, we examined growth-inhibitory activity of two structurally distinct DOT1L inhibitors, SGC0946 and EPZ5676, combined with the selective BCL-2 inhibitor ABT-199. Consistent with our aforementioned results using 2 μM EPZ5676, treatment with 1 μM EPZ5676 had very little detectable effect on BCL-2 family protein levels (Figure S3C). However, a combined blockade of DOT1L and BCL-2 demonstrated deeper growth-inhibitory effects in t(4;11) SEMK2 cells (Figure 3H) but not in the non-*MLLr* cell line Nalm-6 (Figure S3D).

In SEM cells, MLL/AF4 is bound to both *MCL-1* and *BIM*, but not *BAX* or *BCL-2L1* (Figures 4A and 4B), and *BCL-2* family members are associated with a range of H3K79me2/3 levels (Figures 4A–4C). MLL/AF4 knockdowns have little effect on ENL or H3K79me3 levels at these loci (Figure 4B), and there is no effect on the expression levels of *MCL-1*, *BIM*, *BAX*, or *BCL-2L1* (Figures 4D and 4E). Together, these data suggest that MLL/AF4 specifically activates *BCL-2* by promoting increased H3K79me2/3 levels, while other BCL-2 family members are not dependent on MLL/AF4 or H3K79me2/3 for their expression, even though BIM and BAX are both highly expressed in t(4;11) patient samples.

BH3 Profiling Demonstrates BCL-2 Dependence of *MLLr* Primary ALL

ABT-199 is a BCL-2-selective inhibitor recently shown to be active in ALL (Alford et al., 2015). In bimolecular fluorescence complementation (BiFC) assays (Figure 5A; Vela et al., 2013), ABT-199 inhibited BCL-2/BIM interactions, and ABT-737 inhibited the interactions of BCL-2 and BCL-X_L with BIM, according to the known specificity of these inhibitors. BH3 profiling is a technique that identifies the BCL-2 protein family addictions of cancer cells based on the selective binding of BH3 proteins to specific anti-apoptotic BCL-2 family proteins (Certo et al., 2006; Chonghaile et al., 2014; Pan et al., 2014). Using ALL blast mitochondria from 16 samples consisting of primary ALL cells or patient-derived xenografts, we found a statistically significant correlation between mitochondrial sensitivity to BAD BH3 peptide (indicating BCL-2, BCL-X_L, or BCL-W dependence) and cell viability determined by half-maximal inhibitory concentration (IC_{50}) values of ABT-199 (Figure 5B; Table S2). No correlation was found for the mitochondrial response to

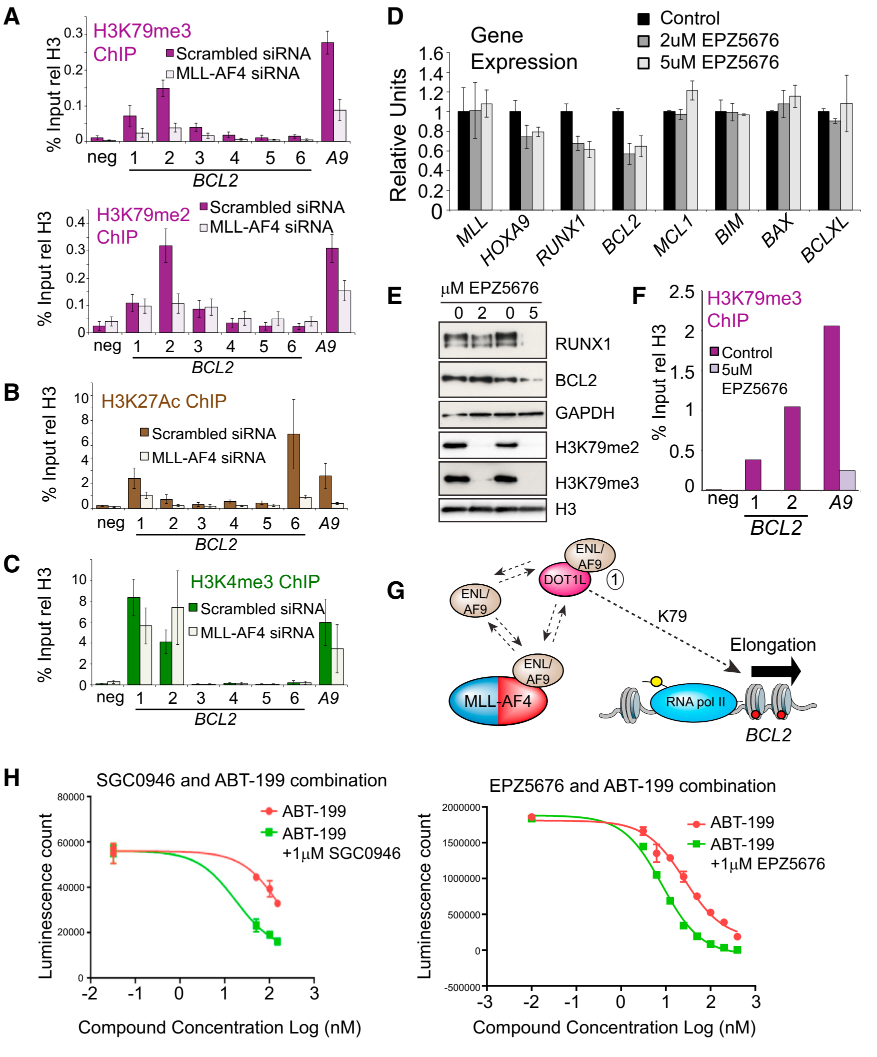

	ABT-199	ABT-199 + SGC0946 1μM
IC50	180.9	17.65

	ABT-199	ABT-199 + EPZ5676 1μM
IC50	26.3	8.33

Figure 3. MLL-AF4 Keeps *BCL-2* Active by Promoting H3K79me2/3

(A–C) ChIP experiments (average of four independent knockdowns) for H3K79me2, H3K79me3, H3K27Ac, or H3K4me3 in SEM cells treated with either control (dark bars) or *MLL-AF4* siRNAs (light bars). PCR primers are as in Figure 1. Error bars indicate SEM. rel, relative; neg, negative.

(D) SEM cells treated with 2 μM or 5 μM EPZ5676 for 7 days were subjected to real-time RT-PCR with the primers/probe sets indicated. Error bars indicate SD of four PCR replicates.

(E) Western blots of SEM cells treated with 2 or 5 μM EPZ5676 for 7 days.

(F) H3K79me3 ChIP at the *BCL-2* and *HOXA9* loci in 5 μM EPZ5676-treated SEM cells.

(G) A proposed model where MLL/AF4 stabilizes ENL protein levels and creates a local concentration of ENL that allows for dynamic exchange between an MLL-AF4:ENL complex and a DOT1L:ENL complex, potentially increasing H3K79me3 levels at the locus.

(H) SEM cells were co-treated with 1 μM of DOT1L inhibitors SGC0946 or EPZ5676 and increasing concentrations of ABT-199 (50 nM, 100 nM, and 150 nM) for 4 days (SGC0946) or 7 days (EPZ5676). Error bars indicate SEM.

See also Figure S3.

BCL-X_L-selective HRK, MCL-1-selective NOXA, or pan-BCL-2 family BIM BH3 peptides (Figure 5B). Similar correlations were obtained in a different set of adult and pediatric B-ALL samples treated with ABT-737 (Figure S4A). Next, we plotted sensitivity to the BAD BH3 peptide against sensitivity to the HRK peptide (Figure 5C). Five of the six *MLLr* samples and additional four B-ALL samples showed BCL-2 dependency (shaded area).

MLLr Cells Expressing High Levels of BCL-2 Are Susceptible to ABT-199-Induced Apoptosis In Vitro and to a Combination of ABT-199 with Chemotherapy

Our gene transcription studies and functional BH3 profiling predict that *MLLr* ALL will be particularly dependent on BCL-2 for survival. In a series of genetically diverse ALL cell lines, two t(4;11)-positive cell lines, RS4;11 and SEM-K2, exhibited high sensitivity to ABT-199 (Figures 5D and S4B). BCL-2 protein expression was highest in *MLLr* RS4;11 and SEMK2 together with the pro-B ALL cell line REH; and BCL-2, but not MCL-1 or BCL-X_L, expression correlated with sensitivity to ABT-199 ($r = -0.82$, $p = 0.008$) (Figures 5E and S4C). In a panel of 24 primary ALL samples, 92% were sensitive to ABT-199 ($IC_{50} < 1$ μM; Figures 5F and S4D), and 78% were sensitive to ABT-737. Interestingly, all five *MLLr* samples—four t(4;11) cases and one t(9;11) case—had ICs_{50} ≤ 0.1 μM. Western blot analysis of ALL blasts (n = 9) confirmed high BCL-2 protein levels (Figure S4E). High sensitivity of primary B-ALL (non-MLL) samples to BCL-2 inhibition was validated in a separate cohort of adult and pediatric samples treated with ABT-199 or dual BCL-2/BCL-X_L inhibitor ABT-263 for 8 hr (Figure S4F).

In ALL cell lines (REH, SEMK2, and RS4;11), a combination of ABT-737 or ABT-199 with chemotherapy agents (vincristine [VCR], doxorubicin [DOX], dexamethasone [DEXA], cytarabine [AraC], L-asparaginase [L-ASP]) was commonly synergistic (Table S3), with best responses observed upon combination with L-ASP (combination index values, <0.01). In six primary ALL samples, each combination produced significantly greater cytotoxic activity (Figures 6A and 6B). Consistent with the cell line data, the combination of L-ASP with ABT-199 exhibited the greatest effect.

Treatment with L-ASP in ALL patients is able to induce apoptosis specifically in lymphoblast cells. We reasoned that the nature of the L-ASP/ABT-199 synergistic interaction could be due to enhancement of apoptotic pathways in MLL/AF4 cells. Consistent with this, western blot analysis of RS4;11 or SEMK2

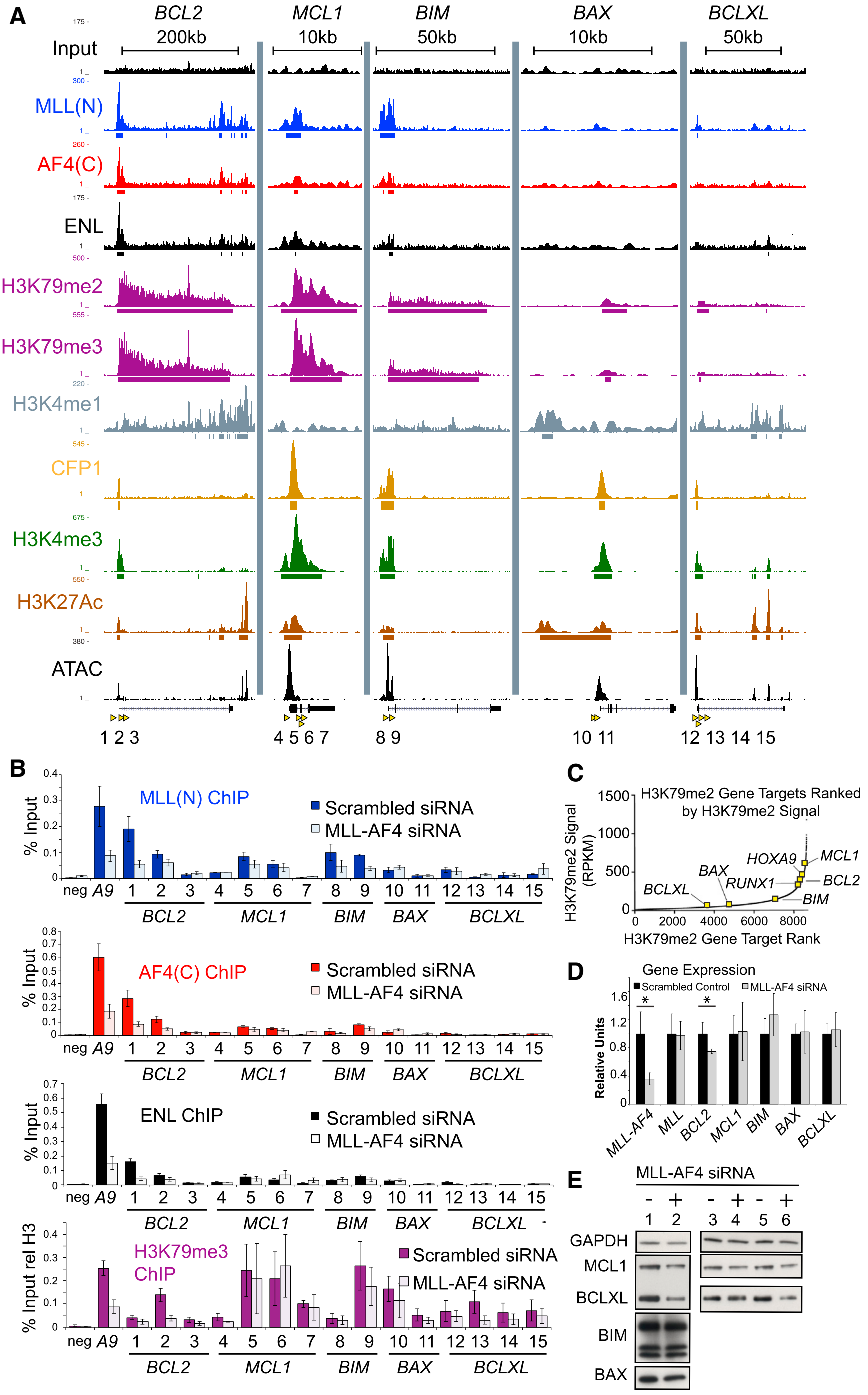

Figure 4. MLL/AF4 Does Not Directly Activate Other BCL-2 Family Genes

(A) ChIP-seq and ATAC-seq peaks in SEM cells at the loci indicated. *BCL-2* tracks are also shown for comparison purposes. PCR primers (1–15) used in subsequent experiments shown by yellow arrowheads.

(B) ChIP experiments (average of three to five independent knockdowns) in SEM cells treated with either control (dark colored bars) or *MLL-AF4* siRNAs (light colored bars). PCR primers are as in (A). *BCL-2* and *HOXA9* (*A9*) data are from Figure 2 and included for comparison purposes. Error bars indicate SEM.

(C) The same H3K79me2 ranking graph as in Figure 2C, with *BCL-2* family genes added for comparison purposes.

(D) Real-time PCR of samples from Figure 2D for *BCL-2* family genes. *MLL-AF4*, *MLL*, and *BCL-2* expression data are from Figure 2D and included for comparison purposes. Error bars indicate SD. *p < 0.02.

(E) Western blots for the indicated proteins in SEM cells treated with either a control (–) or *MLL-AF4*-specific (+) siRNA. Results shown are from three different biological replicates.

cells treated with L-ASP, VCR, or DEXA showed that each treatment significantly reduced the levels of anti-apoptotic proteins MCL-1 and BCL-X_L and the apoptotic factor FAS-associated factor 1 (FAF1; Figure 6C). Interestingly, MLL/AF4 binds and activates *FAF1* expression (Figures 6D–6F), and although FAF1 does not appear to contribute to leukemic growth (Figure S5), MLL/AF4 could contribute to high levels FAF1 protein in the cell and sensitivity to apoptosis induction.

ABT-199 Attenuates Tumor Growth of *MLLr* precursor-B ALL In Vivo and Enhances Anti-leukemia Effects of Standard Chemotherapy

Next, we investigated the anti-leukemic efficacy of ABT-199 in *MLLr* ALL in vivo using primary ALL xenograft models. In a highly aggressive primary xenograft ICN3 model, there was a marked reduction of circulating CD19-positive cells after 4 days of treatment initiated on day 45 post-cell injection (Figure 7A). Despite high levels of blasts in bone marrow (BM) on day 10 due to rapid disease progression, half of the treated mice appeared to benefit (Figure 7A). In the ALL-236-GFP model, treatment with ABT-199 for 10 days reduced tumor burden measured by bioluminescence or circulating GFP(+) cells (Figures S6A and S6B).

Next, the anti-leukemic effects of ABT-199 in combination with an induction-type regimen, VXL, comprising VCR, L-ASP, and DEXA, were evaluated in mice injected with cells from two t(4;11)-positive ALL patients (#542 and #682). Leukemic cells from both of these patients were found to be BCL-2 dependent by BH3 profiling, with sample #682 more sensitive to BIM peptide (Figure 7B). Leukemia burden (human CD45-positive cells) was determined serially in peripheral blood for case #682. Because case #542 showed a more aggressive disease course, leukemia burden was determined only on day 42 of treatment.

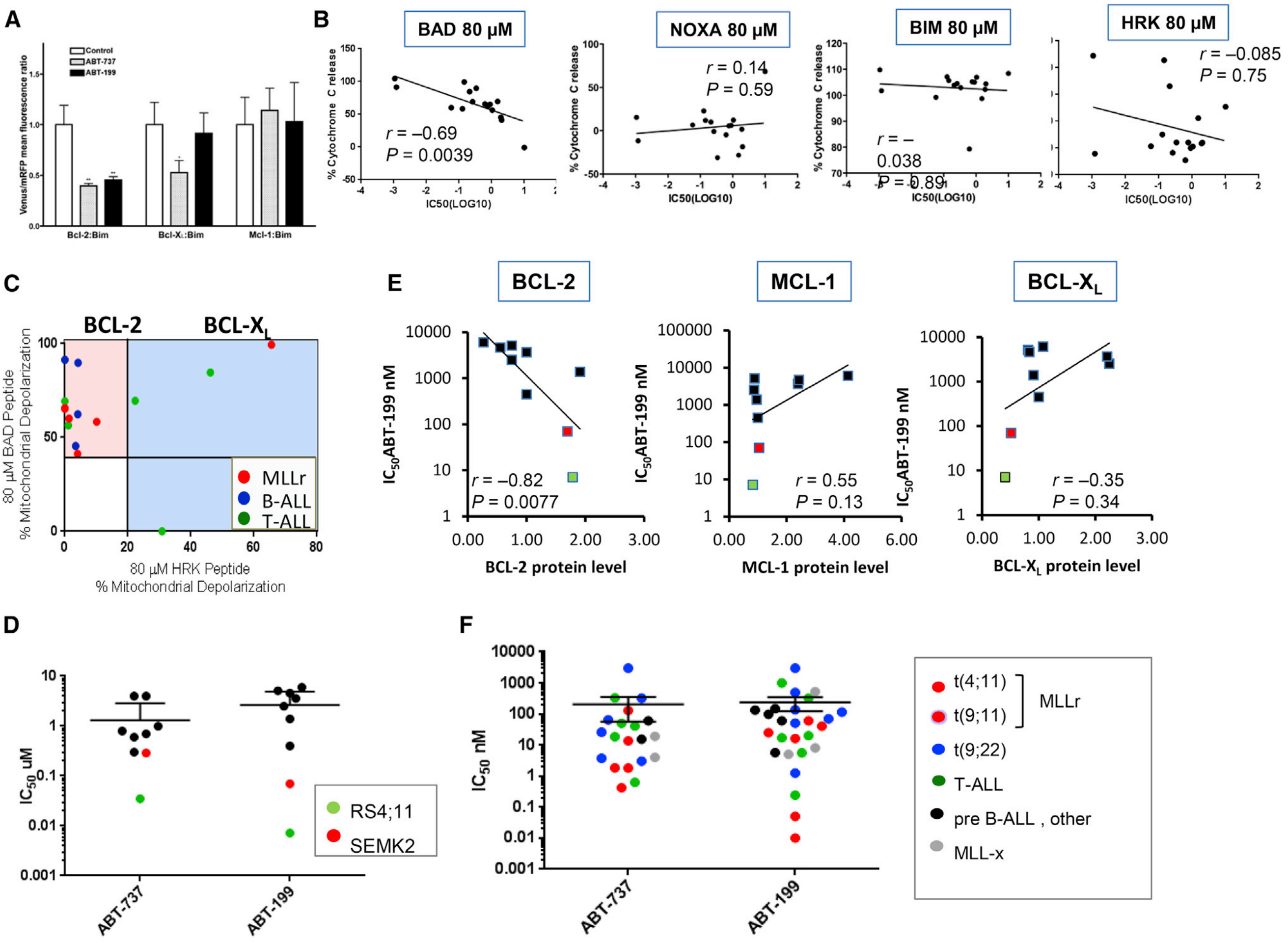

Figure 5. BH3 Profiling Reveals BCL-2 Dependence in Primary ALL

(A) BiFC analysis of interactions of anti-apoptotic proteins with BIM. Results are expressed as the fold change induced by ABT-199/ABT-737 in Venus/RFP (red fluorescent protein) ratio and are the mean ± SEM of three to six independent experiments. *p < 0.05; **p < 0.01.

(B) Levels of cytochrome *c* release from mitochondria of ALL cells exposed to the indicated BH3 peptides were correlated with cell viability IC_{50} values for ABT-199.

(C) Cytochrome *c* release induced by BAD versus HRK peptides. The pink area represents probable BCL-2 dependence, and the blue area represents BCL-X_L dependence.

(D) IC_{50} values for ABT-199 and ABT-737 in ALL cell lines. *MLL*-rearranged ALL cell lines are shown in green (RS4;11) or red (SEMK2). ALL cell line cells were treated with ABT-737 or ABT-199 for 48 hr, and the IC_{50} values calculated on the basis of viable (i.e., Annexin V-/PI-) cell numbers determined by flow cytometry.

(E) Sensitivity to ABT-199 correlates with endogenous BCL-2 protein levels but not with BCL-X_L levels in ALL cell lines. Spearman correlations were calculated based on protein expression levels relative to an internal control (β-actin) and then normalized to levels in NALM-6 cells.

(F) Primary ALL cells (n = 19) were treated with ABT-737 or ABT-199 for 24 hr, and the IC_{50} values were calculated as described earlier. MLLx, *MLL*-rearranged xenograft samples.

See also Figure S4.

In mice engrafted with #542, ABT-199 alone had minimal effects on peripheral blood leukemia burden (Figure 7C). VXL alone modestly reduced leukemia burden by 23% (p = 0.007). Unexpectedly, the combination of ABT-199 with VXL reduced leukemia burden >70% (p < 0.0001; Figure 7C), revealing striking synergy in this ALL PDX model.

In case #682 mice, both VXL and ABT-199 showed anti-leukemia effects (Figure 7D), consistent with higher sensitivity by BH3 profiling. On day 60 (Figure S6C), all six control mice carried circulating human CD45-positive cells (mean ± SD, 81.8% ± 7.1%), while one mouse from each of the six treated with ABT-199 and the six treated with VXL was leukemia free. Strikingly, circulating human CD45-positive cells were not detected in any of the mice in the combination cohort up to day 69, although positive cells were detected again on day 103 (data not shown). Two mice in the combination-treatment group died from chemotherapy-related toxicity and infection but were leukemia-free on autopsy.

DISCUSSION

Lymphoid malignancies utilize the anti-apoptotic BCL-2 family proteins to maintain viability under conditions of oncogenic stress. Because of this dependence, the leukemia cells are

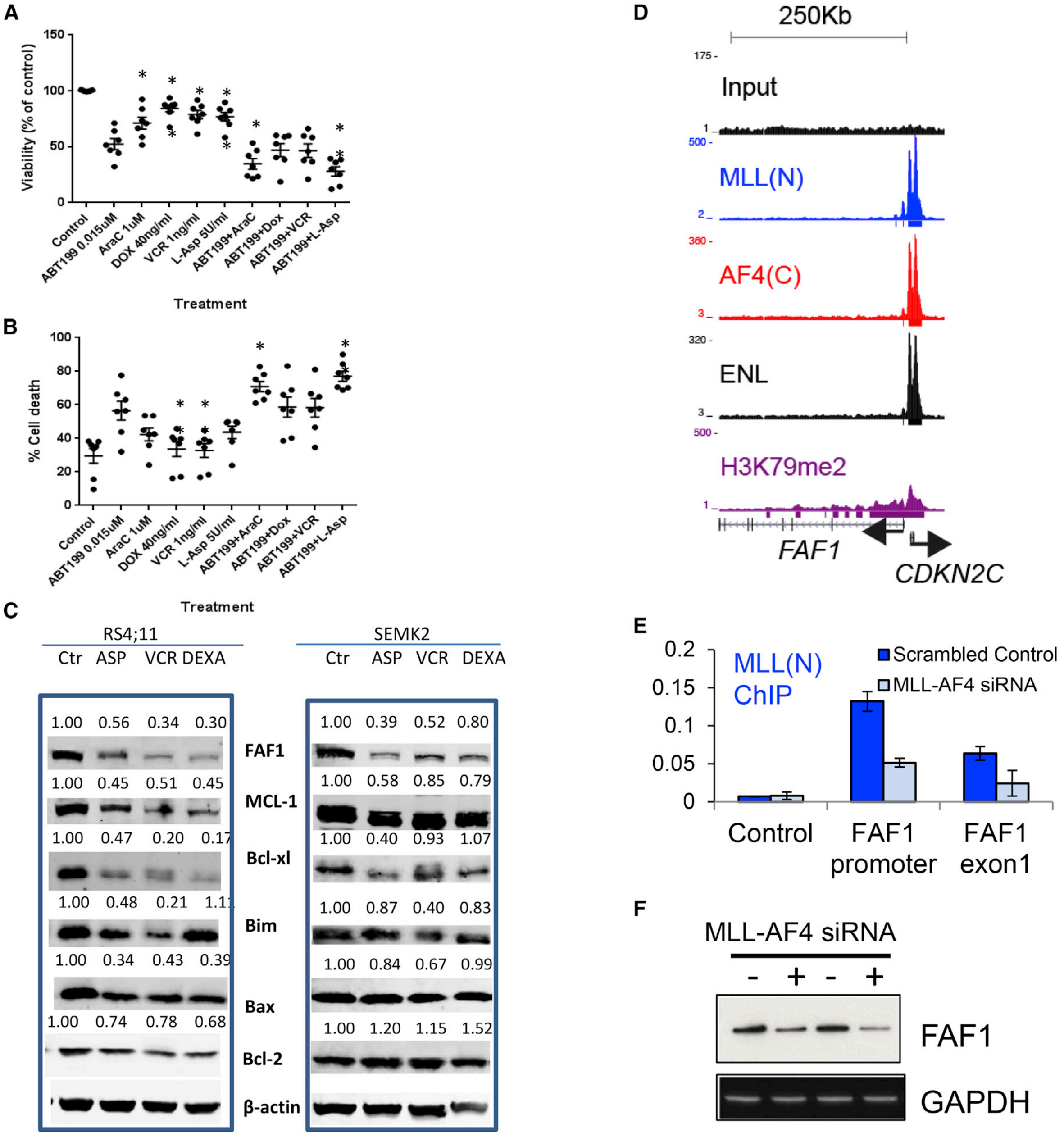

Figure 6. ABT-199 in Combination with Chemotherapy Exhibits Cytotoxic Activity against Primary ALL Cells

(A and B) ALL primary samples (n = 6) and one sample from a patient with t(4;11) biphenotypic leukemia were treated with each single reagent or in combination with ABT-199 at the following concentrations: ABT-199, 0.015 μM; AraC, 1 μM; DOX, 40 ng/ml; VCR, 1 ng/ml; and L-ASP, 5 U/ml. At 24 hr, viability (A) and cell death (B) were determined by Annexin V and 7-AAD staining. Each dot represents one sample. Error bars indicate SEM. *$p < 0.05$.

(C) Western blot analysis of RS4;11 or SEMK2 cells left untreated (Ctr) or treated for 48 or 24 hr, respectively, with: L-ASP (ASP), 2 U or 5 U (RS4;11 or SEMK2, respectively); VCR, 5 ng/ml; or DEXA, 1 μM. Representative blot of one of the three experiments that yielded similar results is shown.

(D–F) MLL/AF4 binds to the *FAF1* gene (D), and MLL/AF4 siRNA treatment reduces MLL-AF4 binding to *FAF1* (E) and reduces FAF1 protein levels (western blot, F). Error bars in (E) represent SEM for three independent knockdown experiments.

See also Figure S5 and Table S3.

susceptible to inhibition of anti-apoptotic BCL-2 family proteins. It has been reported that the BCL-2/BCL-X_L inhibitors ABT-737, ABT-263, and ABT-199 induce rapid and robust apoptosis in ALL cells, both in vitro (Alford et al., 2015; Del Gaizo Moore et al., 2008; High et al., 2010) and in human-derived xenografts (Suryani et al., 2014). In this context, we investigated the expression of BCL-2 family members in a large series of ALL patient samples by proteomic profiling. In accordance with molecular

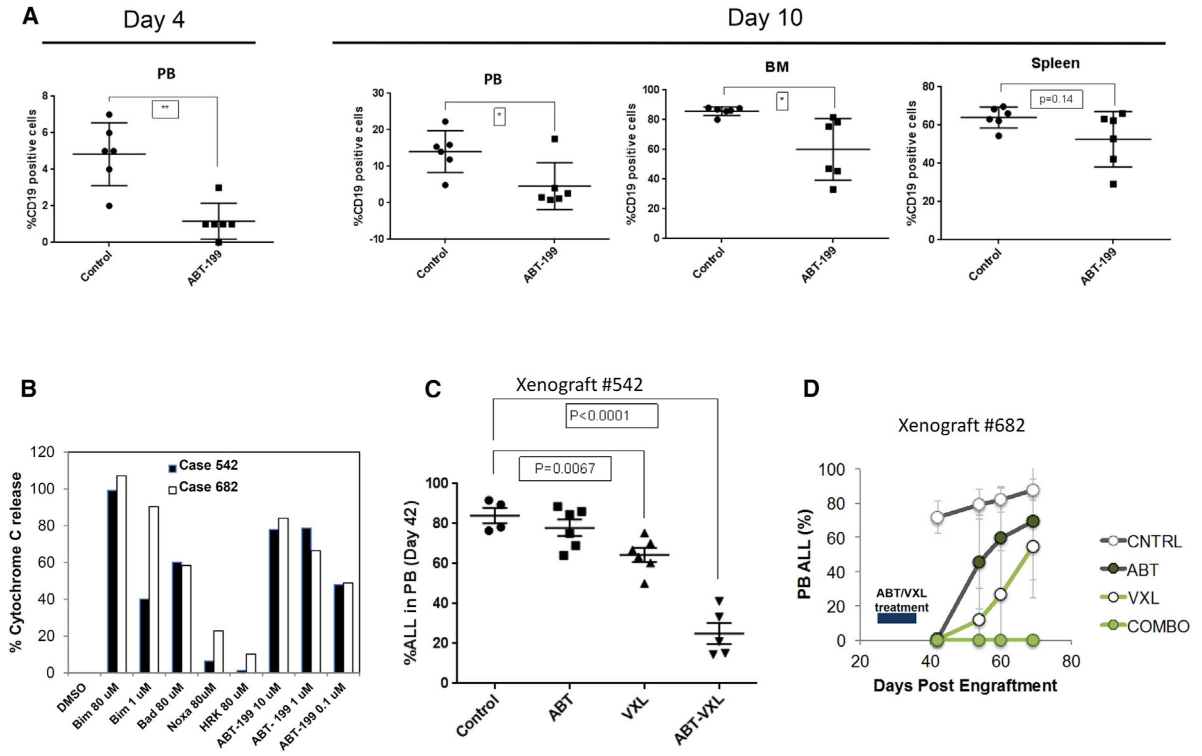

Figure 7. ABT-199 Attenuates Tumor Growth of *MLLr* Pre-B ALL and Interacts Synergistically with Induction-type Chemotherapy to Eradicate Patient-Derived ALL Cells In Vivo

(A) NSG mice were injected intravenously with ICN3 xenograft cells generated from a pediatric patient with relapsed *MLL*-rearranged pre-B-ALL. At day 45 post-injection, mice were randomized into two treatment groups (n = 6 per arm): Vehicle only; ABT-199 at 100 mg/kg/day. Leukemia burden expressed as percent human CD19$^+$ cells is shown in peripheral blood (PB) on days 4 and 10 after treatment initiation and in BM and spleen on day 10. *$p < 0.05$; **$p < 0.01$.

(B) BH3 profiling of primary derived xenografts #542 and #682. Cytochrome *c* release in response to various concentrations of BH3 peptides and ABT-199.

(C and D) Leukemia cells from two patients with t(4;11) ALL (#542 and #682) were injected into NRG mice via tail vein. On day 24 post-engraftment, mice were randomly divided into cohorts to receive VXL (VCR, DEXA, and L-ASP), ABT-199 alone, VXL+ABT-199 combination; or vehicle controls (n = 6 per arm). (C) Percentage of circulating ALL cells on day 42 post-engraftment in case #542. (D) Time course changes in percentages of circulating ALL cells in case #682. Leukemia progression was evaluated by determining the percentage of circulating human CD45-positive cells across different treatment groups. Error bars indicate SD.

See also Figure S6.

heterogeneity of ALL, the proteomic profiles were widely dispersed but closely associated with cytogenetic abnormalities. t(4;11) ALL was closely associated with high levels of BCL-2, BAX, and BIM. BCL-2 has been previously implicated in the pathogenesis of *MLLr* leukemias, and disruption of MLL-fusion-driven *BCL-2* expression has been proposed as a major mechanism of action for the bromodomain inhibitor I-BET151 (Dawson et al., 2011). Examination of publicly available gene expression datasets demonstrated that pediatric *MLLr* ALL expresses high *BCL-2* mRNA levels, consistent with previous findings in a smaller subset of pediatric ALL patients (Robinson et al., 2008). In this study, we demonstrate a direct role for MLL/AF4 in maintaining *BCL-2* expression, providing a plausible explanation for dependency of *MLLr* ALL cells on BCL-2 anti-apoptotic activity.

Interestingly, although we show that *BCL-2* expression is MLL/AF4 dependent, other *BCL-2* family members such as *MCL-1*, *BIM*, *BAX*, and *BCL-2L1* show no direct dependence on MLL/AF4 for their expression. This is despite the fact that we detect low levels of MLL/AF4 binding and H3K79me2 at both the *MCL1* and the *BIM* genes. These results highlight the fact that it is important to functionally validate ChIP-seq experiments, since the presence of binding is not necessarily functionally relevant. Importantly, we also show that wild-type MLL has no direct role in activating *BCL-2* in t(4;11) cells, indicating that *BCL-2* overexpression is an MLL/AF4-specific regulatory event. Therefore, treatment with *BCL-2* inhibitors targets a direct pathway of the MLL/AF4 driver mutation, indicating that this could be a specific vulnerability of these poor-prognosis t(4;11) leukemias.

Although the correlation between MLL/AF4 binding and H3K79 methylation levels has been observed before (Guenther et al., 2008; Krivtsov et al., 2008; Wilkinson et al., 2013), there was previously little direct evidence that MLL/AF4 acted primarily through H3K79me2/3 levels. Instead, past work has suggested that MLL/AF4 functions primarily by recruiting a large transcription elongation complex that includes AFF4, P-TEFb, ENL, AF9, and other proteins (Lin et al., 2010; Mueller et al., 2007; Yokoyama et al., 2010). It is unknown what the exact

role of H3K79me2/3 is, but recent work has suggested that it functions, in part, by disrupting SIRT1-mediated silencing (Chen et al., 2015). DOT1L inhibitors were able to reduce *BCL-2* expression, while *MCL1*, *BIM*, *BAX*, and *BCL-2L1* show almost no sensitivity to DOT1L inhibitors. This suggests that the role of H3K79me2/3 is very gene and context specific and underscores the importance of future work designed to further explore the function of this important histone mark and the complexes that regulate it.

The AF4 protein interacts directly with ENL, but the ENL:AF4 interaction and the ENL:DOT1L interactions are mutually exclusive, so how could MLL/AF4 cause recruitment of DOT1L and increased H3K79me2 levels? Structural analysis of the AF9-DOT1L interaction has shown that AF9 (and, by extension, ENL) interacts with AF4 and DOT1L through the same intrinsically disordered domain (Kuntimaddi et al., 2015; Leach et al., 2013). Intrinsic disorder allows for rapid association kinetics and the possibility of a rapid, dynamic exchange of binding partners between ENL:AF4 and ENL:DOT1L. Thus, it is possible that MLL/AF4 has a direct impact on binding of DOT1L by increasing the local concentration of ENL and/or AF9 proteins. To fully understand this possibility, further work is needed that studies the dynamic interactions of these protein complexes in vivo.

Our observation that treatment of SEM cells with DOT1L inhibitors appears to cooperate with ABT-199 treatment provides an interesting proof of principle that DOT1L inhibitors could potentially be used to sensitize *MLLr* leukemias to treatment with ABT-199. DOT1L inhibition affects gene targets other than *BCL-2* (e.g., *HOXA9* and *RUNX1*); therefore, the nature of this cooperative effect could be due to a general inhibition of a range of different MLL/AF4 targets rather than one or a few MLL/AF4 targets. However, this provides an important proof of principle that combining inhibitors that target MLL/AF4 complex activity can be used in combination with inhibitors of important MLL/AF4 target gene products.

Through BH3 profiling, we demonstrate the predominant dependence of *MLLr* B-lineage ALL cells on BCL-2, as mitochondrial sensitivity to BCL-2-selective BAD peptide was more potent than BCL-X_L-selective HRK, MCL-1-selective NOXA, and non-specific BIM peptide. Further, the robust response observed upon treatment with the BAD peptide showed excellent correlation with mitochondrial depolarization achieved in primary ALL blasts with ABT-199, confirming the on-target BCL-2-dependent activity of this agent. Notably, an additional four B-ALL and two out of five precursor T cell ALL (T-ALL) samples likewise demonstrated BCL-2 dependence. These data indicate that several phenotypically and genetically distinct ALL subtypes utilize BCL-2 as a primary pro-survival mechanism (Chonghaile et al., 2014).

Our results suggest that the presence of t(4;11) may predict response to ABT-199 in ALL. We found that the human *MLLr* B-ALL cell lines expressed the highest levels of BCL-2 protein and exhibited the greatest sensitivity to ABT-199 among the cell lines tested. Among the primary B-ALL samples tested, all *MLLr* were highly sensitive. In addition to its single-agent efficacy, ABT-199 showed beneficial results when combined with induction-type conventional chemotherapy in PDX models of B-lineage *MLLr* ALL established from patient-derived leukemia cells. In four separate in vivo experiments, short-term ABT-199 treatment (7–10 doses) had only transient anti-leukemia effects, yet it profoundly enhanced efficacy of the VXL chemotherapy regimen. The synergistic response between ABT-199 and chemotherapy treatment is likely to be complex and not due to a single factor. Previous studies have documented synergistic anti-leukemia efficacy of dual BCL-2/X_L inhibitor ABT-737 in ALL cells, including xenograft models upon combination with VXL, the regimen used here (Kang et al., 2007); this synergy was attributed to the ability of L-ASP to downregulate MCL-1 protein levels (High et al., 2010). Further, anti-mitotic agents have been shown to synergize with BH3 mimetics and reduce MCL-1 protein levels (Chen et al., 2011; Leverson et al., 2015; Tan et al., 2011; Wong et al., 2012), at least in part through phosphorylation and proteasomal degradation of MCL-1 during mitotic arrest (Wertz et al., 2011). Our data in *MLLr* cell lines support these findings, demonstrating downregulation of both MCL-1 and BCL-X_L by these agents. The regulation of the *FAF-1* locus by MLL/AF4 presents another interesting possibility. High FAF1 protein levels are able to enhance apoptosis, and FAF1 protein is degraded upon induction of apoptosis (Menges et al., 2009). This suggests that MLL/AF4-mediated FAF1 overexpression could sensitize t(4;11) cells to induction of apoptosis by factors such as L-ASP, as long as the anti-apoptotic activity of BCL-2 is also inhibited by ABT-199. This could further explain synergy between BCL-2 antagonist and standard chemotherapeutic agents used in ALL regimens seen in our studies.

In summary, our findings demonstrate that *BCL-2* is a direct target of the rearranged MLL in ALL cells, translating into BCL-2 dependence and vulnerability to selective, on-target BCL-2 inhibition by the clinically active agent ABT-199. These findings strongly advocate introduction of ABT-199, which recently demonstrated impressive efficacy in CLL trials, into the clinical armamentarium of ALL therapy.

EXPERIMENTAL PROCEDURES

Reagents

ABT-199 and ABT-737 were provided by AbbVie.

Cell Lines, siRNA, Primary Samples, and Cultures

Cell lines used for this study are detailed in the Supplemental Experimental Procedures. MLL/AF4 siRNA experiments were performed as described by Thomas et al. (2005), with differences noted in the Supplemental Experimental Procedures. All animal experiments were reviewed by institutional animal committees. All work with human samples was approved by the institutional review board at the University of Texas MD Anderson Cancer Center and the Dana-Farber Cancer Institute. Primary samples were derived from patients with ALL after informed consent was obtained in accordance with institutional guidelines set forth by the MD Anderson Cancer Center and Dana-Farber Cancer Institute. Clinical sample information is summarized in Table S1.

Western Blot Analysis

Western blot analysis was performed as previously described (Pan et al., 2014; Wilkinson et al., 2013). Antibody sources are listed under Supplemental Experimental Procedures.

RPPA

Expression of pro- and anti-apoptotic BCL-2 family proteins was studied in 186 patients diagnosed with ALL by RPPA. Antibodies used are listed in Table S4. The methodology and validation of RPPA are described elsewhere (Kornblau et al., 2011).

BiFC

Interactions between anti-apoptotic proteins BCL-2, BCL-X_L, and MCL-1 and pro-apoptotic proteins BIM and NOXA were studied by BiFC (Vela et al., 2013).

ChIP Assays and ChIP-Seq

ChIP and ChIP-seq experiments were performed as described in the Supplemental Experimental Procedures and as previously described (Milne et al., 2009; Wilkinson et al., 2013).

Intracellular BH3 Profiling of Primary ALL Cells

Intracellular BH3 profiling on primary ALL cells was performed as previously described (Pan et al., 2014).

In Vivo Murine Leukemia Models

ALL-236-GFP/LUC cells generated from pre-B-ALL with t(4;11) (Terziyska et al., 2012) and ICN3 xenograft cells generated from a child with relapsed *MLLr* pre-B-ALL (Duy et al., 2011) were injected intravenously into nonobese diabetic-severe combined immunodeficiency (NOD SCID)/IL2Rγ-KO (NSG) mice. Mice were treated with vehicle (Phosal 50 PG/polyethylene glycol [PEG]40/ethanol, 60/30/10 v/v) or ABT-199 (100 mg/kg/day) by oral gavage. For the combination model, an induction-type regimen consisting of VCR, L-ASP, and DEXA (VXL) (Szymanska et al., 2012) was used in NOD Cg-Rag1$^{tm1-Mom}$ IL2rg^{tm1Wjl}/SzJ (NRG) mice.

Statistical Analysis

Data were analyzed by the two-tailed Student's t test or the Mann-Whitney test, if appropriate. Differences were considered statistically significant at $p < 0.05$. Unless otherwise indicated, data are expressed as mean ± SD.

Additional details on experimental procedures are included in the Supplemental Experimental Procedures.

ACCESSION NUMBERS

The accession number for the data discussed in this publication is GSE: GSE74812 (http://www.ncbi.nlm.nih.gov/geo/query/acc.cgi?acc=GSE74812).

SUPPLEMENTAL INFORMATION

Supplemental Information includes Supplemental Experimental Procedures, six figures, and four tables and can be found with this article online at http://dx.doi.org/10.1016/j.celrep.2015.12.003.

AUTHOR CONTRIBUTIONS

J.M.B.: designed, performed, and analyzed experiments; wrote and edited manuscript. K.K.: wrote and edited manuscript. L.H., I.M., and R.J.: designed, performed, and analyzed experiments; edited manuscript. M.W., K.G.H., L. Golfman, T.C., O.G., E.B., Y.Q., E.O., L. Godfrey, P.N., J.K., E.B., P.Z., and E.P.: designed, performed, and analyzed experiments. H.G., K.R.C., and N.Z.: analyzed data. L.D. and H.M.: performed and analyzed experiments. D.A.T., M.A., S.O., H.M.K.: edited manuscript. J.D.L., M.M., and I.J.: provided reagents and edited manuscript. S.M.K.: designed experiments and analyzed data. P.A.Z.-M., T.A.M., J.C.M., and A.L.: designed and analyzed experiments and edited manuscript. T.M. and M.K.: designed and analyzed experiments; wrote and edited manuscript.

CONFLICTS OF INTEREST

J.D.L. has ownership interest in AbbVie, Inc.; M.A. has commercial research support from Daiichi-Sankyo, has received honoraria from the speakers' bureau of Tetralogic, and is a consultant/advisory board member of Amgen and Eutropics; M.K. has received a commercial research grant from AbbVie, Inc., and is a consultant/advisory board member of the same; A.L. is a consultant/advisory board member of AbbVie Pharmaceuticals. No potential conflicts of interest were disclosed by the other authors.

ACKNOWLEDGMENTS

The work in this grant performed by T.M., L.G., J.K., P.N., and E.B. was funded by Medical Research Council (MRC) Molecular Haematology Unit grant MC_UU_12009/6. We thank the High-Throughput Genomics Group at the Wellcome Trust Centre for Human Genetics (funded by Wellcome Trust grant reference 090532/Z/09/Z) and the Computational Biology Research Group (CBRG), Radcliffe Department of Medicine, at the University of Oxford. This work was supported, in part, by AbbVie Inc. and by NIH/NCI grant P30 CA016672 to the University of Texas MD Anderson Cancer Center (MDACC).

Received: May 7, 2015
Revised: October 21, 2015
Accepted: November 19, 2015
Published: December 17, 2015

REFERENCES

Alford, S.E., Kothari, A., Loeff, F.C., Eichhorn, J.M., Sakurikar, N., Goselink, H.M., Saylors, R.L., Jedema, I., Falkenburg, J.H., and Chambers, T.C. (2015). BH3 inhibitor sensitivity and Bcl-2 dependence in primary acute lymphoblastic leukemia cells. Cancer Res. *75*, 1366–1375.

Andersson, A.K., Ma, J., Wang, J., Chen, X., Gedman, A.L., Dang, J., Nakitandwe, J., Holmfeldt, L., Parker, M., Easton, J., et al.; St. Jude Children's Research Hospital–Washington University Pediatric Cancer Genome Project (2015). The landscape of somatic mutations in infant MLL-rearranged acute lymphoblastic leukemias. Nat. Genet. *47*, 330–337.

Beldjord, K., Chevret, S., Asnafi, V., Huguet, F., Boulland, M.L., Leguay, T., Thomas, X., Cayuela, J.M., Grardel, N., Chalandon, Y., et al.; Group for Research on Adult Acute Lymphoblastic Leukemia (GRAALL) (2014). Oncogenetics and minimal residual disease are independent outcome predictors in adult patients with acute lymphoblastic leukemia. Blood *123*, 3739–3749.

Biswas, D., Milne, T.A., Basrur, V., Kim, J., Elenitoba-Johnson, K.S., Allis, C.D., and Roeder, R.G. (2011). Function of leukemogenic mixed lineage leukemia 1 (MLL) fusion proteins through distinct partner protein complexes. Proc. Natl. Acad. Sci. USA *108*, 15751–15756.

Bursen, A., Schwabe, K., Rüster, B., Henschler, R., Ruthardt, M., Dingermann, T., and Marschalek, R. (2010). The AF4.MLL fusion protein is capable of inducing ALL in mice without requirement of MLL.AF4. Blood *115*, 3570–3579.

Certo, M., Del Gaizo Moore, V., Nishino, M., Wei, G., Korsmeyer, S., Armstrong, S.A., and Letai, A. (2006). Mitochondria primed by death signals determine cellular addiction to antiapoptotic BCL-2 family members. Cancer Cell *9*, 351–365.

Chen, J., Jin, S., Abraham, V., Huang, X., Liu, B., Mitten, M.J., Nimmer, P., Lin, X., Smith, M., Shen, Y., et al. (2011). The Bcl-2/Bcl-X(L)/Bcl-w inhibitor, navitoclax, enhances the activity of chemotherapeutic agents in vitro and in vivo. Mol. Cancer Ther. *10*, 2340–2349.

Chen, C.W., Koche, R.P., Sinha, A.U., Deshpande, A.J., Zhu, N., Eng, R., Doench, J.G., Xu, H., Chu, S.H., Qi, J., et al. (2015). DOT1L inhibits SIRT1-mediated epigenetic silencing to maintain leukemic gene expression in MLL-rearranged leukemia. Nat. Med. *21*, 335–343.

Chonghaile, T.N., Roderick, J.E., Glenfield, C., Ryan, J., Sallan, S.E., Silverman, L.B., Loh, M.L., Hunger, S.P., Wood, B., DeAngelo, D.J., et al. (2014). Maturation stage of T-cell acute lymphoblastic leukemia determines BCL-2 versus BCL-XL dependence and sensitivity to ABT-199. Cancer Discov. *4*, 1074–1087.

Dawson, M.A., Prinjha, R.K., Dittmann, A., Giotopoulos, G., Bantscheff, M., Chan, W.I., Robson, S.C., Chung, C.W., Hopf, C., Savitski, M.M., et al. (2011). Inhibition of BET recruitment to chromatin as an effective treatment for MLL-fusion leukaemia. Nature *478*, 529–533.

Del Gaizo Moore, V., Schlis, K.D., Sallan, S.E., Armstrong, S.A., and Letai, A. (2008). BCL-2 dependence and ABT-737 sensitivity in acute lymphoblastic leukemia. Blood *111*, 2300–2309.

Dreyer, Z.E., Hilden, J.M., Jones, T.L., Devidas, M., Winick, N.J., Willman, C.L., Harvey, R.C., Chen, I.M., Behm, F.G., Pullen, J., et al. (2015). Intensified chemotherapy without SCT in infant ALL: results from COG P9407 (cohort 3). Pediatr. Blood Cancer *62*, 419–426.

Duy, C., Hurtz, C., Shojaee, S., Cerchietti, L., Geng, H., Swaminathan, S., Klemm, L., Kweon, S.M., Nahar, R., Braig, M., et al. (2011). BCL6 enables Ph+ acute lymphoblastic leukaemia cells to survive BCR-ABL1 kinase inhibition. Nature *473*, 384–388.

Guenther, M.G., Lawton, L.N., Rozovskaia, T., Frampton, G.M., Levine, S.S., Volkert, T.L., Croce, C.M., Nakamura, T., Canaani, E., and Young, R.A. (2008). Aberrant chromatin at genes encoding stem cell regulators in human mixed-lineage leukemia. Genes Dev. *22*, 3403–3408.

High, L.M., Szymanska, B., Wilczynska-Kalak, U., Barber, N., O'Brien, R., Khaw, S.L., Vikstrom, I.B., Roberts, A.W., and Lock, R.B. (2010). The Bcl-2 homology domain 3 mimetic ABT-737 targets the apoptotic machinery in acute lymphoblastic leukemia resulting in synergistic in vitro and in vivo interactions with established drugs. Mol. Pharmacol. *77*, 483–494.

Kang, M.H., Kang, Y.H., Szymanska, B., Wilczynska-Kalak, U., Sheard, M.A., Harned, T.M., Lock, R.B., and Reynolds, C.P. (2007). Activity of vincristine, L-ASP, and dexamethasone against acute lymphoblastic leukemia is enhanced by the BH3-mimetic ABT-737 in vitro and in vivo. Blood *110*, 2057–2066.

Kornblau, S.M., Qiu, Y.H., Zhang, N., Singh, N., Faderl, S., Ferrajoli, A., York, H., Qutub, A.A., Coombes, K.R., and Watson, D.K. (2011). Abnormal expression of FLI1 protein is an adverse prognostic factor in acute myeloid leukemia. Blood *118*, 5604–5612.

Krivtsov, A.V., Feng, Z., Lemieux, M.E., Faber, J., Vempati, S., Sinha, A.U., Xia, X., Jesneck, J., Bracken, A.P., Silverman, L.B., et al. (2008). H3K79 methylation profiles define murine and human MLL-AF4 leukemias. Cancer Cell *14*, 355–368.

Kuntimaddi, A., Achille, N.J., Thorpe, J., Lokken, A.A., Singh, R., Hemenway, C.S., Adli, M., Zeleznik-Le, N.J., and Bushweller, J.H. (2015). Degree of recruitment of DOT1L to MLL-AF9 defines level of H3K79 Di- and tri-methylation on target genes and transformation potential. Cell Rep. *11*, 808–820.

Leach, B.I., Kuntimaddi, A., Schmidt, C.R., Cierpicki, T., Johnson, S.A., and Bushweller, J.H. (2013). Leukemia fusion target AF9 is an intrinsically disordered transcriptional regulator that recruits multiple partners via coupled folding and binding. Structure *21*, 176–183.

Leverson, J.D., Phillips, D.C., Mitten, M.J., Boghaert, E.R., Diaz, D., Tahir, S.K., Belmont, L.D., Nimmer, P., Xiao, Y., Ma, X.M., et al. (2015). Exploiting selective BCL-2 family inhibitors to dissect cell survival dependencies and define improved strategies for cancer therapy. Sci. Transl. Med. *7*, 279ra40.

Lin, C., Smith, E.R., Takahashi, H., Lai, K.C., Martin-Brown, S., Florens, L., Washburn, M.P., Conaway, J.W., Conaway, R.C., and Shilatifard, A. (2010). AFF4, a component of the ELL/P-TEFb elongation complex and a shared subunit of MLL chimeras, can link transcription elongation to leukemia. Mol. Cell *37*, 429–437.

Maethner, E., Garcia-Cuellar, M.P., Breitinger, C., Takacova, S., Divoky, V., Hess, J.L., and Slany, R.K. (2013). MLL-ENL inhibits polycomb repressive complex 1 to achieve efficient transformation of hematopoietic cells. Cell Rep. *3*, 1553–1566.

Menges, C.W., Altomare, D.A., and Testa, J.R. (2009). FAS-associated factor 1 (FAF1): diverse functions and implications for oncogenesis. Cell Cycle *8*, 2528–2534.

Milne, T.A., Zhao, K., and Hess, J.L. (2009). Chromatin immunoprecipitation (ChIP) for analysis of histone modifications and chromatin-associated proteins. Methods Mol. Biol. *538*, 409–423.

Mohan, M., Herz, H.M., Takahashi, Y.H., Lin, C., Lai, K.C., Zhang, Y., Washburn, M.P., Florens, L., and Shilatifard, A. (2010). Linking H3K79 trimethylation to Wnt signaling through a novel Dot1-containing complex (DotCom). Genes Dev. *24*, 574–589.

Molica, S. (2015). Highlights in the treatment of chronic lymphocytic leukemia from the 2014 meeting of the American Society of Hematology. Expert Rev. Hematol. *8*, 277–281.

Mueller, D., Bach, C., Zeisig, D., Garcia-Cuellar, M.P., Monroe, S., Sreekumar, A., Zhou, R., Nesvizhskii, A., Chinnaiyan, A., Hess, J.L., and Slany, R.K. (2007). A role for the MLL fusion partner ENL in transcriptional elongation and chromatin modification. Blood *110*, 4445–4454.

Pan, R., Hogdal, L.J., Benito, J.M., Bucci, D., Han, L., Borthakur, G., Cortes, J., DeAngelo, D.J., Debose, L., Mu, H., et al. (2014). Selective BCL-2 inhibition by ABT-199 causes on-target cell death in acute myeloid leukemia. Cancer Discov. *4*, 362–375.

Pieters, R., Schrappe, M., De Lorenzo, P., Hann, I., De Rossi, G., Felice, M., Hovi, L., LeBlanc, T., Szczepanski, T., Ferster, A., et al. (2007). A treatment protocol for infants younger than 1 year with acute lymphoblastic leukaemia (Interfant-99): an observational study and a multicentre randomised trial. Lancet *370*, 240–250.

Robinson, B.W., Behling, K.C., Gupta, M., Zhang, A.Y., Moore, J.S., Bantly, A.D., Willman, C.L., Carroll, A.J., Adamson, P.C., Barrett, J.S., and Felix, C.A. (2008). Abundant anti-apoptotic BCL-2 is a molecular target in leukaemias with t(4;11) translocation. Br. J. Haematol. *141*, 827–839.

Souers, A.J., Leverson, J.D., Boghaert, E.R., Ackler, S.L., Catron, N.D., Chen, J., Dayton, B.D., Ding, H., Enschede, S.H., Fairbrother, W.J., et al. (2013). ABT-199, a potent and selective BCL-2 inhibitor, achieves antitumor activity while sparing platelets. Nat. Med. *19*, 202–208.

Suryani, S., Carol, H., Chonghaile, T.N., Frismantas, V., Sarmah, C., High, L., Bornhauser, B., Cowley, M.J., Szymanska, B., Evans, K., et al. (2014). Cell and molecular determinants of in vivo efficacy of the BH3 mimetic ABT-263 against pediatric acute lymphoblastic leukemia xenografts. Clin. Cancer Res. *20*, 4520–4531.

Szymanska, B., Wilczynska-Kalak, U., Kang, M.H., Liem, N.L., Carol, H., Boehm, I., Groepper, D., Reynolds, C.P., Stewart, C.F., and Lock, R.B. (2012). Pharmacokinetic modeling of an induction regimen for in vivo combined testing of novel drugs against pediatric acute lymphoblastic leukemia xenografts. PLoS ONE *7*, e33894.

Tan, N., Malek, M., Zha, J., Yue, P., Kassees, R., Berry, L., Fairbrother, W.J., Sampath, D., and Belmont, L.D. (2011). Navitoclax enhances the efficacy of taxanes in non-small cell lung cancer models. Clin. Cancer Res. *17*, 1394–1404.

Terziyska, N., Castro Alves, C., Groiss, V., Schneider, K., Farkasova, K., Ogris, M., Wagner, E., Ehrhardt, H., Brentjens, R.J., zur Stadt, U., et al. (2012). In vivo imaging enables high resolution preclinical trials on patients' leukemia cells growing in mice. PLoS ONE *7*, e52798.

Thomas, M., Gessner, A., Vornlocher, H.P., Hadwiger, P., Greil, J., and Heidenreich, O. (2005). Targeting MLL-AF4 with short interfering RNAs inhibits clonogenicity and engraftment of t(4;11)-positive human leukemic cells. Blood *106*, 3559–3566.

Urtishak, K.A., Edwards, A.Y., Wang, L.S., Hudome, A., Robinson, B.W., Barrett, J.S., Cao, K., Cory, L., Moore, J.S., Bantly, A.D., et al. (2013). Potent obatoclax cytotoxicity and activation of triple death mode killing across infant acute lymphoblastic leukemia. Blood *121*, 2689–2703.

Vaillant, F., Merino, D., Lee, L., Breslin, K., Pal, B., Ritchie, M.E., Smyth, G.K., Christie, M., Phillipson, L.J., Burns, C.J., et al. (2013). Targeting BCL-2 with the BH3 mimetic ABT-199 in estrogen receptor-positive breast cancer. Cancer Cell *24*, 120–129.

Vandenberg, C.J., and Cory, S. (2013). ABT-199, a new Bcl-2-specific BH3 mimetic, has in vivo efficacy against aggressive Myc-driven mouse lymphomas without provoking thrombocytopenia. Blood *121*, 2285–2288.

Vela, L., Gonzalo, O., Naval, J., and Marzo, I. (2013). Direct interaction of Bax and Bak proteins with Bcl-2 homology domain 3 (BH3)-only proteins in living cells revealed by fluorescence complementation. J. Biol. Chem. *288*, 4935–4946.

Wertz, I.E., Kusam, S., Lam, C., Okamoto, T., Sandoval, W., Anderson, D.J., Helgason, E., Ernst, J.A., Eby, M., Liu, J., et al. (2011). Sensitivity to antitubulin chemotherapeutics is regulated by MCL1 and FBW7. Nature *471*, 110–114.

Wilkinson, A.C., Ballabio, E., Geng, H., North, P., Tapia, M., Kerry, J., Biswas, D., Roeder, R.G., Allis, C.D., Melnick, A., et al. (2013). RUNX1 is a key target in t(4;11) leukemias that contributes to gene activation through an AF4-MLL complex interaction. Cell Rep. *3*, 116–127.

Wong, M., Tan, N., Zha, J., Peale, F.V., Yue, P., Fairbrother, W.J., and Belmont, L.D. (2012). Navitoclax (ABT-263) reduces Bcl-x(L)-mediated chemoresistance in ovarian cancer models. Mol. Cancer Ther. *11*, 1026–1035.

Yokoyama, A., Lin, M., Naresh, A., Kitabayashi, I., and Cleary, M.L. (2010). A higher-order complex containing AF4 and ENL family proteins with P-TEFb facilitates oncogenic and physiologic MLL-dependent transcription. Cancer Cell *17*, 198–212.

Cell Reports

Supplemental Information

MLL-Rearranged Acute Lymphoblastic Leukemias Activate BCL-2 through H3K79 Methylation and Are Sensitive to the BCL-2-Specific Antagonist ABT-199

Juliana M. Benito, Laura Godfrey, Kensuke Kojima, Leah Hogdal, Mark Wunderlich, Huimin Geng, Isabel Marzo, Karine G. Harutyunyan, Leonard Golfman, Phillip North, Jon Kerry, Erica Ballabio, Triona Ní Chonghaile, Oscar Gonzalo, Yihua Qiu, Irmela Jeremias, LaKiesha Debose, Eric O'Brien, Helen Ma, Ping Zhou, Rodrigo Jacamo, Eugene Park, Kevin R. Coombes, Nianxiang Zhang, Deborah A. Thomas, Susan O'Brien, Hagop M. Kantarjian, Joel D. Leverson, Steven M. Kornblau, Michael Andreeff, Markus Müschen, Patrick A. Zweidler-McKay, James C. Mulloy, Anthony Letai, Thomas A. Milne, and Marina Konopleva

Table S1, Related to Experimental Procedures. Primary samples information

Arbitrary sample #	Diagnosis	Source	% Blasts	Cytogenetics	Assay
1	Rel/Refr T-ALL	PB	95	del(5q), del(6q), and i(17q)	BH3 prof, comb, WB
2	Rel/Refr B-ALL	PB	91	insufficient metaphases	BH3 prof, single treat, WB
3	Rel T-ALL	PB	9	del(6)	BH3 prof, single treat
4	Refr T-ALL	BM	25	del(5q), del(7q) and +21	BH3 prof, single treat, WB
5	Refr preB-ALL	PB	100	t(4;11)	single treat, WB
6	New B-ALL	PB	94	t(9;22)	BH3 prof, comb
7	T-ALL	PB	95	inv(17)	BH3 prof
8	T-ALL	PB	74	ND	BH3 prof, WB
9	New B-ALL	BM	94	t(9;22)	BH3 prof, comb, WB
11	Rel/Refr B-ALL	PB	90	Diploid	BH3 prof, comb, WB
12	ICN3 (P9)	Xenograft		MLL-rearranged ALL	BH3 prof
13	ICN13	Xenograft		MLL-rearranged ALL	BH3 prof
14	ALL-236	Xenograft		MLL-rearranged ALL	BH3 prof
15	ALL 542	Xenograft		t(4;11)	BH3 prof
16	ALL 682	Xenograft		MLL-rearranged ALL	BH3 prof
17	PS 2013-25	Xenograft		N/A	BH3 prof
18	New PreB-ALL	BM	94%	46,XY,del(9)(q13),add(10)(q24)[3]/46,XY[4]	BH3 prof, Single treat
19	New PreB-ALL	BM	88%	45,XX,der(7)t(7;9)(p11;q11),-9,t(9;22)(q34;q11.2)[12]/46,idem,+der(22), t(9;22)[cp2]/46,XX[6], Ph+ ALL,	BH3 prof, Single treat
20	New PreB-ALL	BM	89%	46,XY,t(4;19)(q31;q13)[20].nuc ish(ABLx2)[100]	BH3 prof, Single treat
21	New PreB-ALL	BM	81%	46,XY[20].nuc ish(ABL1,BCR)x2[100]	BH3 prof, Single treat
22	New B-ALL	BM	74%	45,XX,-7,t(9;22;16)(q34;q11;q24)[cp19]/46,XX[1]	BH3 prof, Single treat
23	Pediatric New B-ALL	BM	97%	56XY,+X,dup(1)(q21q42),+4,+6,+9,+10,+14,+14,+17,+21,+21	BH3 prof, Single treat
24	Pediatric New B-ALL	BM	96%	54-55XX,+X,+4,+4,i(7)9q10),+15,i(17)(q10),-19,+21,+22,+1-3 marker	BH3 prof, Single treat
25	Pediatric New B-ALL	BM	90%	53-55XXX,add(1)(q4?2),+4,+6,+10,+14,+17,+21,+21	BH3 prof, Single treat
26	Pediatric New B-ALL	BM	89%	55XX,+X,+4,+6,+10,+14,+18,+18,+19,+21,+21	BH3 prof, Single treat
27	Pediatric New B-ALL	BM	95%	Normal	BH3 prof, Single treat

28	Pediatric New B-ALL	BM	80%	51XX,+X,+4,-7,+9,+14,+der(7)t(7;17)(q11.2;p11.2)+21	BH3 prof, Single treat
29	Pediatric New B-ALL	BM	73%	normal	BH3 prof, Single treat
30	Rel preB-ALL	PB	69%	t(4;11)	Single treat
31	New preB-ALL	PB	90%	Diploid	Single treat
32	Rel/refr T-ALL	PB	99%	del(9)	Single treat
33	Rel/refr preB-ALL	PB	56%	t(2;9)(p23;p22),add(7)(q36),del(13)(q12q14),del(17)(p11.2),+mar[19]	Single treat, WB
34	Rel/Refr T-ALL	PB	95%	del(5q), del(6q), and i(17q)	Single treat
35	New preB-ALL	PB	62%	t(9;11)	Single treat
36	Rel/refr preB-ALL ph(-)	PB	31%	t(4;11)	Single treat
37	Rel/Refr preB-ALL	PB	95%	t(9;22)	Single treat
38	Rel preB-ALL	PB	86%	insufficient metaphases	Single treat
39	New preB-ALL. Ph(+)	PB	87%	t(9;22)	Single treat
40	New preB-ALL	PB	46%	Diploid	Single treat
41	Rel/Refr T-ALL	BM	6%	Diploid	Single treat
42	New preB-ALL	PB	58%	t(9;22)	Single treat
43	Refr T-ALL	PB	86%	del (6)	Single treat
44	New preB-ALL	PB	83%	t(9;22)	Single treat, WB
45	Rel/Refr AML (MLL)	PB	80%	46,XY,t(6;11)(q27;q23)[20]; MLL	Comb
46	Rel/Refr B-ALL	BM	97%	t(14;19)(q32;p13.1),del(17)(p11.2),-20,+mar[9]	Comb, WB
47	New B-ALL	PB	87%	t(9;22)(q34;q11.2)	Comb
48	New B-ALL	BM	63%	t(4;11)(q21;q23) (FISH-MLL+)	Comb

BH3 prof: BH3 profiling; single treat: single treatment; WB: western blot; comb: combination

Table S2, Related to Figure 5. BH3 profiling of primary ALL samples and primary derived xenografts.

	B-ALL				T-ALL					MLL-r					
Sample number	6	9	11	2	1	3	4	7	8	12	13	14	15	16	17
DMSO	0	0	0	0	0	0	0	0	0	0	0	0	0	0	0
Bim 80 uM	102.89	101.73	102.22	105.2	106.93	108.41	104.48	103.87	98.7	79.33	105.66	105.02	99.17	107.1	104.34
Bim 1 uM	98.82	97.01	53.53	95.77	69.92	103.65	83.05	91.81	73.78	18.45	99.29	53.62	40.14	90.41	57.19
Bim 0.3 uM	67.9	65.12	17.07	47.55	17.69	86.8	26.25	64.55	25.49	-3	81.71	16.85	20.81	47.03	-7.84
Bim 0.1 uM	20.97	17.8	11.25	8.96	12.04	66.84	4.51	36.06	0.17	-9.21	44.56	6.47	14.16	16.65	-11.95
Bim 0.03 uM	9.98	-6.19	4.64	2.12	12.23	47.38	2.95	17.36	-8.65	-5.62	25.26	3.22	6.11	10.12	-10.68
Bad 80 uM	89.8	91.37	45.42	62.41	56.48	-0.69	69.3	84.66	69.53	65.73	99.41	65.32	60.11	58.38	41.33
Bad 30 uM	91.7	93.02	49.48	62.31	53	7.47	73.59	89.57	76.14	65.97	99.27	66.21	64.23	71.48	47.09
Bad 8 uM	86.6	93	53.56	57.25	52.5	12.96	71.66	90.91	74.06	65.16	98.9	60.99	72.9	66.56	49.41
Noxa 80uM	-31.15	-11.75	1.57	5.54	12.35	68.16	-1	10.09	-28.31	-4.76	11.92	6.07	6.52	22.83	-18.49
HRK 80 uM	4.18	-4.19	3.43	4.12	1.12	30.88	-3.55	46.21	22.34	-9.09	65.6	0.02	1.34	10.12	4.07
Puma 80 uM	93.75	95.23	58.86	90.19	79.67	85.16	62.8	91.87	77.41	45.55	96.21	74.94	47.06	62.31	49.29
Puma 8 uM	82.84	91.33	38.91	66	56.68	58.67	12.41	88.89	76.27	32.03	93.64	49.93	45.67	76.29	36.02
Puma 0.8 uM	28.03	47.7	8.81	17.67	18.39	26.81	76.58	50.96	43.41	-3.78	65.85	9.91	12.08	28.13	-11.31
ABT-199 10 uM	86.77	94.12	77.68	78.83	81.49	-21.1	81	96.55	77.74	70.28	99.88	73.34	77.89	84.09	74.9
ABT- 199 1 uM	73.25	91.76	27.2	50.12	40.57	7.82	65.97	70.47	40.64	57.22	84.08	49.93	78.68	66.39	39.94
ABT-199 0.1 uM	28.62	78.5	20.44	28.39	23.62	12.24	39.04	39.83	15.93	33.62	51.22	29.54	48.01	48.86	10.05
ABT-737 1 uM	82.79	93.29	44.95	54.53	35.73	11.52	67.12	85.51	73.3	49.81	91.79	56.15	64.31	64.21	52.04
ABT-737 0.1 uM	73.17	86.91	32.19	43.13	23.81	7.02	49.02	71.79	54.07	32.72	85.2	32.56	50.22	59.5	20.42
Puma2a 80 uM	-15.5	-14.93	10.32	-14.27	11.42	-4.94	-24.11	8.23	46.4	-13.24	0	5.85	-0.14	-1.33	-14.33

Cytochrome C release in response to various concentrations of BH3 peptides and ABT-737 or ABT-199 in B-ALL samples, T-ALL samples and primary derived MLLr xenografts. For clinical information, please refer to Table S1.

Table S3, Related to Figure 6. Combination indices for indicated drug combinations at ED50, ED75 and ED90.

Cell line	Combination	ED50	ED75	ED90
REH	VCR/ABT-737	0.38	0.29	0.22
	VCR/ABT-199	0.73	0.40	0.22
	AraC/ABT-737	2.50	1.02	0.41
	AraC/ABT-199	0.40	0.22	0.12
	DEXA/ABT-737	1.8	2.2	2.8
	DEXA/ABT-199	1.44	3.50	10.58
	L-ASP/ABT-737	<0.001	<0.001	<0.001
	L-ASP/ABT-199	0.06	0.05	0.04
	DOX/ABT-737	0.23	0.12	0.06
	DOX/ABT-199	0.12	0.07	0.04
SEMK2	VCR/ABT-737	0.8	0.6	0.4
	VCR/ABT-199	0.8	0.4	0.2
	AraC/ABT-737	>10	>10	0.4
	AraC/ABT-199	>10	1.1	0.02
	DEXA/ABT-737	0.5	0.4	0.3
	DEXA/ABT-199	0.4	0.2	0.1
	L-ASP/ABT-737	0.002	0.004	0.008
	L-ASP/ABT-199	0.003	0.002	0.001
	DOX/ABT-737	0.7	0.6	0.5
	DOX/ABT-199	0.8	0.4	0.2
RS4;11	VCR/ABT-737	1.00	0.84	0.70
	VCR/ABT-199	0.63	0.57	0.52
	AraC/ABT-737	>10	0.35	0.34
	AraC/ABT-199	>10	0.12	0.26
	DEXA/ABT-737	6.23	6.01	5.80
	DEXA/ABT-199	0.32	0.42	0.55

Table S4, Related to Experimental Procedures. List of antibodies used in RPPA

**Antibody List**	**Company**	**cat#**
BAX	Cell Signaling	2772
BCL-2	DAKO	M0887
BIM	Epitomics	1036-1
BCL-X_L	BD biosciences	559027
MCL-1	Cell Signaling	2762S

Figure S1, Related to Figure 1, Association of high BCL-2 protein expression in MLLr with transcript levels of *BCL2.*

Adult ALL: ECOG E2993

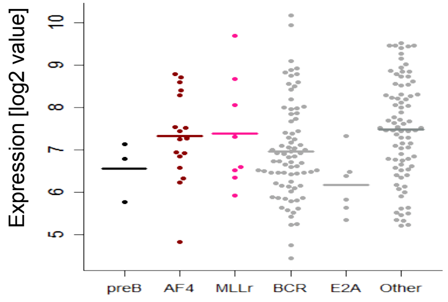

P values

BCL2_ave	BCR	E2A	rMLL	AF4	other rMLL	others	preB
BCR	1	0.0669	0.0958	0.1146	0.4264	0.0036	0.6617
E2A	0.0669	1	**0.0145**	**0.0197**	0.0593	0.0091	0.5476
other rMLL	0.0958	0.0145	1	0.9285	0.8830	0.5147	0.2186
AF4	0.1146	0.0197	0.9285	1	0.8419	0.5746	0.1789
**MLL.8**	0.4264	0.0593	0.8830	0.8419	1	0.6966	0.4970
others	0.0036	0.0091	0.5147	0.5746	0.6966	1	0.1784
preB	0.6617	0.5476	0.2186	0.1789	0.4970	0.1784	1

191 ECOG (E2993):
BCR/ABL1: (78)
E2A/PBX1: (6)
MLL (25) : MLL/AF4 (17) + other MLLr (8)
Other B-ALL: (82)
normal preB: (3)

GEO#: GSE34861

ECOG E2993 clinical trail, n=191
Huimin Geng, et al. Cancer Discovery 2012

Childhood ALL: COG P9906

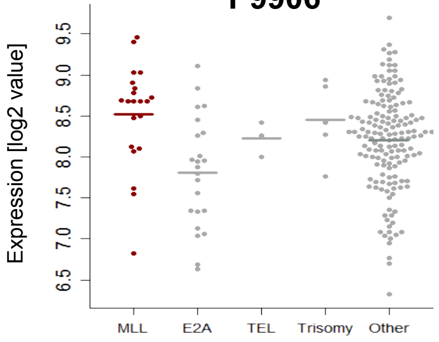

P values

BCL2_ave	E2A	MLLr	TEL	others	Trisomy
E2A	1	**0.0005**	0.2415	0.0062	0.0615
**MLLr**	0.0005	1	0.1719	0.0101	0.8010
TEL	0.2415	0.1719	1	0.9188	0.5714
Trisomy	0.0615	0.8010	0.5714	1	1.0000
Other	0.0062	**0.0101**	0.9188	1.0000	1

COG P9906 clinical trail, n=207
Rechard Harney et al, Blood 2010

207 COG (P9906):
MLLr: (21)
E2A/PBX1: (23)
Other B-ALL: (155)
TEL/AML1: (3)
Trisomy 4 or 10: (5)

GEO#: GSE28460

Childhood ALL: St. Jude ALL

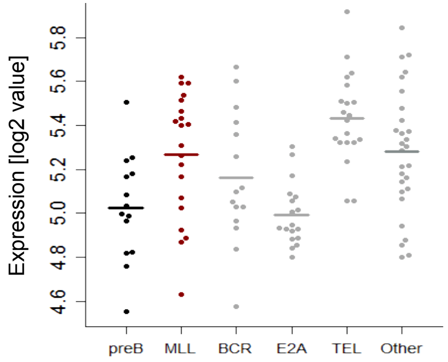

P values

BCL2_ave	BCR	E2A	MLL	TEL	others	preB
BCR	1	0.0437	0.3299	0.0068	0.2193	0.2340
E2A	0.0437	1	**0.0019**	0.0000	0.0003	0.6395
**MLL**	0.3299	0.0019	1	0.1081	0.8766	0.0150
TEL	0.0068	0.0000	0.1081	1	0.0499	0.0000
others	0.2193	0.0003	0.8766	0.0499	1	0.0088
preB	0.2340	0.6395	**0.0150**	0.0000	0.0088	1

St Jude:
BCR: (15)
E2A: (18)
MLL: (20)
TEL: (20)
Hyperdip: (17)
Other B-ALL: (28)
normal preB: (14)

St Jude ALL:http://www.stjuderesearch.org/data/ALL3. (no GEO number, but raw data can be downloaded from the above website). # normal preB (14): http://franklin.et.tudelft.nl/.

Figure S2,Related to Figure 2,MLL/AF4 controls activation of the *BCL-2* gene.

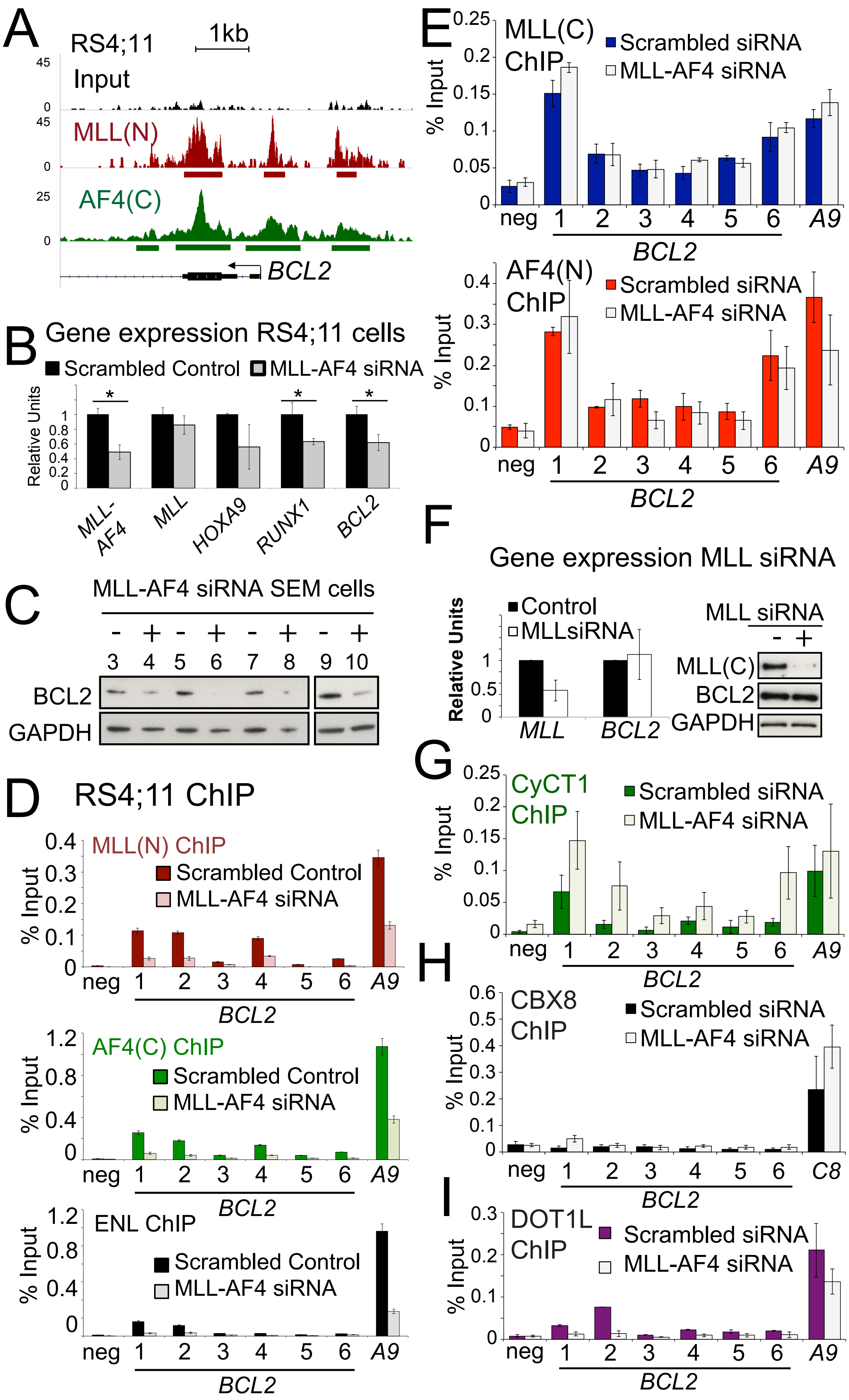

Figure S3, Related to Figure 3, Loss of BCL-2 reduces SEM cell growth and the DOT1L inhibitor EPZ5676 has minimal effect on the survival of Nalm-6 cells.

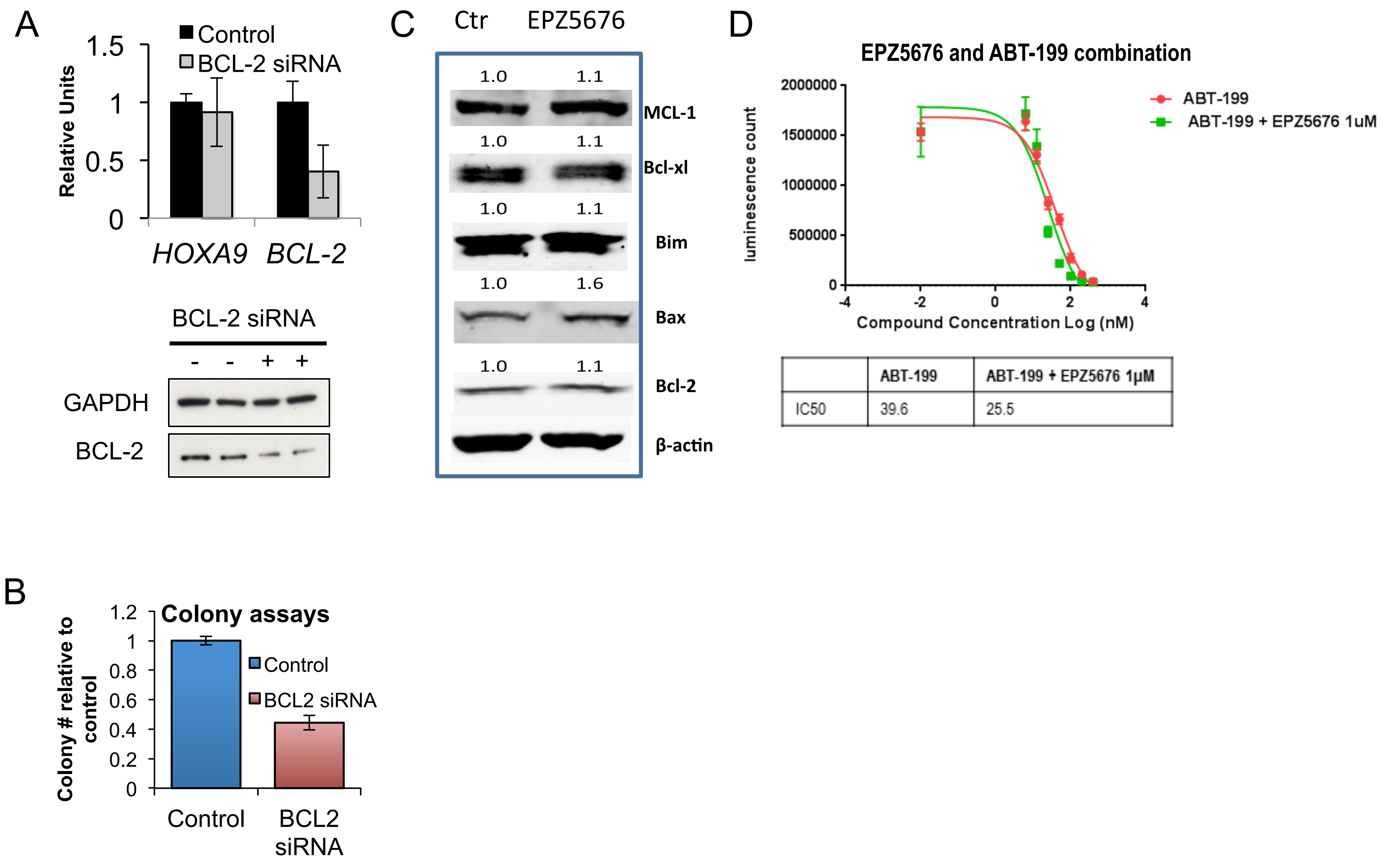

Figure S4, related to Figure 5, ABT-737 or ABT-199 in ALL cell lines and primary samples.

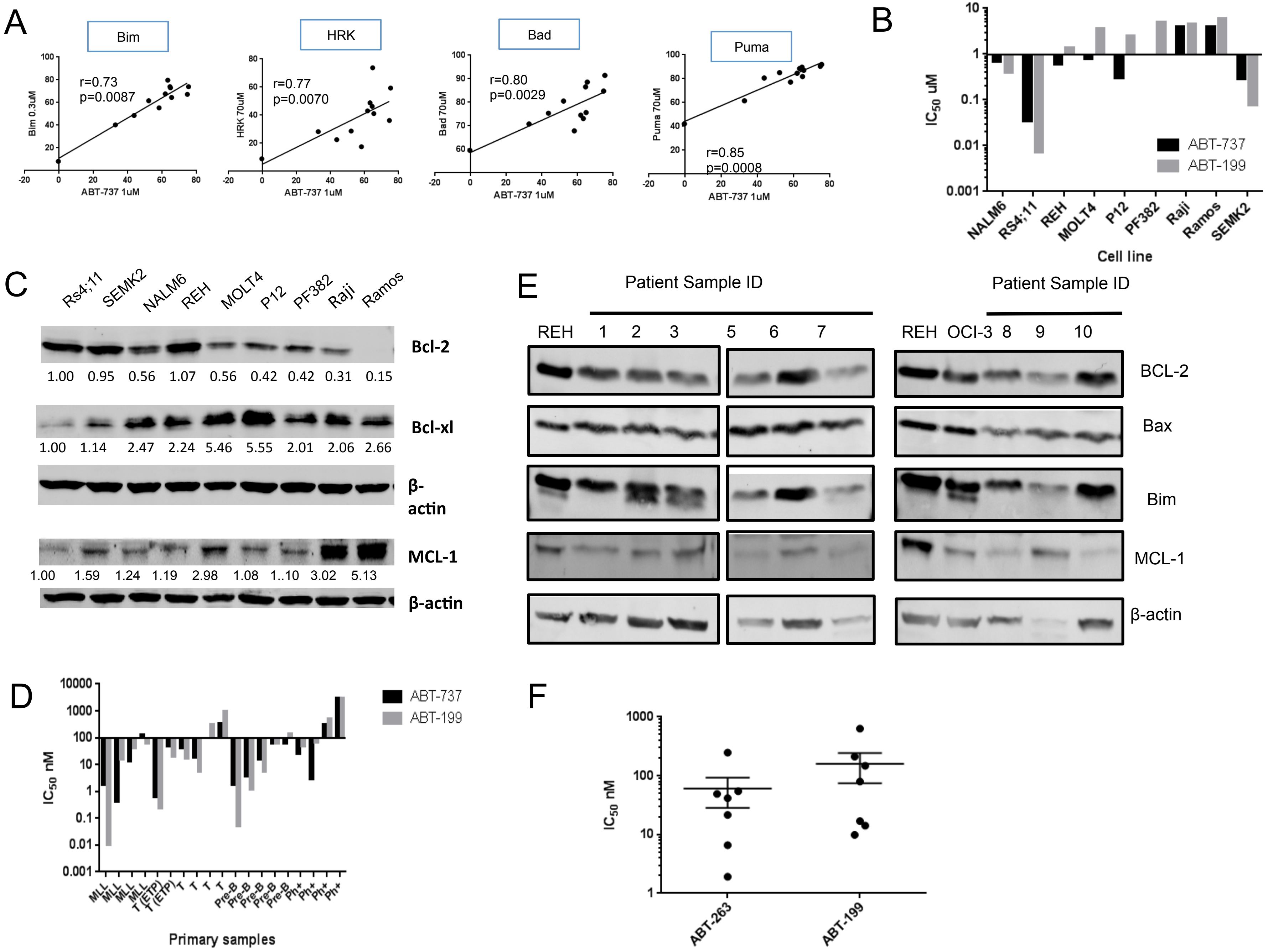

Figure S5, related to Figure 6, FAF1 is a target of MLL/AF4 but does not contribute to leukemic growth.

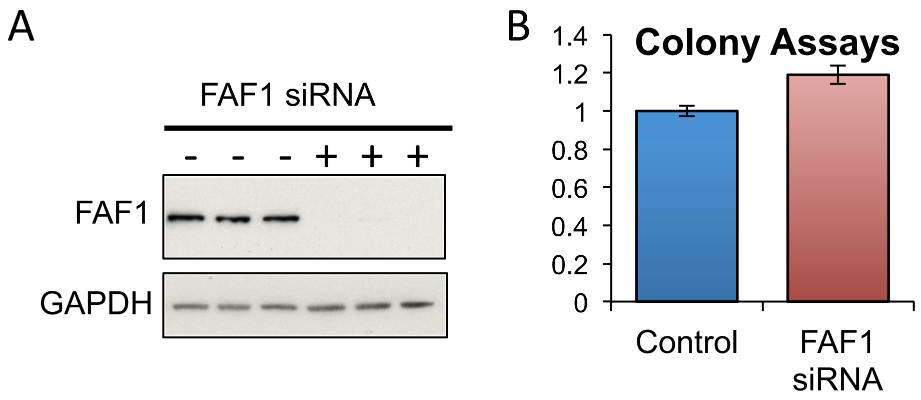

Figure S6, related to Figure 7, ABT199 Inhibits Leukemia Progression in ALL xenograft Model In Vivo.

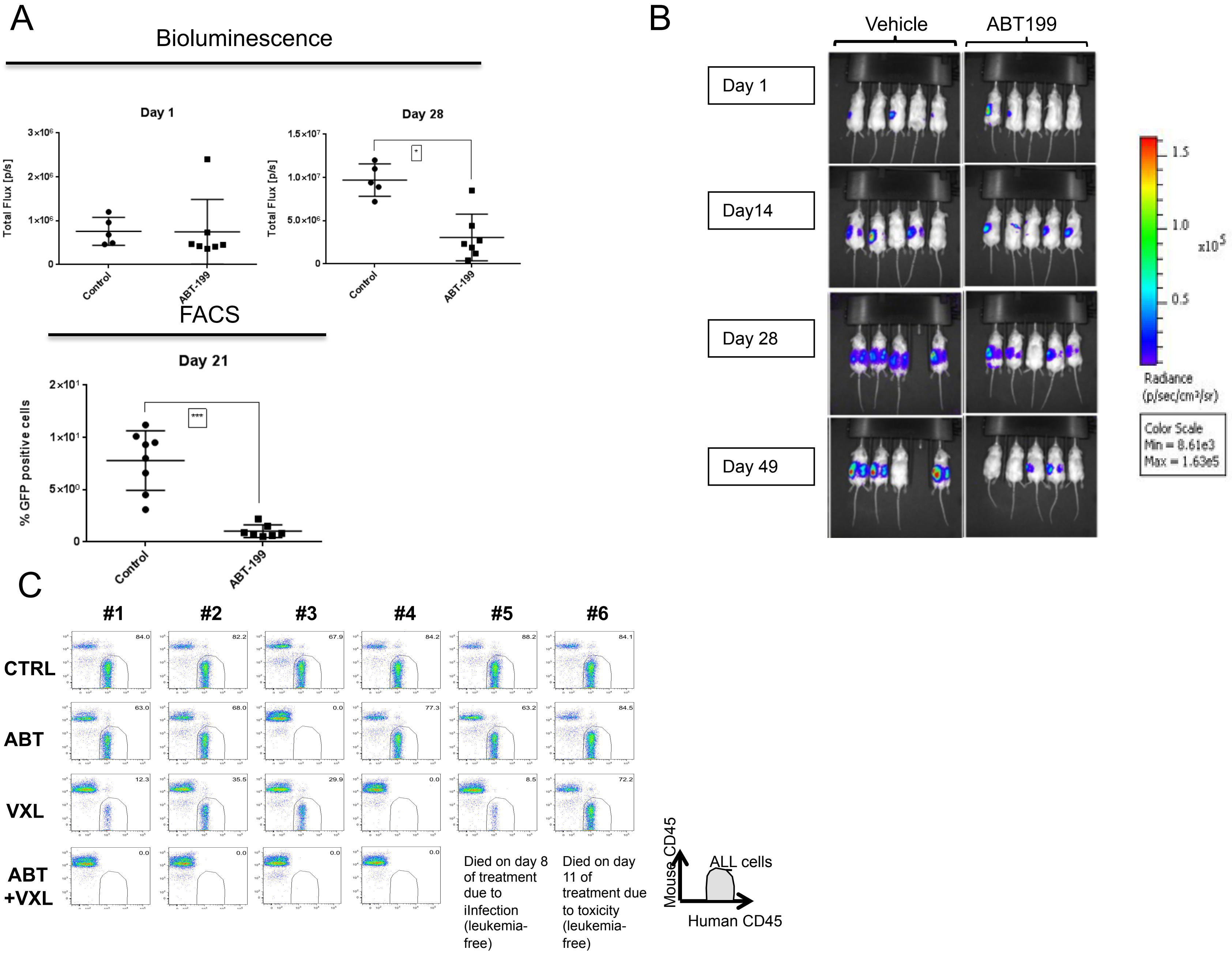

Supplemental Figure Legends.

Figure S1, Related to Figure 1. Association of high BCL-2 protein expression in MLLr with transcript levels of *BCL2. G*ene expression microarray data from 3 large cohorts of patients with ALL were analyzed. A significant increase in *BCL2* mRNA expression in MLLr samples versus normal B-cell controls was detected in the St. Jude cohort ($P = 0.015$). *BCL2* mRNA expression levels were significantly higher in the MLLr samples than E2A/PBX1 samples in all the three studies ($P = 0.0145$, 0.0005 and 0.0019) and higher than molecularly non-designated B-ALL samples in one study ($P = 0.010$).

Figure S2, Related to Figure 2. MLL/AF4 controls activation of the *BCL-2* gene. (A) ChIP-sequencing (ChIP-seq) for MLL-N (red) and AF4-C (green) in RS4;11 cells at the *BCL-2* promoter and first exon. (B) Real time PCR of MLL-AF4, wild type MLL, *HOXA9*, *RUNX1* and *BCL-2* expression in RS4;11 cells treated with either a control (black bars) or an MLL-AF4 specific siRNA (gray bars). In each case, signal was normalized to control treated cells and is the average of three independent knockdown experiments. Error bars represent the standard deviation and * indicates at least $p < 0.02$ (C) Western blots for the indicated proteins in SEM cells treated with either a control (-) or MLL-AF4 specific (+) siRNA. Four biological replicate pairs are shown. (D) ChIP experiments for MLL(N), AF4(C) and ENL in RS4;11 cells treated with either control (dark colored bars) or MLL-AF4 siRNAs (light colored bars). PCR primers are as indicated in Figure 2. Values represent the average of 2 independent knockdown experiments and error bars represent the standard error of the mean. (E) ChIP experiments for MLL(C) and AF4(N) and ENL in SEM cells treated with either control (dark colored bars) or MLL-AF4 siRNAs (light colored bars). Values represent the average of three independent knockdown experiments and error bars represent the standard error of the mean. (F) Real time PCR (left panel) and western blots (right panel) of wild type MLL and BCL-2 normalized to GAPDH in SEM cells treated with either a control (black bars) or an MLL specific siRNA (white bars). (G-I) ChIP experiments for Cyclin T1, CBX8 and DOT1L in SEM cells treated with either control (dark colored bars) or MLL-AF4 siRNAs (light colored bars). PCR primers are as indicated in Figure 2. Values represent the average of 4 (CycT1, CBX8) or 2 (DOT1L) independent knockdown experiments and error bars represent the standard error of the mean.

Figure S3, Related to Figure 3. Loss of BCL-2 reduces SEM cell growth and the DOT1L inhibitor EPZ5676 has minimal effect on the survival of Nalm-6 cells. (A) RT-PCR and western blots of SEM cells treated with BCL-2 siRNA's. (B) Colony assays of the SEM treated samples from C representing the average of two separate knockdowns. Error bars = sd. (C) Western blot analysis of SEMK2 cells treated with EPZ5676 1μM for 7 days. The numbers indicate the relative density of the bands relative to untreated control. (D) Nalm-6 cells were treated with increasing concentrations of ABT-199 and 1uM EPZ5676 and growth-inhibitory effects determined using the Cell viability luminescence assay (CellTiter-Glo®, Promega). The effective dose for 50% cell killing (IC50) was determined using Graph Pad Prism 5.0.

Figure S4, Related to Figure 5. ABT-737 or ABT-199 in ALL cell lines and primary samples. (A) Correlation between percentage (%) of mitochondrial depolarization in B-ALL cells induced by the indicated BH3 peptides and ABT-737 (Sample #18-29 in Supplementary Table 1). R values results from Spearman analysis of data (B and D). IC50 for ABT-737 (black bars) or ABT-199 (grey bars) in ALL cell lines (B) and primary samples (D). Values were determined based on the number of live cells remaining after treatment for 48 h. Among ALL cell lines, RS4;11 and SEM-K2 are t(4;11)-positive; P12-ICHIKAWA, MOLT-4, and PF382 are T-cell lines; Ramos and Raji are Burkitt cell lines. (C and E) Expression of BCL-2, BCL-X_L and MCL-1 in ALL cell lines and primary samples determined by WB analysis. Values indicate protein quantification relative to β-actin. (F) Sensitivity of pediatric B-ALL samples to ABT-199 and ABT-263. IC_{50} values of primary B-cell ALL samples is graphed following short term ex-vivo culture with ABT-199 and ABT-263 for 8hr. Values were determined based on the number of live cells remaining after treatment for 8 h. For clinical information, please refer to Table S1 (Samples #23-29).

Figure S5, related to Figure 6. FAF1 does not contribute to leukemic growth. (A) FAF1 siRNA knockdowns in SEM cells reduce FAF1 protein levels (western blot). (B) FAF1 siRNA knockdowns have no effect on SEM cell growth (colony assay).

Figure S6, related to Figure 7. ABT199 Inhibits Leukemia Progression in ALL xenograft Model In Vivo. (A) ALL-236-GFP/Luciferase cells generated from pre-B-ALL with t(4;11) were injected intravenously into the recipient NSG mice. On day 35 post-injection mice were randomized (N=5/group, Day =1) and treated with vehicle

or ABT199 (100mg/kg/day) for 10 days. Bioluminescence quantification performed at the treatment start (day 1) and on day 28 after treatment onset is shown. Leukemia burden was confirmed by detection of circulating GFP + cells on day 21. (B) BLI of NSG mice engrafted with ALL-236-GFP and treated with ABT-199 for 10 days. Time refers days relative to treatment onset. (C) Selective inhibition of BCL-2 by ABT-199 and induction type chemotherapy synergistically eradicate patient derived ALL cells in vivo. Leukemia cells from a patient with t(4;11) ALL (#682) were injected to NRG mice via tail vein. On day 24 post engraftment, mice were randomly divided into cohorts to receive VXL (Vincristine, Dexamethasone, L-asparaginase) or ABT-199 alone or in combination, or vehicle controls (n = 6/arm). Time course changes in percentages of circulating ALL cells in case #682. Leukemia progression was evaluated by determining the percentage of circulating human CD45-positive cells across different treatment groups.

Supplemental Experimental Procedures

Cell lines, primary samples, and cultures

Raji, RS4;11, Ramos, REH, NALM-6, and OP-9 cells were purchased from American Type Culture Collection (Manassas, VA). MOLT-4, P12-ICHIKAWA, and PF382 were kindly provided by Dr. Adolfo Ferrando (Columbia University, New York, NY) and SEM-K2 by Dr. Carolyn Felix (University of Pennsylvania, Philadelphia, PA). MLLr ICN3 cells were provided by Dr. Markus Muschen (UCSF, San Francisco, CA).(Duy et al., 2011) SEM cells(Greil et al., 1994) were purchased from DSMZ (www.cell-lines.de) and cultured in IMDM (Gibco) supplemented with 15% FCS. Cell lines were validated by the MD Anderson Cancer Center Cell Line Validation Core Facility. RS4;11, REH, NALM-6, and SEM-K2 represent precursor B-cell ALL, Raji and Ramos mature B-cell ALL, and MOLT-4, P12-ICHIKAWA, and PF382 T-cell ALL. RS4;11, SEM and SEM-K2 carry t(4;11), SEM-K2 is a subclone of SEM with identical features.(Zweidler-McKay et al., 2005) Except for SEM, cell lines were maintained in RPMI 1640 medium containing 10% heat-inactivated FBS. For treatment studies, REH, SEMK2 and RS4;11 were treated with ABT-737 or ABT-199 in combination with vincristine (VCR), doxorubicin (DOX), cytarabine (AraC), dexamethasone (DEXA), or L-asparaginase (L-ASP) for 48 h (REH, SEMK2) or 24 h (RS4;11). For Figure 3H and S3C SEMK2 cells were treated with ABT-199 in combination with DOT1L inhibitors SGC0946 (Sigma) or EPZ5676 (BioVision, Inc.) for 4 or 7 days, and effects on cell viability determined using the Cell viability luminescence assay (CellTiter-Glo®, Promega). The effective dose for 50% cell killing (IC50) was determined using Graph Pad Prism 5.0. For treatment of primary samples, patient mononuclear cells were plated on a monolayer of OP-9 stromal cells in RPMI 1640 medium containing 10% FBS and supplemented with the following recombinant human cytokines (all from Gemini Bio-Products): TPO, FLT3 ligand, and IL-3 (10 ng/mL); IL-6 (20 ng/mL); and SCF (100 ng/mL). Cells were treated with ABT-737, ABT-199, a chemotherapy agent, or a combination of ABT-199 and a chemotherapy agent for 24 h. For assessment of pediatric and adult ALL samples from Dana-Farber Cancer hospital primary patient mononuclear cells were grown in RPMI 1640 with 10% FBS and supplemented with IL-3 (10 ng/mL); IL-6 (20 ng/mL); and SCF (100 ng/mL). Cells were treated with ABT-199 and ABT-263 for 8hr prior to measuring cell viability.

Cell viability, apoptosis analysis and BH3 profiling

Viable cells were enumerated by flow cytometry using counting beads (Invitrogen, Carlsbad, CA) with concurrent Annexin-V and DAPI or 7-aminoactinomycin D (7-AAD) staining. IC_{50} values for ABT-199 or ABT-737 were calculated from numbers of live (Annexin V-negative/DAPI or 7AAD-negative) cells after treatment (at 48 h for cell lines or 24 h for primary samples) by using Calcusyn software (Biosoft, Ferguson, MO). Calcusyn was utilized to determine combination index values based on the percentage of cell death induced by each treatment.

For intracellular BH3 profiling of primary ALL cells, thawed primary ALL cells were washed once with PBS and stained with 1:100 Invitrogen Live/Dead – Aqua stain (#34957, Life Technologies) in FACS buffer (2% FBS in PBS, 1:100) for 20 min on ice, washed with FACS buffer, and subsequently stained with CD45-V450 (#642275; BD Biosciences; 1:100) in FACS buffer on ice for 20 min. ALL primary cells were identified as CD45 mid, SSC-A low. Intracellular BH3 profiling was performed as described in Pan et al (Pan et al., 2014).

siRNA experiments

Briefly, using a rectangle pulse EPI 2500 electroporator (Fischer, Heidelberg), 7 X 10^7 SEM or RS4;11 cells were subjected to a 10msec 350V (SEM) or 370V (RS4;11) electroporation in the presence of 300 pmol siRNA. MLL-AF4 siRNA sequences were obtained from(Thomas et al., 2005) and are the following: siMA6 (sense, AAGAAAAGCAGACCUACUCCA;antisense, UGGAGUAGGUCUGCUUUUCUUUU), targeting the MLL exon 9 and AF4 exon 4 MLL-AF4 fusion site present in SEM cells, and siMARS (sense, ACUUUAAGCAGACCUACUCCA-; antisense, UGGAGUAGGUCUGCUUAAAGUCC-), targeting the exon 10–exon 4 fusion site variant present in RS4;11 cells. As control siRNAs we used the mismatch control siMM (sense, AAAAGCUGACCUUCUCCAAUG; antisense, CAUUGGAGAAGGUCAGCUUUUCU). Wild type MLL siRNAs (Dharmacon on Target Plus Smartpool, L-009914-00), FAF1 siRNAs (Dharmacon on Target Plus Smartpool, L-009106-00) and BCL-2 siRNAs (Dharmacon on Target Plus Smartpool, L-003307-00) were all compared to a non-targeting smartpool control (Dharmacon On Target plus non targeting pool D001801020).

Colony forming assays

24 hours post second transfection cells were plated at a density of 1, 2 or 2.5 x 10^5 cells per ml, in triplicate, plated in IMDM MethoCult media (H4100; STEMCELL Technologies) supplemented with FCS and cultured for 14 days (37 °C, 5% CO_2) before counting. Colony forming assays were run in triplicate with at two-three biological repeats.

Western blot analysis

The following mAb were used: mouse anti-BCL-2 (Dako Cytomation, Carpinteria, CA); rabbit anti-BCL-X_L, BAX, BIM, FAF1 (Cell Signaling Technology, Beverly, MA); mouse MCL-1 (BD Pharmingen, San Diego, CA); and mouse anti-β-actin (Sigma-Aldrich). An Odyssey Infrared Imaging System and Odyssey software v2.0 (LI-COR Biosciences, Lincoln, NE) were used to scan the blots and quantify band intensities, respectively. The ratio of band intensity of each BCL-2 family protein relative to that of loading control was normalized to the ratio in untreated NALM-6 cells. For Figures 2, 3, 4, S2 and S5 the following antibodies were used for western blotting: a-AF4-C (For both MLL/AF4 and wild type AF4, Abcam, ab31812); a-MLL-C (Active Motif, 61295); a-ENL (Bethyl, A302-268A); a-BCL-2 (Cell Signaling, 2870); a- BCL-X_L (Cell Signaling, 2764); a-MCL-1 (Cell Signaling, 5453); a-BIM (Abcam, ab32158); a-BAX (Cell Signaling,2772); a-GAPDH (Bethyl, A300-641A); a-H3 (Abcam, ab1791); a-RUNX1 (Cell Signaling, 4334); a-H3K79me2 (Active Motif 39143); a-H3K79me3 (Diagenode pAb-068-050), a-FAF1 (Bethyl, A302-810A).

Bimolecular fluorescence complementation

The coding sequences for human BCL-2, BCL-X_L, MCL-1, BIM, and NOXA were subcloned by standard PCR strategies into BiFC plasmids containing Venus fragments (FLAG/VN173 or HA/VC155; VN and VC are the N- and C-terminal fragments of the Venus protein, respectively). HeLa cells were grown to at least 50% of confluence in 24-well plates. One hour before transfection, 2.5 μM of ABT-199 or vehicle (DMSO) was added. Cells then were transfected with a pBiFC vector (0.3 μg/10^5 cells) containing the cDNA for a VN-BCL-2/BCL-X_L/MCL-1 fusion and a pBiFC vector expressing a VC-BIM fusion. Lipofectamine 2000 (Invitrogen) was used for transfection according to the manufacturer's instructions. Cells were co-transfected with an equal amount of the pAL2-mRFP vector containing *mRFP* cDNA to assess transfection efficiency and relative fluorescence intensities. Z-VAD-fmk (50 μM) was added to delay cell death and preserve cell morphology. After transfection, cells were cultured for 24 h and subjected to trypsinization; Venus and mRFP signals were quantified in a FACSCalibur flow cytometer using 488-nm and 635-nm excitation lasers, respectively. A gating analysis based on mRFP fluorescence was performed to exclude non-transfected cells. The mean fluorescence intensities of the BiFC complexes were normalized to the mean fluorescence intensity of mRFP. At least 10,000 cells were analyzed in each experiment. Results are expressed as the fold-change induced by ABT-737 or ABT-199 in the Venus/RFP intensity ratio for each protein pair. Results are the mean ± SD of two independent experiments, with duplicates (n=4).

Chromatin immunoprecipitation assays

For RS4;11 ChIP-seq, fixed chromatin samples were fragmented by a Bioruptor sonicator (Diagenode, Denville, NJ) for 20 min at high in a constantly circulating 4°C water bath to an average size of 200-500 bp. For all other ChIP and ChIP-seq, samples of up to 10^8 cells were sonicated on a Covaris (Woburn, MA) according to the manufacturers' recommendations. Ab:chromatin complexes were collected with a mixture of Protein A and Protein G Dynabeads (Life Technologies, Grand Island, NY) by using a magnet and were then washed twice with a solution of 50mM Hepes-KOH, pH 7.6, 500mM LiCl, 1mM EDTA, 1% NP-40, and 0.7% Na-deoxycholate. After a Tris-EDTA wash, samples were eluted, treated with RNAse and proteinase K, and purified by using a Qiagen PCR purification kit. ChIP samples were quantified relative to inputs (Milne et al., 2009). Briefly, the amount of genomic DNA co-precipitated with antibody was calculated as a percentage of total input using the following formula: $\Delta C_T = C_T$ (input) − C_T (ChIP), total percentage = $2^{\Delta CT} \times 5.0\%$. A 50-μL aliquot taken from each of 1 mL of sonicated, diluted chromatin before Ab incubation served as the input, and thus the signal from the input samples represents 5% of the total chromatin used in each ChIP. Histone modification % input ChIP signal was normalized to H3 % input ChIP signal. C_T values were determined by choosing threshold values in the linear range of each PCR reaction.

ChIP sequencing.

ChIP samples were submitted to the Wellcome Trust Centre for Human Genetics for library preparation(Lamble et al., 2013) and sequencing. Samples were sequenced using a HiSeq 2500 and 50bp paired-end sequencing. Data were mapped to the Homo sapiens hg18 genome using Bowtie. Conversion to bam files was done using samtools. Duplicate reads were removed and data was normalized to an input track, in SeqMonk. Peaks were called using the probe generator in SeqMonk. The data discussed in this publication have been deposited in NCBI's Gene Expression Omnibus (Edgar et al., 2002) and are accessible through GEO Series accession number GSE 74812.

(http://www.ncbi.nlm.nih.gov/geo/query/acc.cgi?acc=GSE74812).

Antibodies used for ChIP and ChIP-seq assays

a-MLL-N (Bethyl, A300-086A, ChIP and ChIP-seq); a-AF4-C (Abcam, ab31812, ChIP, ChIP-seq and western blot); a-AF4-N (Bethyl, A302-344A, ChIP); a-ENL (Bethyl, A302-268A, ChIP and ChIP-seq); a-AF9 (Bethyl, A300-595A, ChIP); a-CDK9 (Bethyl, A303-493A, ChIP); a-CyclinT1 (Bethyl, A303-496A, ChIP); a-AFF4 (Bethyl, A302-538A, ChIP); a-MLL-C (Active Motif, 61295, ChIP); a-CFP1 (Bethyl A303-161A); a-DOT1L (Bethyl, A300-953A, ChIP); a-H3K79me2 (Millipore 04-835, ChIP-seq; Active Motif 39143 ChIP) a-H3K79me3 (Diagenode pAb-068-050, ChIP and ChIP-seq) ; a-H3 (Abcam, ab1791); a-H3K4me3 (Diagenode, C1541003 ChIP and ChIP-seq); a-H3K27Ac (Diagenode, C15410196, ChIP and ChIP-seq); a-H3K4me1 (Diagenode, C15410194, ChIP-seq); a-CBX8 (Bethyl, A300-882A).

PCR primers for RT-PCR

The following Taqman primer-probe sets were purchased from Applied Biosytems (Waltham, MA, USA): b2M Hs99999007; GAPDH Hs03929097_g1; BCL-2 Hs00608023_m1; BAX Hs00180269_m1; MCL-1 Hs01050896_m1; BCL2L1 Hs00236329_m1; BCL2L11 Hs00708019; MLL Hs00172962_m1; ENL Hs00172962_m1; RUNX1 Hs00231079_m1. HOXA9 Taqman primer/probe set were as follows: HOXA9primer For: AAAACAATGCCGAGAATGAGAGCG, HOXA9 FAM/TAMRA probe: CCCCATCGATCCCAATAACCCAGC, HOXA9primer Rev: TGGTGTTTTGTATAGGGGGACC; MLL/AF4 SYBR green primers for both SEM and RS4;11 cells are as follows: MLL/AF4 For: AGGTCCAGAGCAGAGCAAAC; MLL/AF4 Rev: CGGCCATGAATGGGTCATTTC, GAPDH SYBR green primers are as follows: For: AACAGCGACACCCATCCTC; Rev CATACCAGGAAATGAGCTTGACAA.

PCR primers for ChIP

Negative control region For: GGCTCCTGTAACCAACCACTACC, Negative control region Rev: CCTCTGGGCTGGCTTCATTC; HOXA9 For: ATGCTTGTGGTTCTCCTCCAGTTG, HOXA9 Rev: CCGCCGCTCTCATTCTCAGC; HOXC8 For: AGACTTCTTCCACCACGGCAC, HOXC8 Rev: TAAGCGAGCACGGGTTCTGC; BCL2-1For: GAGGAGGGCTCTTTCTTTCTTC, BCL2-1Rev: GCCTGTCCTCTTACTTCATTCTC; BCL2-2For: CGATAACGCCTGCCATCTAA, BCL2-2Rev: CCACCACATCCTACTGGATTAC; BCL-2-3 primers are from,(Dawson et al., 2011) BCL2-3For: AGCCCCTGGAGAAGTATGGT, BCL2-3Rev: CATCCGTTAGCATGAAGCAA; BCL2-4For: GGCCAGGGTCAGAGTTAAATAG, BCL2-4Rev: GGAGGTTCTCAGATGTTCTTCTC; BCL2-5For: GGAAACCCAGACCAACTCAT, BCL2-5Rev: CCTTATCTCAGGAGGACGTAGA; BCL2-6For: GAGCCCTCAACCTTGTGATAG, BCL2-6Rev: AAGGTAGCCCTGACCATAGA; BAX-10For: CGTGGGCTATATTGCTAGATCC, BAX-10Rev: CTTCCAGGCAGGACGTTATAG; BAX-11For: TTTGCACTTGCTAATTCCTTCTG, BAX-11Rev: GCAGCTCTAATGCCTTCATTTATC; BIM-8For: CTCTTGGCAGAGACAGAAAGG, BIM-8Rev: CAGAGAACGCAGTGTGAGAA; BIM-9For: GAGGAAGTTGTTGGAGGAGAATAG, BIM-9Rev: CTCGCCACTTGTCCTTGTT; MCL1-4For: CCAAACATTGGACTGAGAGTAGAG, MCL1-4Rev: GTTCAGTGATGGATGGGAACA; MCL1-5For: TTCCGCCCATCTTGATTCTT, MCL1-5Rev: TCGCTACTGGGATTTACAGAAC; MCL1-6For: CCTGAGTTCTGTAAATCCCAGTAG, MCL1-6Rev: CGAAGCATGCCTGAGAAAGA; MCL1-7For: GGCATCTTTGGATTTCAGTCTTG, MCL1-7Rev: CTGTAGAGGGAGCAGAACAATC; BCLXL-12For: GGAAGGCATTTCGGAGAAGA, BCLXL-12Rev: TCTGGGTCTAGGTTCCAAGATA; BCLXL-13For: GAGCTGGTGGTTGACTTTCT, BCLXL-13Rev: CAGTCCTGTTCTCTTCCACATC; BCLXL-14For: GCTTCAGAGATCAGGCATCTT, BCLXL-14Rev: CCTCACAGGTTTGGGACTTAAT; BCLXL-15For: CAGTGTAGGCTGTGCAGATT, BCLXL-15Rev: GCAAGTGCTCCACAAACAAG.

Patient microarray gene expression data

Microarray gene expression data from three large cohorts of patients with ALL were analyzed. These cohorts included the Eastern Cooperative Oncology Group (ECOG) Clinical Trial E2993 (GEO#: GSE34861) cohort: 191 total samples comprising 78 BCR-ABL1 patients, 6 E2A-PBX1 patients, 25 MLLr patients (t(4;11): 17, other MLLr: 8), and 82 other B-ALL patients;(Geng et al., 2012) the Children's Oncology Group (COG) Clinical Trial P9906 (GEO#: GSE28460) cohort: 207 total samples, 23 E2A-PBX1 patients, 21 MLLr patients, 3 RUNX1-ETV6 patients, 155 other B-ALL patients (trisomy 4 or 10 patients);(Harvey et al., 2010) and the St. Jude Research Hospital pediatric ALL clinical trial cohort: 132 total samples, 15 BCR-ABL1 patients, 18 E2A-PBX1 patients, 20 MLLr patients, 20 RUNX1-ETV6 patients, 17 hyperdiploid patients, 28 other B-ALL patients, 14 T-ALL patients

(Ross et al., 2003). This last cohort has no GEO number, but raw data can be downloaded from the following site: http://www.stjuderesearch.org/site/data/ALL3/.

The microarray raw data were normalized by using the Robust Multi-array Average (RMA) method(Bolstad et al., 2003) with Expression Console software (Version 1.1, Affymetrix) for the Affymetrix arrays HG-U133 plus2 (COG data) or HG-U133 A and B (St. Jude data), or with NimbleScan software (version 2.5, Roche NimbleGen, Madison, WI) for the NimbleGen array HG18 60mer expression 385K platform (ECOG data). The patients in each cohort were grouped into subtypes according to their cytogenetic features: BCR-ABL, E2A-PBX1, MLLr, ETV6-RUNX1, or other ALLs, which were negative for these translocations. T-ALL samples were excluded from this analysis. MLL fusion partner information was available for the ECOG MLLr ALL data, which were therefore further separated into MLL/AF4 (n=17) or other MLLr (n=8). No MLL fusion partner information was available for the COG or St. Jude clinical trials, so MLLr ALL patients were treated as one group. Expression level of a gene in a sample was determined by the average of expression values from multiple probe sets on the array representing this gene. The *P*-values of differential expression of *BCL2* between MLLr and other ALL subtypes were determined by two-sided Wilcoxon test. All downstream microarray analysis was performed by using R version 2.14.0 (R Development Core Team. R: A Language and Environment for Statistical Computing. 2009; http://www.R-project.org).

RPPA statistical analysis

Expression of pro- and anti-apoptotic BCL-2 family proteins (BAX, BCL-2, MCL-1, BCL-X_L, and BIM) was studied in 186 newly diagnosed ALL. Associations between RPPA protein expression levels and categorical clinical variables were assessed in R using a standard *t*-test, linear regression, or mixed-effects linear model. Associations between continuous variable and protein levels were assessed by using Pearson and Spearman correlations and linear regression. Bonferroni corrections were performed to account for multiple statistical parameters for calculating statistical significance. RPPA antibody information is provided in Table S4.

***In vivo* murine leukemia models**

Three *in vivo* murine leukemia models were used. In ONE model, 10^6 ALL-236-GFP/LUC cells generated from pre-B-ALL with t(4;11) as described elsewhere (Terziyska et al., 2012) were injected intravenously into NOD SCID/IL2Rγ-KO (NSG) mice. On day 21 after injection, peripheral blood samples were collected by retro-orbital bleeding and the presence of GFP-positive cells determined by FACS. Engraftment was detected on day 32 after cell injection by bioluminescence imaging (BLI; IVIS-200, Xenogen, Cranbury, NJ) following administration of the LUC substrate coelenterazine (native; Biotium, Heyward, CA). Three days later, the mice began daily treatment with vehicle (Phosal 50PG/PEG 40/ethanol, 60/30/10 v/v) or ABT-199 (100 mg/kg per day) by oral gavage for 10 days (5 days/week with a drug-free weekend holiday). Antitumor activity was tested by BLI 2 and 4 weeks after initiation of treatment.

For the second model, 12 NSG mice were injected intravenously with ICN3 xenograft cells (2.5×10^5 cells/mouse) generated from a child with relapsed MLLr pre-B-ALL (Duy et al., 2011). Leukemia engraftment was established by detection of circulating human CD19-positive cells in peripheral blood. At day 45 after injection, mice were randomized into two treatment groups (n=6/group) for treatment with vehicle only or ABT-199 (100 mg/kg per day). Mice were treated by oral gavage for 5 consecutive days, given a drug-free weekend holiday and then were treated for 2 more days. Peripheral blood was collected on days 4 and 10 after treatment initiation. Mice were euthanized the morning after the last drug dose. BM, spleen, and peripheral blood were analyzed for leukemia burden by CD19 cytometry.

For the third model, we combined ABT-199 and an induction-type regimen consisting of VCR, L-ASP, and DEXA (VXL).(Szymanska et al., 2012) NOD.Cg-Rag1^{tm1Mom} IL2rg^{tm1Wjl}/SzJ (NRG) mice were obtained from Jackson Laboratory (Bar Harbor, ME) and were bred by standard procedures in accordance with a protocol approved by the IACUC. NRG mice were preconditioned with a single i.p. dose of busulfan (40 mg/kg) 24 h prior to tail vein injection of cryopreserved human patient xenograft pre-B-ALL cells with t(4;11), precoated with OKT3 Ab, as described previously.(Wunderlich et al., 2014) After engraftment of human CD45-positive ALL cells to peripheral blood was observed, mice were randomly divided into cohorts to receive treatment with VCR (0.15 mg/kg in PBS, i.p., 2 doses, days 1 and 8), DEXA (5 mg/kg in PBS, i.p., 7 doses, days 1-5, 8-9), and L-ASP (1000 IU/kg in PBS, i.p., 7 doses, days 1-5, 8-9); ABT-199 (100 mg/kg in 10% ethanol/30% PEG400/60% Phosal50 PG, by oral gavage,

8 doses, days 1-5, 8-9, 11); a combination of the two; or vehicle alone (controls). B-ALL grafts were measured as the percentage of human CD45+CD19+CD33- cells in the peripheral blood by flow cytometry.

Supplemental References

Bolstad, B.M., Irizarry, R.A., Astrand, M., and Speed, T.P. (2003). A comparison of normalization methods for high density oligonucleotide array data based on variance and bias. Bioinformatics *19*, 185-193.

Dawson, M.A., Prinjha, R.K., Dittmann, A., Giotopoulos, G., Bantscheff, M., Chan, W.I., Robson, S.C., Chung, C.W., Hopf, C., Savitski, M.M.*, et al.* (2011). Inhibition of BET recruitment to chromatin as an effective treatment for MLL-fusion leukaemia. Nature *478*, 529-533.

Duy, C., Hurtz, C., Shojaee, S., Cerchietti, L., Geng, H., Swaminathan, S., Klemm, L., Kweon, S.M., Nahar, R., Braig, M.*, et al.* (2011). BCL6 enables Ph+ acute lymphoblastic leukaemia cells to survive BCR-ABL1 kinase inhibition. Nature *473*, 384-388.

Edgar, R., Domrachev, M., and Lash, A.E. (2002). Gene Expression Omnibus: NCBI gene expression and hybridization array data repository. Nucleic Acids Res *30*, 207-210.

Geng, H., Brennan, S., Milne, T.A., Chen, W.Y., Li, Y., Hurtz, C., Kweon, S.M., Zickl, L., Shojaee, S., Neuberg, D.*, et al.* (2012). Integrative epigenomic analysis identifies biomarkers and therapeutic targets in adult B-acute lymphoblastic leukemia. Cancer Discov *2*, 1004-1023.

Greil, J., Gramatzki, M., Burger, R., Marschalek, R., Peltner, M., Trautmann, U., Hansen-Hagge, T.E., Bartram, C.R., Fey, G.H., Stehr, K.*, et al.* (1994). The acute lymphoblastic leukaemia cell line SEM with t(4;11) chromosomal rearrangement is biphenotypic and responsive to interleukin-7. Br J Haematol *86*, 275-283.

Harvey, R.C., Mullighan, C.G., Wang, X., Dobbin, K.K., Davidson, G.S., Bedrick, E.J., Chen, I.M., Atlas, S.R., Kang, H., Ar, K.*, et al.* (2010). Identification of novel cluster groups in pediatric high-risk B-precursor acute lymphoblastic leukemia with gene expression profiling: correlation with genome-wide DNA copy number alterations, clinical characteristics, and outcome. Blood *116*, 4874-4884.

Lamble, S., Batty, E., Attar, M., Buck, D., Bowden, R., Lunter, G., Crook, D., El-Fahmawi, B., and Piazza, P. (2013). Improved workflows for high throughput library preparation using the transposome-based Nextera system. BMC biotechnology *13*, 104.

Milne, T.A., Zhao, K., and Hess, J.L. (2009). Chromatin immunoprecipitation (ChIP) for analysis of histone modifications and chromatin-associated proteins. Methods Mol Biol *538*, 409-423.

Pan, R., Hogdal, L.J., Benito, J.M., Bucci, D., Han, L., Borthakur, G., Cortes, J., DeAngelo, D.J., Debose, L., Mu, H.*, et al.* (2014). Selective BCL-2 inhibition by ABT-199 causes on-target cell death in acute myeloid leukemia. Cancer Discov *4*, 362-375.

Ross, M.E., Zhou, X., Song, G., Shurtleff, S.A., Girtman, K., Williams, W.K., Liu, H.C., Mahfouz, R., Raimondi, S.C., Lenny, N.*, et al.* (2003). Classification of pediatric acute lymphoblastic leukemia by gene expression profiling. Blood *102*, 2951-2959.

Szymanska, B., Wilczynska-Kalak, U., Kang, M.H., Liem, N.L., Carol, H., Boehm, I., Groepper, D., Reynolds, C.P., Stewart, C.F., and Lock, R.B. (2012). Pharmacokinetic modeling of an induction regimen for in vivo combined testing of novel drugs against pediatric acute lymphoblastic leukemia xenografts. PLoS One *7*, e33894.

Terziyska, N., Castro Alves, C., Groiss, V., Schneider, K., Farkasova, K., Ogris, M., Wagner, E., Ehrhardt, H., Brentjens, R.J., zur Stadt, U.*, et al.* (2012). In vivo imaging enables high resolution preclinical trials on patients' leukemia cells growing in mice. PLoS One *7*, e52798.

Thomas, M., Gessner, A., Vornlocher, H.P., Hadwiger, P., Greil, J., and Heidenreich, O. (2005). Targeting MLL-AF4 with short interfering RNAs inhibits clonogenicity and engraftment of t(4;11)-positive human leukemic cells. Blood *106*, 3559-3566.

Wunderlich, M., Brooks, R.A., Panchal, R., Rhyasen, G.W., Danet-Desnoyers, G., and Mulloy, J.C. (2014). OKT3 prevents xenogeneic GVHD and allows reliable xenograft initiation from unfractionated human hematopoietic tissues. Blood *123*, e134-144.

Zweidler-McKay, P.A., He, Y., Xu, L., Rodriguez, C.G., Karnell, F.G., Carpenter, A.C., Aster, J.C., Allman, D., and Pear, W.S. (2005). Notch signaling is a potent inducer of growth arrest and apoptosis in a wide range of B-cell malignancies. Blood *106*, 3898-3906.
